# The selective prolyl hydroxylase inhibitor IOX5 stabilizes HIF-1α and compromises development and progression of acute myeloid leukemia

**DOI:** 10.1038/s43018-024-00761-w

**Published:** 2024-04-18

**Authors:** Hannah Lawson, James P. Holt-Martyn, Vilma Dembitz, Yuka Kabayama, Lydia M. Wang, Aarushi Bellani, Samanpreet Atwal, Nadia Saffoon, Jozef Durko, Louie N. van de Lagemaat, Azzura L. De Pace, Anthony Tumber, Thomas Corner, Eidarus Salah, Christine Arndt, Lennart Brewitz, Matthew Bowen, Louis Dubusse, Derek George, Lewis Allen, Amelie V. Guitart, Tsz Kan Fung, Chi Wai Eric So, Juerg Schwaller, Paolo Gallipoli, Donal O’Carroll, Christopher J. Schofield, Kamil R. Kranc

**Affiliations:** 1 The Institute of Cancer Research, London, UK; 2 Centre for Haemato-Oncology, Barts Cancer Institute, Queen Mary University of London, London, UK; 3 Chemistry Research Laboratory, Department of Chemistry and the Ineos Oxford Institute for Antimicrobial Research, University of Oxford, Oxford, UK; 4 Department of Physiology and Immunology and Croatian Institute for Brain Research, University of Zagreb School of Medicine, Zagreb, Croatia; 5 Centre for Regenerative Medicine, University of Edinburgh, Edinburgh, UK; 6 Université de Bordeaux, Institut National de la Santé et de la Recherche Médicale INSERM U1035, Bordeaux, France; 7 Leukemia and Stem Cell Biology Group, Comprehensive Cancer Centre, King’s College London, London, UK; 8 Department of Haematological Medicine, King’s College Hospital, King’s College London, London, UK; 9 University Children’s Hospital Basel (UKBB), Department of Biomedicine, University of Basel, Basel, Switzerland

**Keywords:** Cancer therapy, Acute myeloid leukaemia, Cancer

## Abstract

Acute myeloid leukemia (AML) is a largely incurable disease, for which new treatments are urgently needed. While leukemogenesis occurs in the hypoxic bone marrow, the therapeutic tractability of the hypoxia-inducible factor (HIF) system remains undefined. Given that inactivation of HIF-1α/HIF-2α promotes AML, a possible clinical strategy is to target the HIF-prolyl hydroxylases (PHDs), which promote HIF-1α/HIF-2α degradation. Here, we reveal that genetic inactivation of *Phd1*/*Phd2* hinders AML initiation and progression, without impacting normal hematopoiesis. We investigated clinically used PHD inhibitors and a new selective PHD inhibitor (IOX5), to stabilize HIF-α in AML cells. PHD inhibition compromises AML in a HIF-1α-dependent manner to disable pro-leukemogenic pathways, re-program metabolism and induce apoptosis, in part via upregulation of BNIP3. Notably, concurrent inhibition of BCL-2 by venetoclax potentiates the anti-leukemic effect of PHD inhibition. Thus, PHD inhibition, with consequent HIF-1α stabilization, is a promising nontoxic strategy for AML, including in combination with venetoclax.

## Abstract (web summary)

Lawson et al. show that genetic inactivation of *Phd1* or *Phd2* hinders progression of AML and compromises leukemic stem cells. They develop a selective PHD inhibitor IOX5 and show therapeutic efficacy in AML, which can be potentiated with venetoclax.

## Main

AML is an aggressive clonal disease of hematopoietic stem cells (HSCs) and primitive progenitors, which acquire diverse mutations to drive disease initiation and progression^1^. Despite recent advances, most AML cases are highly aggressive, with an overall 5-year survival rate of ~30% (ref. ^2^). Hence, there is a clear unmet clinical need to identify new nontoxic therapeutic strategies for improved AML treatment.

Given the hypoxic nature of bone marrow (BM), the functional significance of the α,β-heterodimeric HIF-1 and HIF-2 in AML pathogenesis and the potential of their modulation for AML treatment is of considerable interest. Initial studies proposed that HIF-1 and HIF-2 are required for AML propagation, suggesting an oncogenic role for HIFs in AML^3,4^. Subsequent genetic evidence, however, indicated that inactivation of *Hif1*a and/or *Hif2*a accelerates AML initiation, indicating a tumor-suppressor function for HIFs^5,6^, thus suggesting that one potential therapeutic strategy for AML is to pharmacologically enhance HIF stability.

HIF-α, but not HIF-β, levels are regulated by HIF-prolyl hydroxylase (PHD1–PHD3) catalysis, with PHD2 considered to be the key contributor to setting the steady-state levels of HIF-1α under normoxic conditions^7–10^. Under normal physiological conditions, when O_2_ levels are not limiting, the PHDs catalyze C4-hydroxylation of proline-residues in the α-subunits of HIF-1 and HIF-2 (collectively HIF-α). This post-translational modification strengthens binding of HIF-α to the von Hippel Lindau (VHL) protein, a targeting component of a ubiquitin ligase complex, resulting in HIF-α ubiquitination and subsequent degradation. In hypoxia, PHD activity decreases, and thus HIF-α isoforms are stabilized and translocate to the nucleus where they bind HIF-β and promote transcription of HIF target genes to ameliorate the effects of hypoxia^11–13^. Activation of HIF-mediated gene expression in the absence of hypoxia can be achieved by pharmacological PHD inhibition^14–17^. Small-molecule PHD inhibitors have shown nontoxic therapeutic utility: roxadustat and daprodustat (Dap), inter alia, stimulate erythropoietin production in a HIF-α-dependent manner to enhance erythropoiesis for anemia treatment in patients with chronic renal failure (reviewed previously^8^). Roxadustat has also been shown in murine studies to suppress M2 macrophage polarization to protect from renal fibrosis^18^ and activate phagocytosis in a subset of tumor-infiltrating macrophages to promote their antitumor potential^19^. Furthermore, PHD inhibition enhances the antibacterial activity of skin phagocytes and keratinocytes^20^ and boosts mucosal protection during colitis^21^. Notably, dimethyloxalylglycine (DMOG), a prodrug precursor of *N*-oxalylglycine, which inhibits multiple 2-oxoglutarate (2OG)-dependent oxygenases^22^, including the PHDs, decreases survival of human THP-1 AML cells^23^; however, the therapeutic significance of selective pharmacological PHD inhibition with consequent HIF-α upregulation in many diseases and malignancies, including AML, remains unknown.

Considering that deletion of *Hif1a* and/or *Hif2a* promotes leukemogenesis, we investigated the impact of constitutive HIF activation on AML. We demonstrate that genetic inactivation of *PHD1* and *PHD2* compromises both AML initiation, disease progression, hinders leukemic stem cell (LSC) maintenance, but has no significant impact on multilineage hematopoiesis. We show that a new PHD-selective inhibitor (IOX5), as well as the clinically used PHD inhibitor Dap, potently compromise AML cells in a HIF-dependent manner. From a clinical perspective, while PHD inhibition strongly activates the expression of a number of HIF-α-dependent genes, including pro-apoptotic *BNIP3*, we found that additional inactivation of BCL-2 (by the drug venetoclax), an inhibitor of BAK/BAX-dependent apoptosis^24^, potentiates the anti-leukemic effect of constitutive HIF activation. Thus, our results reveal a promising therapeutic strategy for AML that merits clinical evaluation.

## Results

### PHD2 is required for AML initiation

**Fig. 1:**
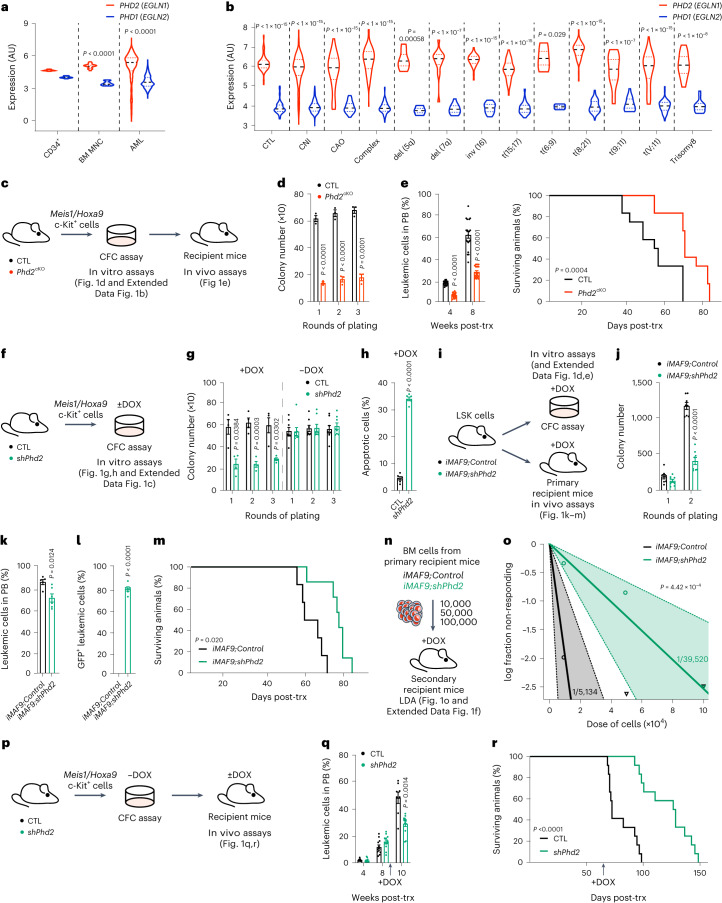
Loss of *Phd2* impairs leukemogenesis in murine AML models. **a**, *PHD2* (*EGLN1*) and *PHD1* (*EGLN2*) expression in human CD34^+^ cells, BM mononuclear cells (MNCs) and samples from patients with AML (for both *EGLN1* and *E*GLN2 expression; CD34^+^, *n* = 16 patients; BM MNCs, *n* = 18 patients; AML, *n* = 671 patients). AU, abitrary units. **b**, *PHD2* (*EGLN1*) and *PHD1* (*EGLN2*) expression in control (CTL), cytologically normal with intermediate prognosis (CNI), cytologically abnormal not otherwise specified (CAO) and different cytogenetic subgroups of human AML BM samples. (CTL, *n* = 198 patients; CNI, *n* = 1,043 patients; CAO, *n* = 47 patients; Complex, *n* = 130 patients; del(5q), *n* = 7 patients; del(7q), *n* = 15 patients; inv(16), *n* = 40 patients; t(15;17), *n* = 54 patients; t(6;9), *n* = 4 patients; t(8;21), *n* = 62 patients; t(9;11), *n* = 19 patients; t(v;11), patients; Trisomy8, *n* = 16 patients). **c**, *Phd2*^fl/fl^ (CTL) and *Phd2*^fl/fl^*;Vav-iCre* (*Phd2*^cKO^) FL c-Kit^+^ cells were co-transduced with *Meis1* and *Hoxa9* retroviruses, serially re-plated in CFC assays and transplanted into lethally irradiated recipient mice. **d**, CFC counts of CTL and *Phd2*^cKO^ cells after each re-plating (*n* = 3 mice per genotype). **e**, Percentage of leukemic cells in the PB of recipient mice (*n* = 20 CTL mice; *n* = 21 *Phd2*^cKO^ mice) and survival curve of mice transplanted with CTL and *Phd2*^cKO^ leukemic cells (*n* = 12 mice per genotype), respectively. trx, transplantation. **f**, *rtTA* (CTL) and *shPhd2/rtTA* (*shPhd2*) leukemic cells were prepared as **c**. CTL and *shPhd2* leukemic cells were serially re-plated in CFC assays. **g**, CFC counts of CTL and *shPhd2* leukemic cells after each re-plating ± DOX (+DOX, *n* = 4 mice per genotype; −DOX, *n* = 8 mice per genotype). **h**, Percentage of annexin V^+^ToPro^+^ cells after three rounds of re-plating +DOX (*n* = 6 mice per genotype). **i**, LSK cells from *iMLL-AF9*;*Control* and *iMLL-AF9;shPhd2* mice were sorted for in vitro and in vivo assays. **j**, CFC counts of *iMLL-AF9;Control* and *iMLL-AF9;shPhd2* cells + DOX (*n* = 8 mice per genotype). **k**, Percentage of leukemic cells in PB of recipient mice 6 weeks following transplantation (*n* = 5 *iMLL-AF9;Control* mice; *n* = 6 *iMLL-AF9;shPhd2* mice). **l**, Percentage of leukemic GFP^+^ cells in PB of recipient mice 6 weeks following transplantation (*n* = 6 mice per genotype). **m**, Survival curve of mice transplanted with *iMLL-AF9;Control* and *iMLL-AF9;shPhd2* LSK cells (*n* = 6 *iMLL-AF9;Control* mice; *n* = 7 *iMLL-AF9;shPhd2* mice). **n**, LDA in secondary recipients transplanted with indicated doses of CD45.2^+^ BM cells from primary recipients. **o**, Plot showing Poisson statistical analysis. Circles represent the percentages of negative mice for each cell dose and triangles represent any data values with zero negative responses. Solid lines indicate the best-fit linear model, and dotted lines represent 95% confidence intervals (CIs). LSC frequencies were calculated using the ELDA software. The exact *n* number per group and analyses from ELDA software are provided in the Source Data. **p**, CTL and *shPhd2* leukemic cells were serially re-plated and transplanted into recipient mice without DOX. At 8 weeks post-transplant, recipient mice were continuously treated with DOX. **q**, Percentage of leukemic cells in PB of recipient mice (*n* = 12 mice per genotype). **r**, Survival curve of mice transplanted with CTL and *shPhd2* leukemic cells (*n* = 12 mice per genotype). Data represent mean ± s.e.m. Comparisons with no *P* value are not significant (NS). *P* values were calculated using a two-tailed Mann–Whitney *U*-test and a paired or unpaired Student’s *t*-test, unless stated otherwise. Kaplan–Meier survival curve statistics were determined using the log-rank (Mantel–Cox) test. Source data

**Extended Data Fig. 1:**
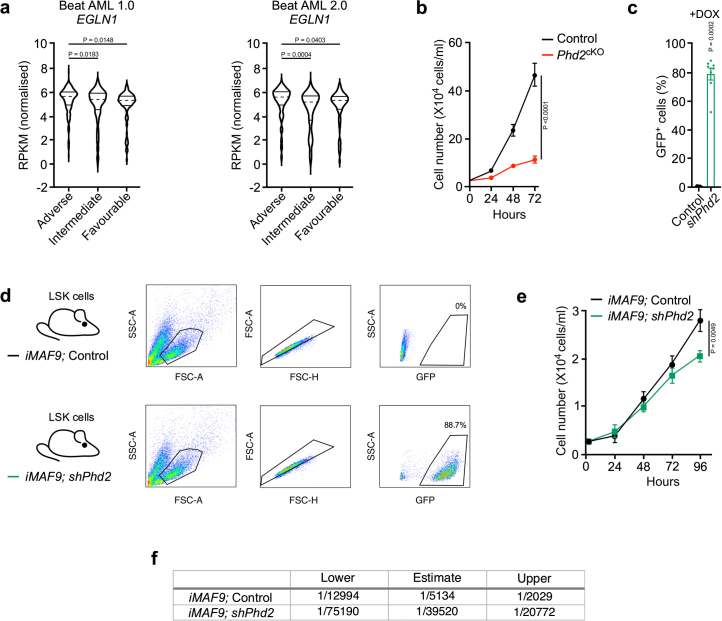
Loss of *Phd2* reduces murine AML cell growth and LSC frequency in vivo*.* **(a)** *PHD2* (*EGLN1*) expression in AML patient samples with adverse, intermediate and favorable prognosis, respectively. Data from Beat AML1.0 and Beat AML 2.0 datasets, respectively. (Beat AML1.0, Adverse n = 162 patients, Intermediate n = 150 patients, Favorable n = 117 patients; Beat AML 2.0, Adverse n = 175 patients, Intermediate n = 100 patients, Favorable n = 158 patients). P values calculated by Ordinary One-way ANOVA. **(b)** Proliferation analyses with Control and *Phd2*^cKO^ cells (n = 6 biological replicates per genotype). P value calculated at 72 hr time point. **(c)** Percentage of GFP^+^ cells after 3 rounds of re-plating +DOX (n = 8 biological replicates per genotype). **(d)** *iMLL-AF9* mice were crossed to Contol (*rtTA*) and *shPhd2* (*shPhd2/rtTA*) mice, generating *iMLL-AF9;Control* and *iMLL-AF9;shPhd2* mice, respectively. LSK cells from *iMLL-AF9;Control* and *iMLL-AF9;shPhd2* mice were cultured in DOX-treated media and GFP expression was measured by flow cytometry. Representative flow cytometry plot (n = 3 mice per genotype). **(e)** Proliferation of *iMLL-AF9;Control* and *iMLL-AF9;shPhd2* cells in the presence of DOX (*iMLL-AF9;Control* n = 9 biological replicates, *iMLL-AF9;shPhd2* n = 12 biological replicates). **(f)** LDA in secondary recipients transplanted with cells from primary recipients (n = 6–7 mice per dose). Lower, estimate and upper LSC frequencies were calculated using the ELDA software. Exact n number per group and analyses from ELDA software is provided in the Source Data. Data represent mean ± SEM. Comparisons not marked with a P value = ns. P values were calculated using a two-tailed Mann–Whitney U-test, paired or un^−^paired student *t*-test, unless stated otherwise. Source data

We investigated the relative abundance of *PHD1* (*EGLN2*) and *PHD2* (*EGLN1*) in normal human BM CD34^+^ cells, BM mononuclear cells from healthy donors and human AML cells. Both *PHD1* and *PHD2* were expressed in all cell populations, with the relative expression of *PHD2* being higher compared to *PHD1* in healthy mononuclear and AML cells (Fig. 1a). Furthermore, while *PHD1* and *PHD2* were largely unchanged in AML subsets with diverse cytogenetic abnormalities compared to non-leukemic controls, the relative *PHD2* expression was overall higher in all AML subsets compared to *PHD1* (Fig. 1b). Notably, the expression level of *PHD2* directly correlated with adverse AML prognosis (Extended Data Fig. 1a).

To investigate the requirement for PHD2 in AML initiation, we employed genetic approaches, beginning with a mouse model of AML driven by expression of *Meis1* and *Hoxa9*, oncogenes that are frequently overexpressed in human AML^25–27^ and which drive leukemogenesis^6^. We combined the *Phd2*^fl^ allele^28^ with the hematopoietic-specific *Vav-iCre* to generate *Phd2*^fl/fl^*;Vav-iCre* (*Phd2*^cKO^) mice. We found that *Phd2*^cKO^ hematopoietic stem and progenitor cells (HSPCs) transduced with *Meis1*/*Hoxa9* retroviruses (Fig. 1c) generated substantially fewer colonies upon serial re-plating (Fig. 1d) and displayed defective proliferation compared to control cells (Extended Data Fig. 1b). Notably, *Phd2*-deficient cells displayed significantly compromised leukemic burden and initiated AML with a longer latency (Fig. 1e), indicating that *Phd2* is required for AML development.

The requirement for PHD2 in leukemic transformation was corroborated by utilizing a mouse model harboring a doxycycline (DOX)-inducible *Phd2* shRNA, coupled with a green fluorescent protein (GFP) reporter (*shPhd2* mice)^29^. Upon DOX treatment, *shPhd2* mice (*CAG-rtTA*^+/−^*TRE-shRNA*^+/−^) express GFP and *shPhd2*, but the control mice (*CAG-rtTA*^+/−^*TRE-shRNA*^−/−^) do not. HSPCs from *shPhd2* and control mice were transduced with *Meis1*/*Hoxa9* retroviruses and serially re-plated in the presence and absence of DOX (Fig. 1f). In the absence of DOX, cells of both genotypes underwent robust serial re-plating; however, when cells of both genotypes were treated with DOX, *shPhd2* cells showed significantly compromised leukemic transformation (Fig. 1g). Furthermore, *shPhd2* activation (evidenced by strong GFP expression) (Extended Data Fig. 1c) induced AML cell apoptosis (Fig. 1h). Taken together, *Phd2* is required for efficient AML cell survival and initiation of disease driven by *Meis1* and *Hoxa9* oncogenes.

To corroborate the requirement for *Phd2* in AML initiation using a distinct AML-driving oncogene, we combined the *shPhd2* system with a DOX-inducible *MLL-AF9* allele (*iMLL-AF9*)^30^, thus enabling activation of mixed-lineage-leukemia (MLL)-AF9 expression concurrently with *Phd2* knockdown (and GFP reporter) upon DOX treatment (Fig. 1i and Extended Data Fig. 1d). We observed that while Lin^–^Sca-1^+^c-Kit^+^ (LSK) HSPCs from *iMLL-AF9;Control* mice undergo efficient transformation upon DOX treatment, those from *iMLL-AF9;shPhd2* mice display defective transformation and compromised proliferative capacity (Fig. 1j and Extended Data Fig. 1e). Thus, PHD2 promotes *MLL-AF9*-driven leukemic transformation in vitro.

To investigate the role of PHD2 in *MLL-AF9*-driven AML in vivo, we transplanted *iMLL-AF9;Control* and *iMLL-AF9;shPhd2* LSK cells into DOX-treated recipient mice (Fig. 1i). We found that *iMLL-AF9* cells with *Phd2* knockdown manifested reduced leukemic engraftment (Fig. 1k,l) and caused AML with substantially increased disease latency compared to control cells (Fig. 1m). To enumerate LSCs in the leukemic recipients of *iMLL-AF9;Control* and *iMLL-AF9;shPhd2* LSK cells, we performed a limiting dilution assay (LDA) with donor-derived CD45.2^+^ BM cells from primary recipients (Fig. 1n). The LSC frequency in recipients of *iMLL-AF9;shPhd2* cells was significantly decreased compared to that in recipients of *iMLL-AF9;Control* cells (Fig. 1o and Extended Data Fig. 1f). Therefore, *Phd2* inactivation compromises LSC development and/or function and hinders MLL-AF9-driven leukemogenesis.

### Acute *Phd2* inactivation impedes progression of established AML

We next investigated whether acute *Phd2* depletion from established AML cells impacts leukemic cell survival and disease outcome. We transformed *shPhd2* and control cells with *Meis1*/*Hoxa9* in the absence of DOX, transplanted the cells into recipient mice and allowed them to establish AML (Fig. 1p,q). Upon detection of leukemic engraftment (Fig. 1q), we continuously administered DOX to induce *Phd2* knockdown. *Phd2* knockdown substantially compromised disease progression and significantly extended mouse survival (Fig. 1q,r). Therefore, genetic inactivation of *Phd2* in newly diagnosed AML curbs disease progression, suggesting PHD inhibition as a promising emerging therapeutic strategy.

### *Phd2* inactivation does not compromise normal multilineage hematopoiesis

**Fig. 2:**
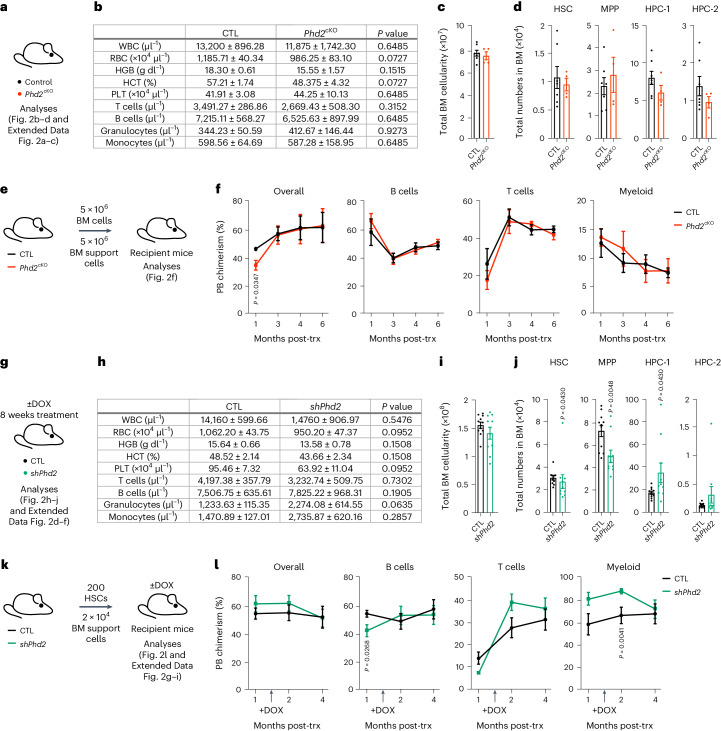
PHD2 is dispensable for functional hematopoiesis. **a**, Steady-state analyses of 8–10-week-old CTL and *Phd2*^cKO^ mice. **b**, PB counts include WBC, white blood cell; RBC, red blood cell; HGB, hemoglobin; HCT, hematocrit; PLT, platelet (*n* = 7 CTL mice; *n* = 4 *Phd2*^cKO^ mice). **c**, Total BM cellularity (*n* = 7 CTL mice; n = 4 *Phd2*^cKO^ mice). **d**, Total numbers of HSCs, MPPs, primitive hematopoietic progenitor cells (HPC-1 and HPC-2) (*n* = 7 CTL mice; *n* = 4 *Phd2*^cKO^ mice). **e**, The 5 × 106 CD45.2^+^ BM cells from 8–10-week-old CTL and *Phd2*^cKO^ mice were transplanted into lethally irradiated syngeneic CD45.1^+^/CD45.2^+^ recipient mice with 5 × 106 competitor CD45.1^+^ BM cells. Animals were analyzed 6 months after transplantation. **f**, Percentage of CD45.2^+^ cells in overall PB, B cell, T cell and myeloid compartments in recipient mice (at all time points *n* = 4 CTL mice; at month 1 and 3, *n* = 5 *Phd2*^cKO^ mice; at month 4 and 6, *n* = 4 *Phd2*^cKO^ mice). **g**, Steady-state analyses of 13–14-week-old CTL and *shPhd2* mice treated with DOX for 8 weeks. **h**, PB counts (*n* = 5 CTL mice; *n* = 5 *shPhd2* mice). **i**, Total BM cellularity (*n* = 11 CTL mice; *n* = 10 *shPhd2* mice). **j**, Total numbers of HSC, MPP, HPC-1 and HPC-2 populations (*n* = 11 CTL mice; *n* = 10 *shPhd2* mice). **k**, 200 HSCs from 8–10-week-old CTL and *shPhd2* mice were transplanted into lethally irradiated syngeneic CD45.1^+^/CD45.2^+^ recipient mice together with 5 × 106 competitor CD45.1^+^ BM cells. Recipient mice were treated with DOX 6 weeks post-transplantation. Animals were analyzed 4 months after transplantation. **l**, Percentage of CD45.2^+^ cells in the overall, B cell, T cell and myeloid PB compartments in recipient mice. For overall engraftment (month 1, *n* = 7 CTL mice, *n* = 9 *shPhd2* mice; month 2, *n* = 8 CTL mice, *n* = 9 *shPhd2* mice; month 4, *n* = 7 CTL mice, *n* = 10 *shPhd2* mice). For myeloid cell engraftment (month 1, *n* = 8 CTL mice, *n* = 9 *shPhd2* mice; month 2, *n* = 9 CTL mice, *n* = 9 *shPhd2* mice; month 4, *n* = 8 CTL mice, *n* = 10 *shPhd2* mice). For B cell engraftment (month 1, *n* = 7 CTL mice, *n* = 12 *shPhd2* mice; month 2, *n* = 7 CTL mice, *n* = 9 *shPhd2* mice; month 4, *n* = 8 CTL mice, *n* = 10 *shPhd2* mice). For T cell engraftment (month 1, *n* = 9 CTL mice, *n* = 12 *shPhd2* mice; month 2, *n* = 8 CTL mice, *n* = 9 *shPhd2* mice; month 4, *n* = 8 CTL mice, *n* = 10 *shPhd2* mice). Data represent mean ± s.e.m. Comparisons with no *P* value are NS. *P* values were calculated using a two-tailed Mann–Whitney *U*-test and paired or unpaired Student’s *t*-test, unless stated otherwise. Source data

**Extended Data Fig. 2:**
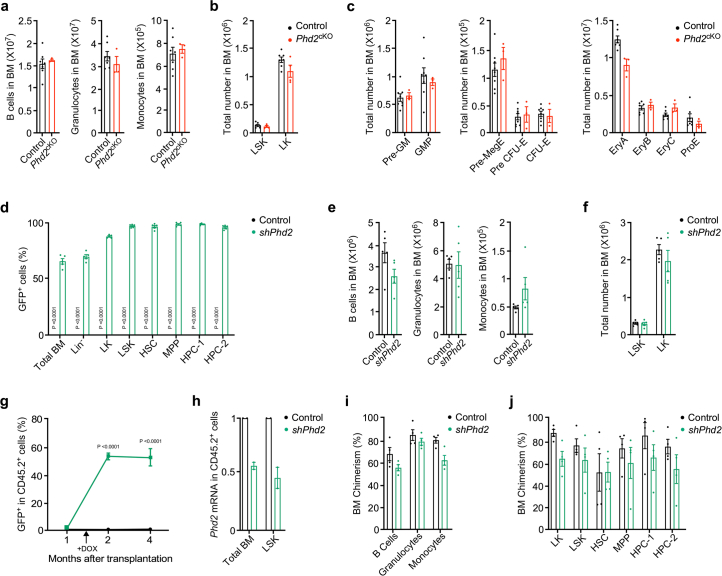
PHD2 is dispensable for functional hematopoiesis. **(a-c)** Analyses of 8^−^ to 10-week-old Control and *Phd2*^cKO^ mice. **(a)** Total numbers of B cells, Granulocytes and Monocytes in the BM (Control n = 7 mice, *Phd2*^cKO^ n = 3 mice). **(b)** Total numbers of LK and LSK cells in the BM (Control n = 7 mice, *Phd2*^cKO^ n = 4 mice). **(c)** Total numbers of Pre-GM, GMP, Pre-MegE, Pre-CFU-E and CFU-E, EryA, EryB, EryC and ProE progenitors (Pre-GM, GMP, Pre-MegE, Pre-CFU-E and CFU-E Control n = 7, *Phd2*^cKO^ n = 3; EryA, EryB, EryC and ProE Control n = 8 mice, *Phd2*^cKO^ n = 3 mice). **(d-f)** Analyses of 13- to 14-week-old Control and *shPhd2* mice treated with DOX for 8 weeks. **(d)** Percentage of GFP^+^ cells in total BM, Lin^-^, LK, LSK, HSC, MPP, HPC-1 and HPC-2 compartments (n = 5 mice per genotype). **(e)** Total numbers of B cells, Granulocytes and Monocytes cells in the BM (n = 5 mice per genotype). **(f)** Total numbers of LK and LSK cells in the BM (n = 5 mice per genotype). **(g-j)** Transplantation of HSCs from 8- to 10-week-old Control and *shPhd2* mice. Recipient mice were treated with DOX 6 weeks post-transplantation. **(g)** Percentage of GFP^+^ cells in CD45.2^+^ in overall PB compartment. (Month 1, n = 7 Control mice; n = 9 *shPhd2* mice. Month 2 n = 10 Control mice; n = 9 *shPhd2* mice. Month 4 n = 9 Control mice; n = 10 *shPhd2* mice). **(h)** Relative levels of *Phd2* mRNA (normalized to β-actin) in total BM and LSK CD45.2^+^ sorted cells 16 weeks post-transplantation (Data analyzed from RNA extracted from n = 2 mice per genotype, plated in triplicate). **(i)** Percentage of CD45.2^+^ cells in the B cell, Granulocytes and Monocytes in the BM compartments of recipient mice (n = 4 mice per genotype). **(j)** The percentage of CD45.2^+^ cells within the LK, LSK, HSC, MPP, HPC-1 and HPC-2 compartments of recipient mice 16 weeks after transplantation (n = 4 mice per genotype). Data represent mean ± SEM. Comparisons not marked with a P value = ns. P values were calculated using a two-tailed Mann–Whitney U-test, paired or unpaired student *t*-test, unless stated otherwise. Source data

Efficient targeting of AML cells without disrupting normal hematopoiesis is required for the development of nontoxic therapies. Notably, *Phd2*^cKO^ mice sustained steady-state hematopoiesis (Fig. 2a,b and Extended Data Fig. 2a), had normal total BM cellularity (Fig. 2c) and displayed unaffected numbers of HSCs and primitive and committed progenitors at different levels of the hematopoietic differentiation hierarchy (Fig. 2d and Extended Data Fig. 2b,c). To test the multilineage reconstitution potential of HSCs lacking *Phd2*, we competitively transplanted BM cells from *Phd2*^cKO^ and control mice into lethally irradiated recipients (Fig. 2e) and found no difference in long-term multilineage hematopoiesis (Fig. 2f). Therefore, deletion of *Phd2* has no impact on steady-state or post-transplantation multilineage hematopoiesis.

We next investigated the consequences of inducible global *Phd2* knockdown on normal hematopoiesis by treating *shPhd2* and control mice with DOX for 8 weeks (Fig. 2g and Extended Data Fig. 2d). Within this timeframe, *Phd2* knockdown had no impact on mouse survival or steady-state multilineage hematopoiesis (Fig. 2h and Extended Data Fig. 2e,f). DOX-treated *shPhd2* mice had unaffected total BM cellularity (Fig. 2i), despite a small reduction in HSC and multipotent progenitor (MPP) numbers (Fig. 2j). To test the fitness of HSCs upon *Phd2* knockdown, we competitively transplanted HSCs from *shPhd2* and control mice (not treated with DOX) into recipients and upon efficient engraftment, we continuously administered DOX for 10 weeks (Fig. 2k and Extended Data Fig. 2g,h). HSCs of both genotypes equally contributed to multilineage hematopoiesis (Fig. 2l and Extended Data Fig. 2i) and stem and progenitor cell compartments of recipient mice (Extended Data Fig. 2j). Thus, inducible *Phd2* knockdown does not impair multilineage hematopoiesis, uncovering a tractable therapeutic window for nontoxic PHD2 inhibition in AML treatment.

### Targeting PHD1 compromises AML but not normal hematopoiesis

**Fig. 3:**
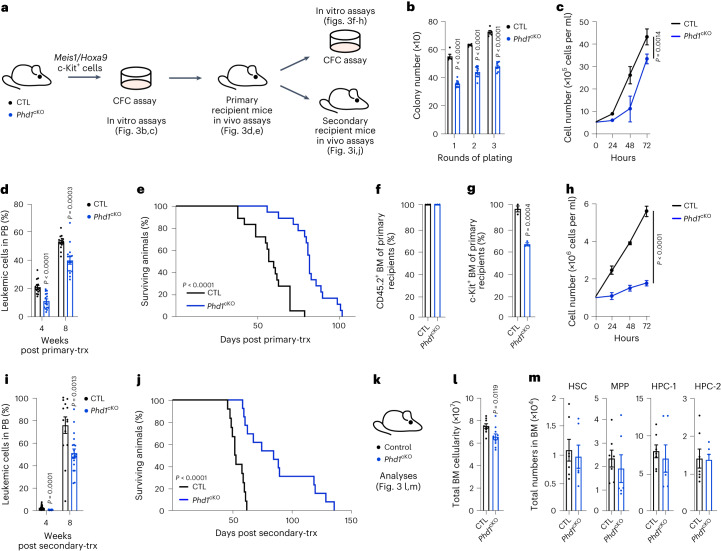
Loss of *Phd1* compromises AML propagation and maintenance. **a***, Phd1*^fl/fl^ (CTL) and *Phd1*^fl/fl^*;Vav-iCre* (*Phd1*^cKO^) FL c-Kit^+^ cells were co-transduced with *Meis1* and *Hoxa9* retroviruses, serially re-plated in CFC assays and transplanted into lethally irradiated recipient mice. Leukemic cells collected from primary recipient mice were then transplanted in lethally irradiated secondary recipients. **b**, CFC counts of CTL and *Phd1*^cKO^ cells after each re-plating (*n* = 5 CTL mice; *n* = 10 *Phd1*^cKO^ mice). **c**, Proliferation analyses with CTL and *Phd1*^cKO^ cells (*n* = 6 CTL biological replicates; *n* = 3 *Phd1*^cKO^ biological replicates). *P* value calculated at the 72-h time point. **d**, Percentage of leukemic cells in the PB of recipient mice in primary transplant (*n* = 15 mice per genotype). **e**, Survival curve of mice transplanted with CTL and *Phd1*^cKO^ leukemic cells in primary transplant (*n* = 18 mice per genotype). **f**, Percentage of CD45.2^+^ leukemic cells in BM of primary recipient mice at the end of the experiment (*n* = 3 mice per genotype). **g**, Percentage of c-Kit^+^ cells in BM of primary recipient mice at the end of the experiment (*n* = 3 mice per genotype). **h**, Proliferation analyses with CTL and *Phd1*^*cKO*^ cells collected from primary recipients (*n* = 6 biological replicates per genotype). *P* value was calculated at the 72-h time point. **i**, Percentage of leukemic cells in the PB of recipient mice in secondary transplant (*n* = 22 mice per genotype). **j**, Survival curve of mice transplanted with CTL and *Phd1*^cKO^ leukemic cells in secondary transplant (*n* = 12 CTL mice; *n* = 13 *Phd1*^cKO^ mice). **k**, Steady-state analyses of 8–10-week-old CTL and *Phd1*^cKO^ mice. **l**, Total BM cellularity (*n* = 9 CTL mice; *n* = 11 *Phd1*^cKO^ mice). **m**, Total numbers of HSCs, MPPs and primitive hematopoietic progenitor cells (HPC-1 and HPC-2) (*n* = 7 CTL mice; *n* = 6 *Phd1*^cKO^ mice). Data represent mean ± s.e.m. Comparisons with no *P* value are NS. *P* values were calculated using a two-tailed Mann–Whitney *U*-test and paired or unpaired Student’s *t*-test, unless stated otherwise. Kaplan–Meier survival curve statistics were determined using the log-rank (Mantel–Cox) test. Source data

Among the three human PHD isoforms, PHD2 is thought to often make the most important contribution to setting the steady-state levels of HIF-1α under normoxia, though both PHD2 and PHD1 isoforms have similar, but not identical, abilities to hydroxylate HIF-α subunits^30^. We thus investigated the functional significance of PHD1 in initiation and propagation of AML driven by *Meis1*/*Hoxa9* (Fig. 3a). Loss of *Phd1* compromised serial re-plating and proliferative potential of *Meis1*/*Hoxa9*-transduced cells (Fig. 3b,c), and impeded AML initiation in vivo (Fig. 3d,e). We then investigated whether *Phd1* loss impacts disease propagation (Fig. 3a). Notably, *Phd1*^cKO^ cells from primary recipients showed reduced c-Kit expression, a marker that enriches for LSCs^31^, and displayed decreased proliferation (Fig. 3f–h). To test the ability of control and *Phd1*-deficient AML cells from primary recipients to propagate AML, we performed secondary transplantation experiments. While transplantation of control cells resulted in aggressive AML in secondary recipients, mice transplanted with *Phd1*^cKO^ AML cells had a reduced leukemic burden, with significantly increased survival (Fig. 3i,j). Thus, PHD1 is required for AML initiation and propagation.

To investigate the role of *Phd1* in multilineage hematopoiesis, we analyzed *Phd1*^cKO^ and control mice under steady-state conditions (Fig. 3k). *Phd1*^cKO^ mice sustained normal hematopoiesis, and despite displaying a reduced cellularity in the BM, loss of *Phd1* had no impact on the HSPC compartment (Fig. 3l,m). Together, as described above for PHD2, PHD1 inactivation impedes AML initiation and propagation without affecting steady-state hematopoiesis.

### Development of a selective PHD inhibitor for AML treatment

**Fig. 4:**
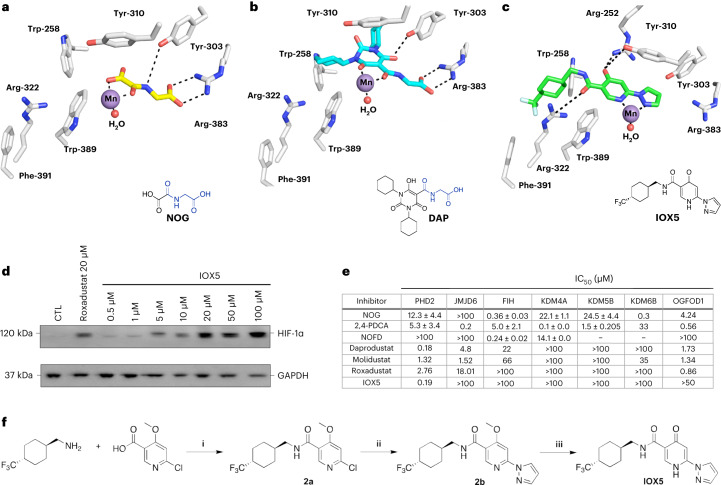
Daprodustat and IOX5 are potent PHD inhibitors with distinct modes of action. **a**, View from a crystal structure of PHD2 complexed with NOG (PDB: 5LR9)^59^. **b**, Visualization of the binding mode of Dap to PHD2, based on a crystal structure of PHD2 complexed with CCT-6 (PDB: 5OX5)^15^. **c**, Visualization of the potential binding mode of IOX5 to PHD2, based on a crystal structure of PHD2 complexed with compound **1** (PDB: 6ST3)^37^. Mn in the crystal structures substitutes for catalytically active Fe(II). **d**, Western blot showing dose-dependent HIF-1α stabilization in HEK293T cells treated with IOX5 or vehicle CTL. GAPDH was used as a loading CTL. Roxadustat was used to achieve HIF-1α stabilization as a positive CTL for the HIF-1α western blot analysis. Blot representative of four independent experiments. **e**, Comparison of the broad-spectrum 2OG oxygenase inhibitors NOG^54^ and 2,4-pyidine dicarboxylic acid (2,4-PDCA) and the FIH selective inhibitor NOFD^54,87,88^ with the PHD-selective inhibitors Dap, molidustat, roxadustat and IOX5 against isolated recombinant forms of 2OG-dependent oxygenases. For conditions see Supplementary Information. **f**, Synthetic route to IOX5. (i) T3P, DIPEA, DMF, room temperature, 16 h, 86%. (ii) Pyrazole, Pd^t^BuXPhos G3, Cs_2_CO_3_, ^t^BuOH, N_2_, 60 °C, 16 h, 46%. (iii) Lithium chloride (LiCl), DMAc, 120 °C, microwave, 8 h, 30%. Source data

**Extended Data Fig. 3:**
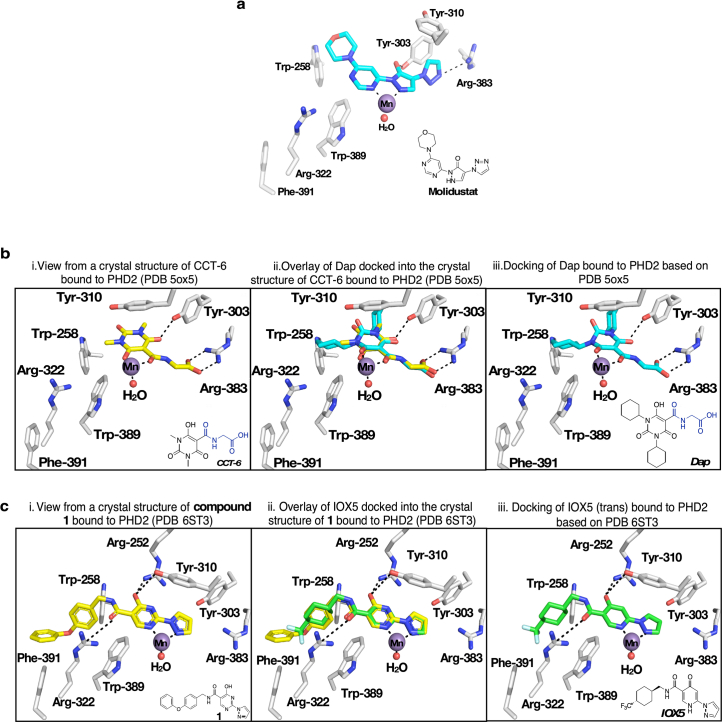
Modeling of PHD inhibitors. **(a)** View from a crystal structure of PHD2 complexed with Molidustat (PDB: 6ZBO)^17^. **(b)** Visualization of Dap complexed with PHD2. (i) View of PHD2 complexed with CCT-6 (PDB: 5ox5) and structure of CCT-6. (ii) An overlay of binding mode of Dap (modeled) with CCT-6. (iii) Prediction of Dap binding mode with PHD2 (PDB: 5ox5) and structure of Dap. **(c)** Visualization of IOX5 complexed with PHD2. (i) View of PHD2 complexed with **compound 1** (PDB: 6ST3) and the chemical structure of **compound 1**. (ii) An overlay of binding mode of IOX5 (modeled) with that of **compound 1**. (iii) Prediction of IOX5 binding mode with PHD2 (PDB: 6ST3) and structure of IOX5. Note Mn in the crystal structures substitutes for catalytically active Fe(II).

We next investigated whether PHD inhibition by small molecules can be deployed for therapeutic purposes in AML. The PHDs belong to the 2OG-dependent oxygenase superfamily. There are 60–70 human 2OG oxygenases, which, inter alia, have roles in collagen biosynthesis, lipid metabolism, DNA damage repair, epigenetic regulation and messenger RNA modification^7^. Some 2OG oxygenases have reported roles in AML, including the JmjC KDM5 subfamily, which catalyze demethylation of *N*-methyl groups of lysine and arginine residues on histones^32–34^. Notably, the key elements of 2OG binding and Fe(II) coordination are substantially, but incompletely, conserved in PHD1–PHD3 and many structurally related human 2OG oxygenases^7^, including the HIF-α asparaginyl hydroxylase (factor inhibiting HIF (FIH)), catalysis by which suppresses transcription of HIF target genes in a context-dependent manner^35,36^. Current clinically used PHD inhibitors, including Dap, roxadustat and molidustat bind in the 2OG co-substrate binding pocket and chelate the active site Fe(II) of the PHDs, similarly to *N*-oxalylglycine (NOG), a broad-spectrum 2OG oxygenase inhibitor (close 2OG analog) (Fig. 4a,b,e and Extended Data Fig. 3a,b)^15,17,22^. A pharmacological mode of action relying solely on 2OG competition has potential off target effects, including inhibition of other 2OG-dependent oxygenases, as well as of other 2OG using enzymes. Dap and molidustat inhibit Jumonji domain containing 6, arginine demethylase and lysine hydroxylase (JMJD6) (half-maximum inhibitory concentration (IC_50_) of 4.8 and 1.52 μM, respectively), whereas roxadustat weakly inhibits JMJD6 (IC_50_ of 18.01 μM) (Fig. 4e). Dap, molidustat and roxadustat both inhibit 2-oxoglutarate and iron-dependent oxygenase domain containing 1 (OGFOD1) (IC_50_ 1.73, 1.34 and 0.86 μM, respectively), which, like the PHDs, is a prolyl hydroxylase. Dap manifests weak inhibition of FIH (IC_50_ of 22 μM) and molidustat weakly inhibits KDM6B (IC_50_ of 35 μM) (Fig. 4e)^15^. Considering the large number of 2OG-dependent oxygenases, and their broad functional roles in various fundamental biological processes, we considered it important to develop a highly selective PHD inhibitor suitable for use in AML therapy.

**Extended Data Fig. 4:**
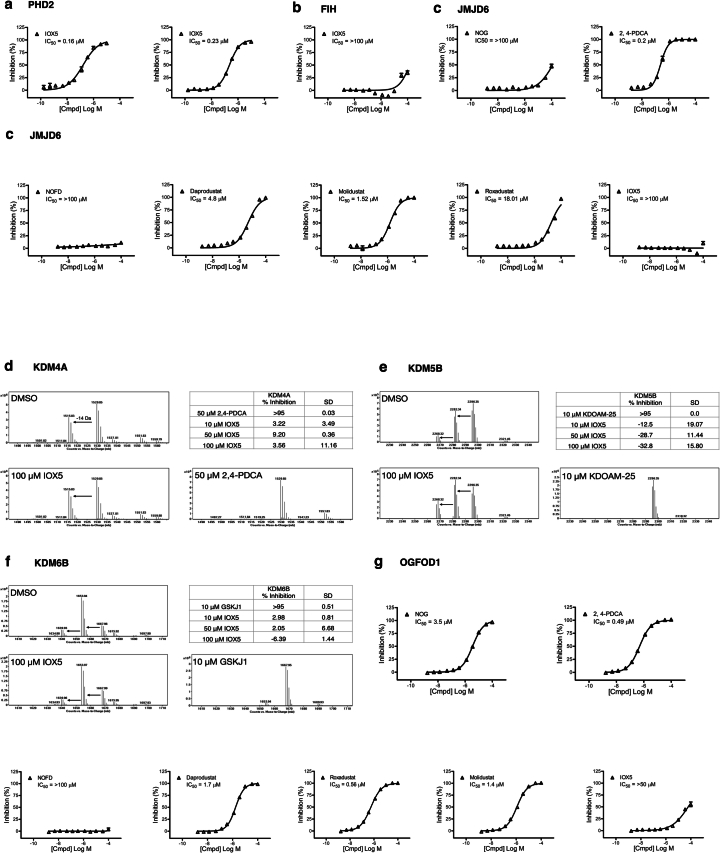
Inhibition curves of PHD inhibitors. **(a)** PHD2 inhibition by IOX5 as assayed by SPE-MS^35^. No enzyme and DMSO controls were used for normalization of the HIF-1α CODD peptide hydroxylation. Errors are standard deviations of the mean (n = 2 independent assays). Eleven concentrations assayed in duplicate were used to obtain dose-response curve for IOX5. Values in the graph are means with standard deviation (n = 2 independent assays). IC_50_ values were calculated using GraphPad Prism. **(b)** in vitro FIH selectivity profiling of IOX5, as assayed by SPE-MS^15^. Eleven concentrations assayed in duplicate were used to obtain a dose-response curve for IOX5. Values present in the graph are means with standard deviation (n = 2 independent assays). IC_50_ values were calculated using GraphPad Prism. **(c)** in vitro JMJD6 selectivity profiling of N-oxalylglycine (NOG), 2,4-pyridine dicarboxylic acid (2,4-PDCA) and the FIH selective inhibitor N-oxalyl-D-phenylalanine (NOFD), Daprodustat, Molidustat, Roxadustat and IOX5 assayed by SPE-MS. Eleven concentrations assayed in duplicate were used to obtain dose-response curves. IC_50_ values were calculated using GraphPad Prism (n = 2 independent assays). **(d)** in vitro KDM4A selectivity profiling of IOX5, as assayed by LCMS. Three concentrations were assayed in duplicate. **(e)** in vitro KDM5B selectivity profiling of IOX5, as assayed by LCMS^15^. Three concentrations were assayed in duplicate. **(f)** in vitro KDM6B selectivity profiling of IOX5, as assayed by LCMS. Three concentrations were assayed in duplicate. **(g)** OGFOD1 selectivity profiling of N-oxalylglycine (NOG), 2,4-pyridine dicarboxylic acid (2,4-PDCA) and the FIH selective inhibitor N-oxalyl-D-phenylalanine (NOFD), Daprodustat, Molidustat, Roxadustat and IOX5 as assayed by SPE-MS (the protocol is described in the Supplemental Information section). Eleven concentrations assayed in duplicate were used to obtain dose-response curves for each compound. IC_50_ values were calculated using GraphPad Prism (n = 2 independent assays). Source data

With the aim of developing a PHD inhibitor suitable for AML treatment, we identified a PHD-specific hydrophobic pocket close to the entrance of the PHD2 active site and proposed that targeting this pocket may lead to PHD-selective inhibition. Crystallographic analysis of PHD2 complexed with a 4-hydroxypyrimidine inhibitor (**1**; Extended Data Fig. 3c) implied that binding in this pocket can help enable potent PHD inhibition^37^; however, **1** also potently inhibited other 2OG oxygenases such as the human prolyl hydroxylase OGFOD1 and a viral collagen prolyl hydroxylase (vCPH); further **1** only stabilized HIF-1α at 100 μM in Hep3b cells^37^. To improve the selectivity and cellular efficacy of **1**, we conducted studies to improve on its interaction with the PHD-specific pocket and altering interactions within the 2OG binding pocket; these resulted in development of IOX5 (Fig. 4c). Docking studies on the IOX5-binding mode, based on the structure of PHD2 complexed with **1** (Extended Data Fig. 3c)^37^, imply that IOX5 binds to the PHD2 (and likely PHD1 and PHD3) active site metal (within the 2OG binding pocket) via chelation of its pyridone and pyrazole groups. The IOX5 pyridone group can hydrogen bond with Tyr-310 and Arg-252, and its 4-trifluoromethyl substituted cyclohexyl group binds in the PHD-specific hydrophobic pocket at the active site entrance (Fig. 4c and Extended Data Fig. 3c). Notably, like Dap (IC_50_ of 0.18 μM), IOX5 is a highly potent PHD2 inhibitor (IC_50_ of 0.19 μM with isolated PHD2) (Fig. 4e and Extended Data Fig. 4a), with molidustat and roxadustat (IC_50_ of 1.32 and 2.76 μM, respectively) being somewhat less potent under our assay conditions^15^ (Fig. 4e). IOX5 was shown to compete with 2OG for binding to PHD2 (Supplementary Fig. 1). Moreover, IOX5 stabilizes HIF-1α at 0.5 μM in HEK293T cells (Fig. 4d), indicating, at least, equivalent cellular potency compared to the existing clinically used PHD inhibitors and **1** (ref. ^37^).

Of note, IOX5 did not inhibit any of the other tested human 2OG-dependent oxygenases, including FIH and the histone-modifying 2OG oxygenases lysine-specific demethylase 4A (KDM4A), lysine-specific demethylase 5B (KDM5B), lysine-specific demethylase 6B (KDM6B), JMJD6 at 100 μM and the prolyl hydroxylase OGFOD1 at 50 μM, contrasting with Dap, roxadustat and molidustat^15,38^ (Fig. 4e and Extended Data Fig. 4b–g). The lack of activity of IOX5 against FIH is notable as catalysis by FIH regulates HIF in a context-dependent manner^36^. Thus, both Dap and IOX5 are similarly potent PHD inhibitors; however, IOX5 is more selective.

### PHD inhibition stabilizes HIF-1α and promotes apoptosis

**Fig. 5:**
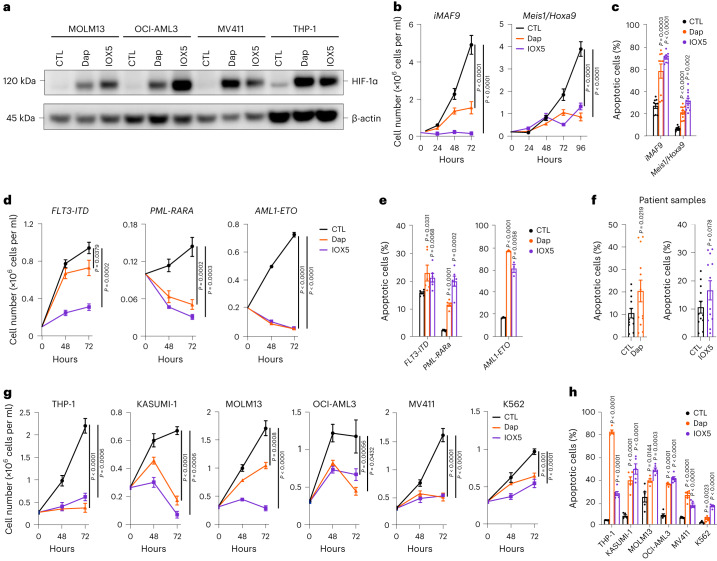
Pharmacological inhibition of PHD2 stabilizes HIF-1α and substantially impacts AML growth and survival in vitro*.* **a**, Western blot of HIF-1α in MOLM13, OCI-AML3, MV411 and THP-1 cells treated with Dap, IOX5 or vehicle CTL for 48 h. β-Actin was used as a loading control. Blot is representative of three independent experiments. **b**, Proliferation of *iMLL-AF9*- and *Meis1*/*Hoxa9*-transformed murine cells treated with Dap, IOX5 or vehicle CTL (*iMLL-AF9;* *n* = 9 biological replicates per group. *Meis1*/*Hoxa9;* *n* = 12 biological replicates per group). *P* values calculated at 72-h and 96-h time points, respectively. **c**, Annexin V^+^DAPI^+^ analyses of *iMLL-AF9*- and *Meis1*/*Hoxa9*-transformed murine cells treated with Dap, IOX5 or vehicle CTL (*n* numbers as in **b**). **d**, Proliferation analyses of *FLT3-ITD-*, *PML-RARα-* and *AML1-ETO-*transformed murine cells treated with Dap, IOX5 or vehicle CTL (*FLT3-ITD* *n* = 6 biological replicates per group; *PML-RARα* *n* = 6 biological replicates per group; *AML1-ETO* *n* = 3 biological replicates per group). *P* values calculated at the 72-h time point. **e**, Annexin V^+^DAPI^+^ analyses of *FLT3-ITD-*, *PML-RARα-* and *AML1-ETO-*transformed murine cells treated with Dap, IOX5 or vehicle CTL (*FLT3-ITD* *n* = 6 biological replicates per group; *PML-RARα,* *n* = 6 biological replicates per group; *AML1-ETO*, *n* = 3 biological replicates per group). **f**, Percentage of annexin V^+^PI^+^ cells (matched to vehicle CTL) of individual patient samples treated with Dap or IOX5 (*n* = 12 patients). **g**, Proliferation analyses of THP-1, KASUMI-1, MOLM13, OCI-AML3, MV411 and K562 cells treated with Dap, IOX5 or vehicle CTL (THP-1: CTL, 0 h *n* = 12 biological replicates, 48 h *n* = 8 biological replicates, 72 h *n* = 3 biological replicates; Dap, *n* = 6 biological replicates per time point; IOX5, 0 h *n* = 12 biological replicates, 48 h *n* = 9 biological replicates, 72 h *n* = 3 biological replicates; KASUMI-1, *n* = 6 biological replicates per group; MOLM13, *n* = 6 biological replicates per group; OCI-AML3, *n* = 6 biological replicates per group; MV411, *n* = 6 biological replicates per group; K562, *n* = 6 biological replicates per group). *P* values calculated at the 72-h time point. **h**, Annexin V^+^DAPI^+^ analyses of THP-1, KASUMI-1, MOLM13, OCI-AML3, MV411 and K562 cells treated with Dap, IOX5 or vehicle CTL (THP-1: CTL *n* = 6 biological replicates, Dap *n* = 5 biological replicates, IOX5 *n* = 6 biological replicates; KASUMI-1: CTL *n* = 5 biological replicates, Dap *n* = 5 biological replicates IOX5, *n* = 6 biological replicates; MOLM13: CTL *n* = 5 biological replicates, Dap *n* = 5 biological replicates, IOX5 *n* = 6 biological replicates; OCI-AML3: *n* = 6 biological replicates per group; MV411: CTL *n* = 6 biological replicates, Dap *n* = 6 biological replicates, IOX5 *n* = 5 biological replicates; K562: CTL *n* = 6 biological replicates, Dap *n* = 6 biological replicates, IOX5 *n* = 5 biological replicates). THP-1 = *MLL-AF9* translocation; KASUMI-1 = *AML-ETO* translocation; MOLM13 = *MLL-AF9* translocation and *FLT3-ITD* mutation; OCI-AML3 = *DNMT3A*, *NRAS* and *NPM1* mutations; MV411 = MLL-AF4 and *FLT3-ITD* mutation; K562 = *BCR*-ABL translocation. Data represent mean ± s.e.m. Comparisons with no *P* value are NS. *P* values were calculated using a two-tailed paired Student’s *t*-test, unless stated otherwise. Source data

**Extended Data Fig. 5:**
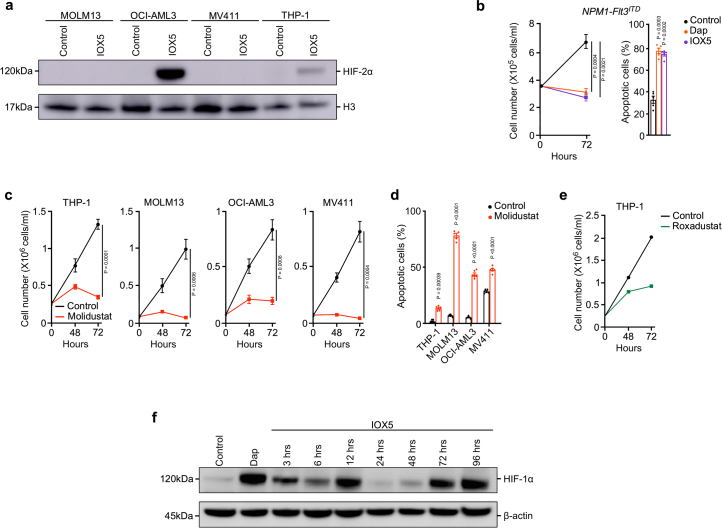
Therapeutic validation of PHD inhibitors in AML. **(a)** Western blot of HIF-2α in MOLM13, OCI-AML3, MV411 and THP-1 cells treated with IOX5 or vehicle control for 48hrs. α-Histone 3 (H3) used as a loading control. Blot is representative of 3 independent experiments. **(b)** Proliferation and Annexin-V^+^DAPI^+^ analyses of murine *NPM1/Flt3-ITD* cells treated with Daprodustat (Dap), IOX5 or vehicle control (n = 5 biological replicates per group). **(c)** Proliferation analyses of THP-1, MOLM13, OCI-AML3, MV411 cells treated with Molidustat (Mol) or vehicle control (THP-1 n = 5 biological replicates per group; MOLM13, OCI-AML3 and MV411 n = 6 biological replicates per group). P value calculated at 72 hr time point. **(d)** Annexin-V^+^DAPI^+^ analyses of THP-1, MOLM13, OCI-AML3, MV411 cells treated with Molidustat or vehicle control. (THP-1 Control n = 10 biological replicates, Mol n = 5 biological replicates; MOLM13 Control n = 6 biological replicates, Mol n = 5 biological replicates, OCI-AML3 n = 6 biological replicates per group, MV411 n = 6 biological replicates per group). **(e)** Proliferation analyses of THP-1 cells treated with Roxadustat or vehicle control (Control n = 3 biological replicates, Roxadustat n = 2 biological replicates). **(f)** Western blot of HIF-1α in THP-1 cells treated with IOX5 for described time points. For vehicle control and positive control, cells were treated with DMSO and Dap, respectively, for either 48 or 96 hrs. β-actin used as a loading control. Blot representative of 2 independent experiments. Data represent mean ± SEM. Comparisons not marked with a P value = ns. P values were calculated using a two-tailed Mann–Whitney U-test, paired or unpaired student *t*-test, unless stated otherwise. Source data

We next investigated the impact of pharmacological PHD inhibition on AML cells, using both IOX5 and Dap, the latter of which has entered clinical practice for treatment of anemia in chronic kidney disease^8^. As observed with HEK293T cells^15^, treatment with either Dap or IOX5 consistently stabilized HIF-1α in a range of human AML cell lines (Fig. 5a). Furthermore, IOX5 stabilized HIF-2α protein levels in some but not all AML cell lines (Extended Data Fig. 5a). Thus, while PHD inhibition consistently stabilizes HIF-1α protein across all tested AML cell lines, HIF-2α stabilization is variable and cell line-specific.

Given the anti-leukemic effect of genetic *Phd1*/2 inactivation on murine AML cells, we treated murine *iMLL-AF9-* and *Meis1/Hoxa9*-transformed cells with Dap or IOX5. Both compounds substantially reduced the proliferative capacity and induced apoptosis in these cells (Fig. 5b,c). To validate the anti-leukemic effect of PHD inhibition on AML cells transformed by drivers independent of the *MLL*/*Meis1*/*Hoxa9* axis, we employed murine AML cells harboring *FLT3-ITD* mutations (with and without *NPM1* mutations), *PML-RARα* and *AML1-ETO*. We found that Dap and IOX5 reduced the proliferative rate and induced apoptosis in these cells (Fig. 5d,e and Extended Data Fig. 5b).

To investigate the clinical utility of PHD inhibition for AML treatment, we determined the impact of Dap and IOX5 on AML patient samples, focusing on poor-risk AML, which is characterized by a particularly low overall patient survival rate^2^. We found that treatment of independent patient samples with Dap or IOX5 clearly increased apoptosis of primary human AML cells (Fig. 5f and Supplementary Table 1).

To further investigate the consequences of PHD inhibition, we tested a range of human AML cell lines with diverse mutational backgrounds (detailed in Fig. 5 legend) and found, in accord with our results in murine AML cells, that Dap or IOX5 treatment consistently compromised their proliferation and survival (Fig. 5g,h). Furthermore, other chemically distinct PHD inhibitors, molidustat and roxadustat, also manifested strong anti-leukemic activity in established human AML cells (Extended Data Fig. 5c–e).

We next tested whether prolonged PHD inhibition in AML cells stabilizes HIF-1α transiently or in a sustained manner to compromise AML cells. We continuously treated THP-1 AML cells with IOX5 for 96 h and found that HIF-1α was stabilized after 3–12 h; its levels decreased between 24–48 h, then increased 72–96 h after treatment initiation (Extended Data Fig. 5f). This finding is consistent with previous reports indicating that HIF-1α levels peak and subsequently decline upon prolonged hypoxic exposure^39,40^ and the fact that HIF activity may oscillate^41^. While future investigations are warranted to provide mechanistic details underpinning this expression pattern, we conclude that IOX5-induced HIF-1α stabilization is sufficient to compromise AML cells.

### PHD inhibition impairs LSCs, extending survival of leukemic mice

**Fig. 6:**
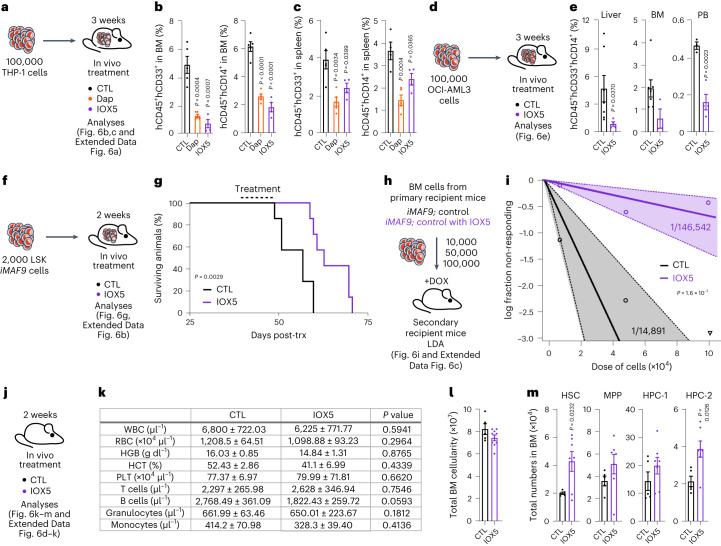
PHD inhibition decreases AML engraftment and increases survival in vivo but does not affect normal hematopoiesis. **a**, A total of 100,000 THP-1 cells were transplanted into NBSGW recipient mice. At 14 days following transplantation, recipient mice were treated with Dap, IOX5 or vehicle CTL 2× daily via i.p. injection for 21 days. **b**, Percentage of human CD45^+^CD33^+^ and human CD45^+^CD14^+^ cells in the BM (vehicle CTL-treated mice *n* = 5; Dap-treated mice *n* = 5; IOX5-treated mice *n* = 4). **c**, Percentage of human CD45^+^CD33^+^ and human CD45^+^CD14^+^ cells in the spleen (vehicle CTL-treated mice *n* = 4–5; Dap-treated mice *n* = 5; IOX5-treated mice *n* = 4). **d**, A total of 100,000 OCI-AML3 cells were transplanted into NBSGW recipient mice. At 14 days following transplantation, recipient mice were treated with IOX5 or vehicle CTL 2× daily via i.p. injection for 21 days. **e**, Percentage of human CD45^+^CD33^+^CD14^+^ cells in the liver, BM and PB, respectively (liver: *n* = 7 vehicle CTL-treated mice, *n* = 4 IOX5-treated mice; BM: *n* = 7 vehicle CTL-treated mice, *n* = 3 IOX5-treated mice; PB: *n* = 3 vehicle CTL-treated mice, *n* = 4 IOX5-treated mice). **f**, A total of 2,000 LSK cells from *iMLL-AF9;Control* mice were sorted and transplanted into irradiated recipient mice. At 40 days following transplantation, recipient mice were treated with IOX5 or vehicle CTL 2× daily via i.p. injection for 14 days. **g**, Survival curve of mice transplanted with *iMLL-AF9;Control* LSK cells treated with IOX5 or a vehicle CTL (*n* = 7 mice per group). **h**, LDA in secondary recipients transplanted with indicated doses of CD45.2^+^ BM cells from primary recipients. **i**, Plot showing Poisson statistical analysis. Circles represent the percentages of negative mice for each cell dose, triangles represent any data values with zero negative responses. Solid lines indicate the best-fit linear model and dotted lines represent 95% CIs. LSC frequencies were calculated using the ELDA software. The exact *n* number per group and analyses from ELDA software are provided in the Source Data. **j**, Steady-state analyses of 8–10-week-old C57Bl6 mice treated with IOX5 or vehicle CTL 2× daily via i.p. injection for 14 days. **k**, PB counts (*n* = 6 vehicle CTL-treated mice; *n* = 8 IOX5-treated mice). **l**, Total BM cellularity (*n* = 5 vehicle CTL-treated mice; *n* = 9 IOX5-treated mice). **m**, Total numbers of HSC, MPP, HPC-1 and HPC-2 populations (*n* = 5 vehicle CTL-treated mice; *n* = 7 IOX5-treated mice). Data represent mean ± s.e.m. Comparisons with no *P* value are NS. *P* values were calculated using a two-tailed Mann–Whitney *U*-test and paired or unpaired Student’s *t*-test, unless stated otherwise. Kaplan–Meier survival curve statistics were determined using the log-rank (Mantel–Cox) test. Source data

**Extended Data Fig. 6:**
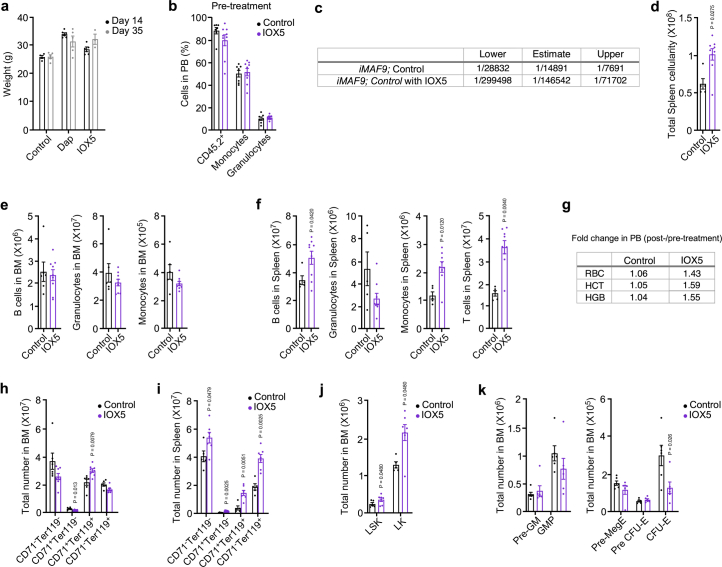
Anti-leukemic potential of PHD inhibition in vivo*.* **(a)** Weight of animals pre- and post-treatment with Dap, IOX5 or vehicle control 2x daily via i.p. injection for 21 days. (Day 14, Control n = 4 mice, Dap n = 6 mice, IOX5 n = 6 mice; Day 35, Control n = 5 mice, Dap n = 5 mice, IOX5 n = 3 mice). **(b)** Percentage of leukemic CD45.2^+^ cells and myeloid cells in PB of recipient mice pre-treatment with IOX5 or vehicle control (Control n = 7 mice, IOX5 n = 9 mice). **(c)** LDA analyses in secondary recipients transplanted with cells from primary recipients (n = 9–10 mice per dose). Lower, estimate and upper LSC frequencies were calculated using the ELDA software. **(d-k)** Analyses of 8- to 10-week-old C57Bl6 mice treated with IOX5 or vehicle control 2x daily via i.p. injection for 14 days. **(d)** Total spleen cellularity (Vehicle control-treated mice n = 5, IOX5-treated mice n = 9). **(e)** Total numbers of B cells, Granulocytes and Monocytes in the BM (Vehicle control-treated mice n = 6, IOX5-treated mice n = 9). **(f)** Total numbers of B cells, Granulocytes, Monocytes and T cells in the spleen (Vehicle control-treated mice n = 5, IOX5-treated mice n = 9). **(g)** Fold change of RBC, HCT and HGB in PB post-/pre-treatment (Vehicle control-treated mice n = 6, IOX5-treated mice n = 8). **(h)** Total numbers of erythrocyte progenitors in the BM (Vehicle control-treated mice n = 6, IOX5-treated n = 8). **(i)** Total numbers of erythrocyte progenitors in the spleen (Vehicle control-treated mice n = 5, IOX5-treated mice n = 7). **(j)** Total numbers of LSK and LK cells in the BM. (Vehicle control-treated mice n = 5 mice, IOX5-treated mice n = 7). **(k)** Total numbers of Pre-GM, GMP, Pre-MegE, Pre-CFU-E and CFU-E progenitors in the BM (n = 6 mice per group). Data represent mean ± SEM. Comparisons not marked with a P value = ns. P values were calculated using a two-tailed Mann–Whitney U-test, paired or unpaired student *t*-test, unless stated otherwise. Source data

To test the anti-leukemic potential of Dap and IOX5 in vivo, we administered them to mice engrafted with human THP-1 cells (harboring an *MLL-AF9* translocation) and found that the treatment was well tolerated and significantly reduced leukemic burden (Fig. 6a–c and Extended Data Fig. 6a). Next, we examined whether IOX5 has a therapeutic impact on MLL-AF9-independent AML. Following engraftment of recipients with OCI-AML3 cells (harboring *DNMT3A*, *NRAS* and *NPM1* mutations), we administered IOX5 (Fig. 6d). This significantly reduced the leukemic burden (Fig. 6e). Therefore, IOX5 compromises both MLL-rearranged and non-MLL AML in vivo.

To further examine the translational utility of IOX5 in AML, we transplanted murine *iMLL-AF9* LSK cells into DOX-treated recipient mice, and following confirmation of equal engraftment, we treated mice with IOX5 for 14 days (Fig. 6f and Extended Data Fig. 6b). IOX5 substantially increased survival of leukemic mice compared to those treated with vehicle (Fig. 6g). Furthermore, a LDA revealed that IOX5-treated recipients had significantly decreased LSC frequency (Fig. 6h,i and Extended Data Fig. 6c), indicating that PHD inactivation compromises LSC maintenance and AML progression.

Given that Dap has been deemed safe in murine and human studies^8,42^, we examined the impact of IOX5 on normal hematopoiesis. We found that acute PHD inhibition with IOX5 had no detrimental impact on the differentiated PB, BM or spleen cell compartments (Fig. 6j–l and Extended Data Fig. 6d–f) and, as expected, enhanced erythropoiesis (Extended Data Fig. 6g–i). Moreover, IOX5 treatment manifested no significant defects in the HSPC compartment (Fig. 6m and Extended Data Fig. 6j,k). Therefore, reflecting our genetic studies, chemically distinct small-molecule PHD inhibitors, with related but distinct PHD binding modes, display strong anti-leukemic activity, but do not disrupt normal hematopoiesis.

### IOX5 compromises AML in a HIF-α dependent manner

**Fig. 7:**
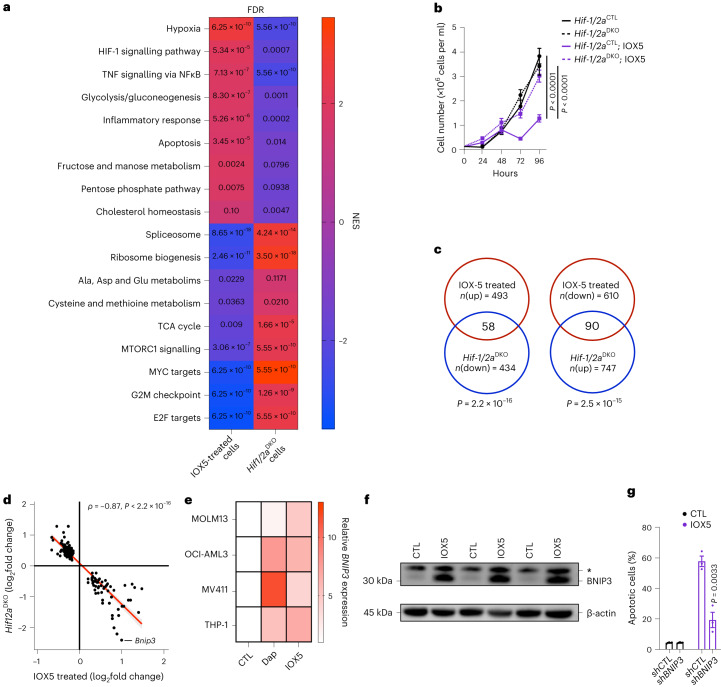
Targeting PHD2 upregulates HIF target genes, including the pro-apoptotic *BNIP3.* **a**, GSEA showing pathways up- and downregulated in IOX5-treated *iMLL-AF9* and *Hif1/2a*^DKO^ *Meis1*/*Hoxa9* cells (*n* = 4 RNA samples per group). False discovery rate (FDR) displayed on the graph. **b**, Proliferation of *Hif1/2a*^CTL^ and *Hif1/2a*^DKO^ cells treated with IOX5 or vehicle CTL (all *n* = 12 biological replicates per group apart from: *Hif1/2a*^CTL^ treated with vehicle CTL at 72 h *n* = 11; H*if1/2a*^DKO^ treated with IOX5 at 24 h *n* = 9, 48 h *n* = 9, 72 h *n* = 9, 96 h *n* = 8). *P* values calculated at the 96-h time point. **c**, Venn diagram of overlapping genes between up- and downregulated genes in IOX5-treated *iMLL-AF9* and *Hif1/2a*^DKO^ *Meis1*/*Hoxa9* cells. Fisher’s exact test statistical analyses shown. Odds ratio 4.04 and 2.85, respectively. **d**, Scatter-plot showing the inverse correlation between up- and downregulated transcripts as shown in **c**. Statistical significance was evaluated using Spearman’s correlation test. *Bnip3* is annotated. **e**, Relative levels of *BNIP3* mRNA (normalized to *ACTB*) in MOLM13, OCI-AML3, MV411 and THP-1 cells treated with Dap, IOX5 or vehicle CTL. RNA extracted from individual biological replicates and plated in triplicate. (MOLM13: CTL *n* = 4 biological replicates, Dap *n* = 4 biological replicates, IOX5 *n* = 3 biological replicates; OCI-AML3: *n* = 3 biological replicates; MV411: *n* = 3 biological replicates; THP-1: *n* = 3 biological replicates) **f**, Western blot of BNIP3 in THP-1 cells treated with IOX5 or vehicle CTL. β-actin used as a loading CTL. Asterisk indicates a nonspecific band. Blot is representative of two independent experiments. **g**, MOLM13 cells were transduced with lentiviruses expressing scrambled short hairpin RNA (*shCTL*) and a shRNA targeting *BNIP3* (*shBNIP3*). Annexin V^+^DAPI^+^ analyses of *shCTL* and *shBNIP3* MOLM13 cells treated with IOX5 or vehicle CTL (*n* = 3 biological replicates per group). Data represent mean ± s.e.m. Comparisons with no *P* value are NS. *P* values were calculated using a two-tailed paired or unpaired Student’s *t*-test, unless stated otherwise. Source data

**Extended Data Fig. 7:**
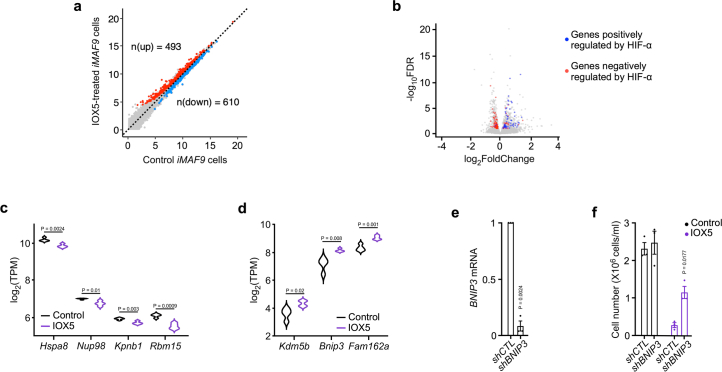
Dysregulated transcripts following PHD inhibition. **(a)** Scatter-plot of transcripts in control and IOX5-treated *iMLL-AF9* cells (n = 4 RNA samples per group). Transcripts significantly up- (red) and downregulated (blue) in IOX5-treated *iMLL-AF9* cells are highlighted. **(b)** Volcano plot of differentially expressed genes in IOX5-treated *iMLL-AF9* cells. Genes positively- and negatively- regulated by HIF-1α^12^ are marked in blue and red, respectively. **(c)** Expression of *Hspa8*, *Nup98*, *Kpnb1* and *Rbm15* in IOX5-treated *iMLL-AF9* cells. Violin plots showing distribution of log2 expression values (transcripts per million). (n = 4 RNA samples per group). **(d)** Gene expression of *Kdm5b*, *Bnip3*, and *Fam162a* in IOX5-treated iMLL-AF9 cells. Violin plots show the distribution of log2 expression values (transcripts per million). (n = 4 RNA samples per group). **(e)** Relative levels of *BNIP3* mRNA (normalized to *β-ACTIN*) in MOLM13 cells transduced with lentiviruses expressing scrambled short hairpin RNA (*shCTL*) and a shRNA targeting *BNIP3* (*shBNIP3*) (n = 1 biological replicate per group, plated in triplicate). **(f)** Proliferation analyses of *shCTL-* and *shBNIP3-*transduced MOLM13 cells treated with IOX5 or vehicle control (n = 3 biological replicates per group). Data represent mean ± SEM. Comparisons not marked with a P value = ns. P values were calculated using a two-tailed Mann–Whitney U-test, paired or unpaired student *t*-test, unless stated otherwise. Source data

We next investigated the mechanism by which PHD inhibition compromises AML. The *iMLL-AF9* cells treated with IOX5 had dysregulated gene expression (Extended Data Fig. 7a) with significant upregulation of hypoxia and HIF-α signaling, as well as upregulation of HIF-mediated pathways, including glycolysis, pentose phosphate pathway, fructose and mannose metabolism, and apoptosis^11–13^ (Fig. 7a, left and Supplementary Table 2, top). Consistent with the anti-leukemic effect of IOX5, *iMLL-AF9* treated cells manifested downregulation of pathways, which normally promote, or are required for, oncogenic transformation (including MYC and E2F targets, tricarboxylic acid cycle, ribosome biogenesis, MTORC1 and RAN signaling)^43–49^ (Fig. 7a, left and Supplementary Table 2, bottom). Given that IOX5 inhibits PHD-mediated HIF-α degradation, we compared the dysregulated pathways in AML cells treated with IOX5 to those lacking HIF-α, that is *Hif1/2a*^DKO^ AML cells exposed to hypoxia^6^. We observed that multiple pathways upregulated in IOX5-treated cells were downregulated in *Hif1/2a*^DKO^ cells and vice versa (Fig. 7a and Supplementary Table 2). To explore the HIF-α dependency of the anti-leukemic effect of IOX5, we treated control and *Hif1/2a*^DKO^ cells with IOX5 and found that control AML cells manifest substantially reduced proliferation, whereas *Hif1/2a*^DKO^ cells are refractory to IOX5 and proliferate normally (Fig. 7b), indicating that IOX5 requires the intact HIF system to exhibit its anti-leukemic activity.

### PHD inhibition promotes HIF-dependent anti-leukemic program

Considering the HIF-α dependency of the anti-leukemic role of IOX5, we compared our transcriptomic analyses of IOX5-treated AML cells with available datasets of HIF-α dependent transcription^11–13^ and, as expected, multiple genes upregulated by IOX5 are positively regulated by HIF-α and vice versa (Extended Data Fig. 7b). Furthermore, we examined the intersection of up- and down-regulated transcripts in IOX5-treated cells with the down- and up-regulated transcripts in *Hif1/2a*^DKO^ cells, respectively, revealing HIF-dependent transcripts that are dysregulated by PHD inhibition (Fig. 7c). Examining transcripts that were downregulated upon IOX5 treatment (and upregulated upon loss of *Hif-α*) revealed a number of genes known to be overexpressed or to have oncogenic roles in leukemia, including *Hspa8*, *Nup98*, *Kpnb1* and *Rbm15* (refs. ^50–53^) (Extended Data Fig. 7c). Inspecting transcripts which are upregulated upon IOX5 treatment (and downregulated upon loss of *Hif-α*) we found increased expression of HIF target genes, including the 2OG-dependent JmjC histone demethylase *Kdm5b*, a known tumor suppressor in AML^33^. Notably, our analyses also revealed significant upregulation of the pro-apoptotic factor *Bnip3* (Extended Data Fig. 7d). Spearman rank analyses of transcripts upregulated upon IOX5 treatment and downregulated upon loss of *Hif-α* revealed a significant correlation and identified *Bnip3* as being inversely correlated with loss of *Hif-α* (Fig. 7d). Of note, PHD inhibition by both Dap and IOX5 strongly induced *BNIP3* expression in diverse established human AML cells (Fig. 7e). Furthermore, IOX5 also strongly upregulated BNIP3 protein in THP-1 cells (Fig. 7f) and *BNIP3* knockdown decreased the pro-apoptotic effect of IOX5 (Fig. 7g and Extended Data Fig. 7e,f). Therefore, the anti-leukemic effect of PHD inhibition is HIF-dependent. Thus, although the effects of HIF upregulation can be pleiotropic and context dependent^36^, our evidence implies the anti-leukemic effect of PHD inhibition is mediated, at least in part, through increased expression of *Bnip3*.

### PHD and BCL-2 co-inhibition efficiently ablates AML cells

**Fig. 8:**
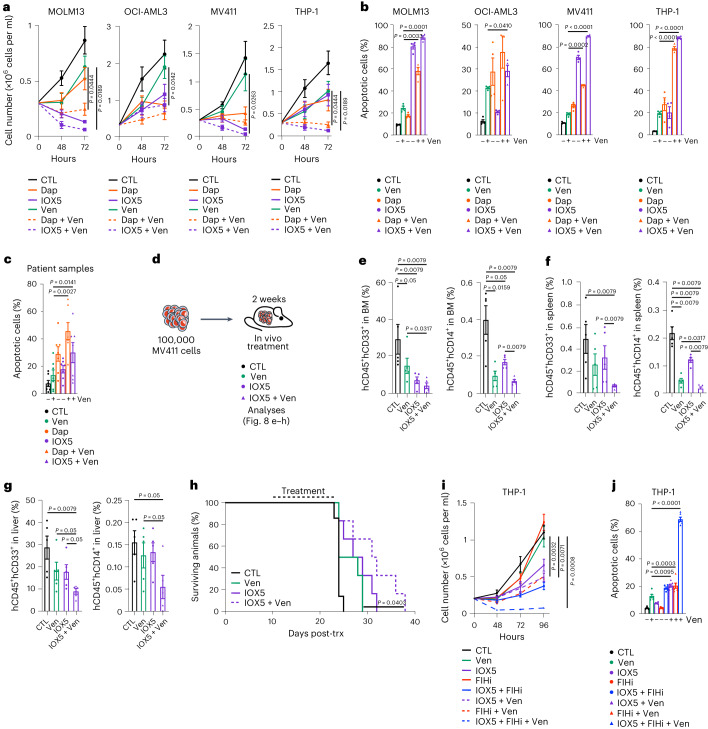
PHD inhibition combined with venetoclax ablates AML growth in vitro and in vivo*.* **a**,**b**, Proliferation and annexin V^+^PI^+^ analyses, respectively, of MOLM13, OCI-AML3, MV411 and THP-1 cells treated with Dap, IOX5, venetoclax (Ven), Dap + Ven, IOX5 + Ven or vehicle CTL (*n* = 4 biological replicates per group). Statistical significance between Ven only and Dap + Ven or IOX5 + Ven is represented on the graph. The statistical significance between CTL and all other experimental conditions is described below. MOLM13 *P* values; CTL versus IOX5 *P* = 0.0397, CTL versus Dap + Ven *P* = 0.0074, CTL versus IOX5 + Ven *P* = 0.0104. OCI-AML3 *P* values; CTL versus Dap *P* = 0.0325, CTL versus IOX5 *P* = 0.0047, CTL versus Dap + Ven *P* = 0.0143. MV411 *P* values; CTL versus Dap *P* = 0.0446, CTL versus IOX5 *P* = 0.0178, CTL versus Dap + Ven *P* = 0.0183, CTL versus IOX5 + Ven *P* = 0.0178. THP-1 *P* values; CTL versus IOX5 *P* = 0.0397, CTL versus Dap + Ven *P* = 0.0074, CTL versus IOX5 + Ven *P* = 0.0104. *P* values calculated at the 72-h time point (**a**). MOLM13 *P* values; CTL versus Dap *P* = 0.0049, CTL versus IOX5 *P* = 0.0001, CTL versus Ven *P* = 0.0033, CTL versus Dap + Ven *P* = 0.0005, CTL versus IOX5 + Ven *P* < 0.0001. OCI-AML3 *P* values; CTL versus Dap *P* = 0.0327, CTL versus IOX5 *P* = 0.0065, CTL versus Ven *P* = 0.0004, CTL versus Dap + Ven *P* = 0.0228, CTL versus IOX5 + Ven *P* = 0.0040. MV411 *P* values; CTL versus Dap *P* = 0.0028, CTL versus IOX5 *P* < 0.0001, CTL versus Ven *P* = 0.0029, CTL versus Dap + Ven *P* = < 0.0001, CTL versus IOX5 + Ven *P* = < 0.0001. THP-1 *P* values; CTL versus Dap *P* = 0.0236, CTL versus IOX5 *P* = 0.0484, CTL versus Ven *P* = 0.0008, CTL versus Dap + Ven *P* < 0.0001, CTL versus IOX5 + Ven *P* < 0.0001 (**b**). **c**, Percentage of annexin V^+^PI^+^ cells of individual patient samples treated with Dap, IOX5, Ven, Dap + Ven, IOX5 + Ven or vehicle CTL (*n* = 7 patients). Statistical significance represented as in **a**,**b**. *P* values; CTL versus Dap *P* = 0.0086, CTL versus IOX5 *P* = 0.0028, CTL versus Dap + Ven *P* = 0.0011, CTL versus IOX5 + Ven *P* = 0.0111. **d**, 100,000 MV411 cells were transplanted into NBSGW recipient mice. At 14 days following transplantation, recipient mice were treated with IOX5, Ven or vehicle CTL. Dosing regimen consisted of 2× daily via i.p. injection (IOX5 or vehicle) and/or 1× daily via o.g. (Ven or vehicle). After 14 days treatment, half of the cohort were analyzed for human AML cell engraftment, while the other half were observed for survival analyses. **e**, Percentage of human CD45^+^CD33^+^ and CD45^+^CD14^+^ cells, respectively, in BM (*n* = 5 mice per group). **f**, Percentage of human CD45^+^CD33^+^ and CD45^+^CD14^+^ cells, respectively, in the spleen (*n* = 5 mice per group). **g**, Percentage of human CD45^+^CD33^+^ and CD45^+^CD14^+^ cells, respectively, in the liver (*n* = 5 mice per group). **h**, Survival curve of mice treated with IOX5, Ven, IOX5 + Ven or vehicle CTL (CTL *n* = 7 mice; Ven, IOX5 and IOX5 + Ven *n* = 6 mice each). Statistical significance between Ven only and IOX5 + Ven is represented on the graph. Statistical significance between CTL and all other experimental conditions are described below. *P* values; CTL versus Ven *P* = 0.0403, CTL versus IOX5 *P* = 0.0059, CTL versus IOX5 + Ven *P* = 0.0027. **i**,**j**, Proliferation and annexin V^+^DAPI^+^ analyses, respectively, of THP-1 cells treated with Ven, IOX5, FIH inhibitor (FIHi; DM-NOFD), IOX5 + FIHi, IOX5 + Ven, FIHi + Ven, IOX5 + FIHi + Ven or vehicle CTL. Statistical significance between Ven only, IOX5 + Ven, FIHi + Ven or IOX5 + FIHi + Ven is represented on the graph. Statistical significance between CTL and all other experimental conditions are described below. CTL, Ven, FIHi, IOX5 + Ven, FIHi + Ven, IOX5 + FIHi + Ven *n* = 6; IOX5 *n* = 6 biological replicates per group at the 0, 24, 48 and 72-h time points, *n* = 5 biological replicates per group at the 96-h time point, IOX5 + FIHi *n* = 6 biological replicates per group at the 0, 24, 48 and 72-h time points, *n* = 5 biological replicates per group at the 96-h time point*. P* values; CTL versus IOX5 *P* = 0.0015, CTL versus IOX5 + FIHi *P* = 0.0054, CTL versus IOX5 + Ven *P* = 0.0004, CTL versus FIHi + Ven *P* = 0.0011, CTL versus IOX5 + FIHi + Ven *P* < 0.0001. IOX5 versus IOX5 + Ven *P* = 0.0091, IOX5 versus IOX5 + FIHi + Ven *P* = 0.0015. FIHi versus FIHi + Ven *P* = 0.0022, FIHi versus IOX5 + FIHi + Ven *P* < 0.0001. *P* values calculated at the 96-h time point (**i**). *n* = 6 biological replicates per group. *P* values; CTL versus Ven *P* = 0.0003, CTL versus IOX5 *P* < 0.0001, CTL versus IOX5 + FIHi *P* < 0.0001, CTL versus IOX5 + Ven *P* < 0.0001, CTL versus FIHi + Ven *P* = 0.0002, CTL versus IOX5 + FIHi + Ven *P* < 0.0001 (**j**). IOX5 versus IOX5 + Ven *P* < 0.0001, IOX5 versus IOX5 + FIHi + Ven *P* < 0.0001. FIHi versus FIHi + Ven *P* = 0.0003, FIHi versus IOX5 + FIHi + Ven *P* < 0.0001. Data represent mean ± s.e.m. Comparisons with no *P* value are NS. *P* values were calculated using a two-tailed Mann–Whitney *U*-test, paired or unpaired Student’s *t*-test, unless stated otherwise. Kaplan–Meier survival curve statistics were determined using the log-rank (Mantel–Cox) test. Source data

Considering that PHD inhibition promotes AML apoptosis and increases expression of the HIF-dependent, pro-apoptotic BH3-family member BNIP3, we explored the anti-leukemic potential of further disruption of the BH3-driven apoptotic pathway. We combined PHD inhibition with the BCL-2 inhibitor venetoclax, which in combination with hypomethylating agents displays promising clinical utility in AML treatment^24^. The PHD inhibitor and venetoclax combination resulted in profound loss of proliferative potential along with an increase in apoptosis in human AML cells (Fig. 8a,b). Testing the PHD inhibitor/venetoclax combination against primary patient samples showed a marked advantage of this therapy over single agent PHD inhibitor or venetoclax treatment (Fig. 8c and Supplementary Table 1).

Finally, we investigated the efficacy of the IOX5/venetoclax combination in vivo (Fig. 8d). We engrafted MV411 AML cells (with *MLL-AF4* and *FLT3-ITD* mutation) and found that IOX5 or venetoclax treatment alone decreased leukemic burden and extended survival of the recipient mice (Fig. 8e–h). Moreover, the combination therapy further compromised leukemic burden and further extended mouse survival, compared to IOX5 or venetoclax alone (Fig. 8e–h). Thus, dual PHD and BCL-2 inhibition act in concert to efficiently promote AML cell apoptosis, decrease AML burden and prolong mouse survival, revealing a promising therapeutic strategy in AML treatment.

### Combined PHD and FIH inhibition to eliminate AML cells

**Extended Data Fig. 8:**
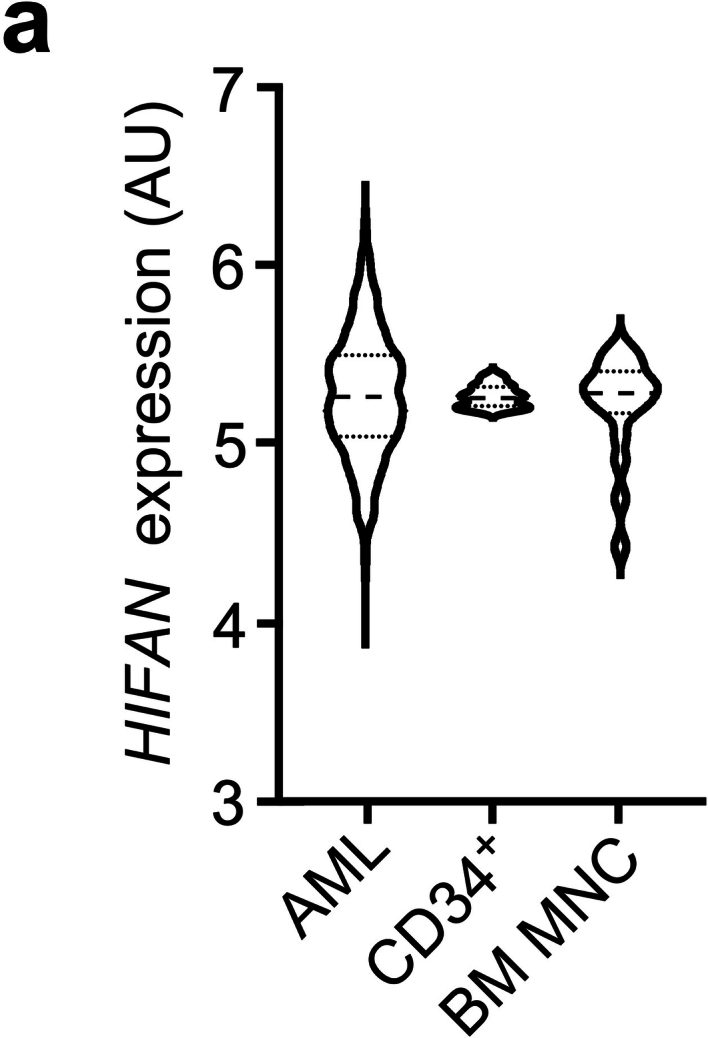
FIH expression across healthy and AML samples. **(a)** *FIH* (*HIFAN*) gene expression, respectively, across human AML bone marrow samples, healthy CD34^+^ BM cells and BM MNCs. (CD34^+^ n = 16 patients; BM MNC n = 19 patients; AML n = 671 patients). Source data

In addition to the post-transcriptional regulation of protein degradation by the PHD-VHL system^9^, HIF-α activity is regulated by FIH, which hydroxylates asparaginyl HIF-α residues to restrain HIF’s transcriptional activity^7^. FIH inhibition increases the affinity of HIF-α to transcriptional co-activators p300/CBP, thus promoting HIF-mediated transcription. We found that FIH (*HIFAN*) is comparably expressed in both human AML cells and primitive and mature blood cells from healthy donors (Extended Data Fig. 8a). Given our previous findings that both PHD and FIH inactivation may be needed to achieve optimal HIF activity^36^, we investigated the impact of dual PHD/FIH inhibition on AML cells. The FIH inhibitor, *N*-oxalyl-D-phenylalanine (NOFD), selectively inhibits FIH over many other 2OG oxygenases and does not inhibit PHD2 (Fig. 4e)^54^. We found that a prodrug form of an FIH inhibitor DM-NOFD^36,55^ alone had no impact on proliferation and survival of AML cells (Fig. 8i,j). Notably, however, the combined treatment of AML cells with IOX5 and DM-NOFD had a more potent anti-leukemic effect compared to IOX5 treatment alone (Fig. 8i,j). Given that venetoclax potentiated the anti-leukemic activity of IOX5, we investigated the effect of the triple combination of PHD and FIH inhibitors and venetoclax in AML. This combination severely compromised AML cells compared to single or double combinations used (Fig. 8i,j). Therefore, both increased HIF-α stability (by PHD inhibition) and enhanced HIF-α transcriptional activity (by FIH inhibition) are desirable to elicit a strong therapeutic effect against AML, which can be further potentiated by venetoclax.

## Discussion

The identification of AML-specific and nontoxic therapies is a major challenge in hemato-oncology. Genetic HIF-1 and/or HIF-2 deletion accelerates leukemogenesis^5,6^, suggesting a tumor-suppressor function of HIFs in AML; however, the functional significance of activation of the HIF system on AML has not previously been explored. We found that genetic inactivation of two suppressors of the HIF pathway, PHD1 and PHD2, attenuates AML. Concordantly, pharmacological PHD inhibition compromises AML cells in a HIF-dependent manner, induces AML cell apoptosis, reduces AML burden and impairs disease progression. Notably, inhibition of PHDs has no detrimental consequences on normal hematopoiesis, implying a broad therapeutic window for deploying PHD inhibitors in AML. Indeed, PHD inhibition promotes HSC quiescence, facilitates hematopoietic regeneration following injury, enhances erythropoiesis and protects organs from chemotherapy-mediated damage in a HIF-dependent manner^56,57^. These results highlight the therapeutic potential of PHD targeting in AML and imply that PHD inhibition may compromise AML while counteracting AML-induced suppression of normal hematopoiesis and the negative impact of chemotherapy. On the other hand, PHD inhibition by Dap impairs murine B cell development^42^, while DMOG, a broad spectrum inhibitor of 2OG-dependent oxygenases, compromises human monocyte survival^23^. Thus, while PHD targeting has a considerable therapeutic potential in AML, the role of the immune response during PHD inhibition merits further investigations.

Given the availability of relatively nontoxic clinically used PHD inhibitors, our findings are of considerable translational potential for AML treatment. Clinical trials testing Dap and molidustat in renal anemia have shown that chronic administration of these compounds is generally well tolerated, does not cause severe adverse effects and promotes erythropoiesis^8^. While these compounds are partially selective for PHD inhibition and may well be suitable for AML treatment, they inhibit other 2OG-dependent oxygenases, including JMJD6 (refs. ^7,15^), which is required for normal hematopoiesis and HSC maintenance^58^. Given that normal hematopoiesis is suppressed by AML, it is undesirable to inhibit key regulators of hematopoiesis while targeting the disease. Thus, we employed a structure-guided approach to generate a potent and selective PHD inhibitor, with particular emphasis on selectivity with respect to other 2OG-dependent oxygenase family members. We targeted a PHD-specific pocket located where HIF-α binds, and which is potentially involved in substrate binding^59^. Indeed, knowing that the PHDs are highly selective for HIF-α^10^, we hypothesized that designing a compound that both binds to the PHD-specific pocket, as well as active site Fe(II), might improve inhibition selectivity, as indeed was the case for IOX5.

IOX5 displays potent anti-leukemic activity comparable to Dap, with both compounds increasing HIF-α levels and activating HIF-dependent transcriptional networks. In addition to suppressing numerous genes which promote oncogenic transformation, IOX5 promotes the expression of a HIF-1 target *Bnip3*, a pro-apoptotic member of the BH3 family^60^. One of the multifaceted roles of BNIP3 is to activate apoptosis through localization to the mitochondria and activation of BAX/BAK, which permeabilize the outer mitochondrial membrane and destabilize mitochondrial membrane potential, leading to subsequent cell death^61,62^. BAX/BAK are restrained by the anti-apoptotic BCL-2, and BCL-2 inhibition by venetoclax, which consequently promotes BAX/BAK-mediated apoptosis, has already advanced AML treatments^24^. With this in mind, we combined IOX5 and venetoclax with the aim of enabling BAX/BAK-dependent apoptosis and discovered that BCL-2 inhibition potentiates the pro-apoptotic effect of PHD inactivation. Therefore, we propose that the effects of IOX5 and venetoclax converge to activate AML cell death, thus highlighting an attractive therapeutic strategy.

The significance of the HIF system in AML has been unclear. While HIFs were initially proposed to promote AML^3,4^, subsequent genetic evidence indicated that they are tumor suppressors^5,6^. Our results clearly demonstrate that activation of the HIF system is insufficient to induce spontaneous leukemogenesis. Furthermore, they imply that HIFs fail to accelerate oncogene-driven AML. On the contrary, increasing HIF stability through PHD targeting strongly suppresses AML, thus highlighting the anti-leukemic role of HIFs in AML.

Our results indicate that PHD inhibition compromises AML driven by diverse genetic alterations. We found that PHD inactivation has a significant anti-leukemic effect in AML driven by expression of *Meis1*/*Hoxa9*, both of which are frequently overexpressed in human AML^25–27^. We also demonstrated that PHD inhibition activates apoptosis in AML cells harboring diverse genetic alterations, including *MLL-AF9*, which function upstream of *Meis1* and *Hoxa9*, as well as *AML-ETO*, *PML-RARα* and *FLT3-ITD* (with and without *NPM1* mutations). Dap and IOX5 promote death in cells from primary AML samples from patients with poor-risk AML harboring complex karyotype with or without *TP53* mutations. Therefore, while our data suggest a broad applicability of PHD inhibitors in AML, further investigations are warranted to identify those AML genomic subgroups that are particularly sensitive to PHD inhibition, singly or in combination with FIH inhibition and venetoclax.

Together, our preclinical investigations provide proof-of-concept evidence that selective PHD inhibition is highly anti-leukemogenic, setting the stage for a promising therapeutic approach to AML treatment, without any toxic effects on normal hematopoiesis. HIF upregulation may be achieved by employing either the existing clinically used PHD inhibitors or highly selective PHD inhibitors developed specifically for AML treatment, such as IOX5.

## Methods

### Inclusion and ethics statement

This study has been a collaborative project between the Institute of Cancer Research, Barts Cancer Institute and the University of Oxford, with other significant contributions made across the UK and Europe. All experiments were conducted following extensive training and, where appropriate, following approval by ethical bodies.

### Mice

All experiments on animals were performed under UK Home Office authorization under the project license PP4153210 at Barts Cancer Institute following approval by Queen Mary University of London AWERB. Animals were subject to an optimum dark–light cycle, with ambient temperature and humidity. All mice were on the C57BL/6 genetic background. *Phd2*^fl/fl^, *shPhd2* and *iMLL-AF9* mice were described previously^28–30^. *Vav-iCre* and NBSGW mice were purchased from The Jackson Laboratory^63,64^. All transgenic and knockout mice were CD45.2^+^. Congenic recipient mice were CD45.1^+^/CD45.2^+^. Mice used for support BM cells during transplantation experiments were CD45.1^+^. Unless stated otherwise, all in vivo experiments had mixed-sex animals aged between 8–12 weeks when experiments began. All animals were monitored twice daily and humanely killed at the experimental end point, which was not exceeded during this study. For animals analyzed in steady-state hematopoiesis, HSC or BM transplantation, or drug toxicity studies, the end point is as described in the experimental design stated in the figure legends for each experiment. For animals employed in AML studies, the humane end point was determined either by the experimental design or in the case of survival studies, by disease progression. Disease progression was measured against a clinical scoring system detailed in the UK Home Office project license. The parameters studied include animal weight, appearance, body condition, clinical signs and natural and provoked behavior.

### Human tissue and ethical approvals

All use of human tissue was in compliance with the ethical and legal framework of the UK’s Human Tissue Act, 2004. Primary human AML samples were from Barts Cancer Institute Biobank (with approval of the Research Ethics Committee). Their use was authorized following ethical review by the Tissue Biobank’s scientific sub-committee and with the informed consent of the donors. For all samples used in this study, Barts Cancer Institute Biobank obtained informed consent from all participants.

### Flow cytometry

All BM, fetal liver (FL) and splenic cells were prepared and analyzed as described^6,47,58,65–68^. BM cells were isolated by crushing tibias and femurs using a pestle and mortar. FL cells were prepared by mashing the tissue and passing through a 70-µm strainer. Single cell suspensions from BM, FL or PB were incubated with Fc block and then stained with antibodies. For HSC and progenitor cell analyses, following incubation with Fc block, unfractionated BM cells were stained with lineage markers containing biotin-conjugated anti-CD4, anti-CD5, anti-CD8a, anti-CD11b, anti-B220, anti-Gr-1 and anti-Ter119 antibodies together with BV711-conjugated anti-c-Kit, APC-Cy7-conjugated anti-Sca-1, PE-conjugated anti-CD48 and PE-Cy7-conjugated anti-CD150 antibodies. Biotin-conjugated antibodies were then stained with PB-conjugated streptavidin. For analyses of differentiated cells, following incubation with Fc block, spleen or BM cell suspensions were stained with PerCP-conjugated anti-B220 and APC-Cy7-conjugated anti-CD19 antibodies for B cells; APC-conjugated anti-CD11b and PE-Cy7-conjugated anti-Gr-1 for myeloid cells; PE-conjugated anti-CD4 and anti-CD8 antibodies for T cells.

To distinguish CD45.2^+^-donor-derived cells in PB or BM of transplanted mice, BV711-conjugated anti-CD45.1 and Pacific Blue-conjugated anti-CD45.2 antibodies were used. For HSC and progenitor staining in transplanted mice, APC-conjugated anti-c-Kit and PerCP-conjugated streptavidin was used; the remainder of the staining was as described above. For analyses of differentiated cells in PB and BM of transplanted mice, myeloid cells and lymphoid cells were stained as described above. TO-PRO-3 or 4,6-diamidino-2-phenylindole (DAPI) were used for dead cell exclusion.

To assess human AML burden, cells were stained with anti-human FITC-conjugated anti-CD45, APC-conjugated anti-CD33 and anti-human PE-conjugated CD14.

Flow cytometry analyses were performed using a LSRFortessa (BD). Cell sorting was performed on a FACSAria Fusion (BD). Representative flow cytometry gating strategies are available in Supplementary Figs. 10–17.

### Leukemic transformation

Leukemic *Meis1*/*Hoxa9, FLT3-ITD* and *PML-RARα* cells were prepared as described^68,69^. Transduced cells were subjected to three rounds of colony-forming cell (CFC) assays in MethoCult M3231 (STEMCELL Technologies) supplemented with 20 ng ml^−1^ SCF, 10 ng ml^−1^ IL-3, 10 ng ml^−1^ IL-6 and 10 ng ml^−1^ granulocyte–macrophage colony-stimulating factor. Colonies were counted 5–7 days after plating and 2,500 cells were re-plated.

### Syngeneic transplantation assays

CD45.1^+^/CD45.2^+^ recipient mice were lethally irradiated using a split dose of either; a 11 Gy (two doses of 5.5 Gy administered at least 4 h apart) at an average rate of 0.58 Gy min^−1^ using a Cesium 137 GammaCell 40 irradiator or 8 Gy (two doses of 4 Gy administered at least 4 h apart) at an average rate of 1.086 Gy min^−1^ using a RADSOURCE X-ray irradiator.

For primary transplantations of leukemic cells, 100,000 *Meis1*/*Hoxa9*-transduced c-Kit^+^ cells or 2,000 *iMLL-AF9* LSK cells were transplanted into lethally irradiated CD45.1^+^/CD45.2^+^ recipient mice (together with 200,000 unfractionated support CD45.1^+^ wild-type BM cells). For secondary transplantations of leukemic cells, 50,000 cells collected from primary recipients were transplanted into lethally irradiated CD45.1^+^/CD45.2^+^ recipient mice (together with 200,000 unfractionated support CD45.1^+^ wild-type BM cells). Recipients were culled upon reaching their humane end point as recorded in survival curves.

For LDA analyses, increasing doses (10,000, 50,000 and 100,000) of CD45.2^+^ BM cells from primary transplantation were re-transplanted into lethally irradiated CD45.1^+^/CD45.2^+^ recipient mice (together with 200,000 unfractionated support CD45.1^+^ wild-type BM cells). LSC frequency was calculated using ELDA software^70^.

For primary transplantations of healthy total BM cells, 5,00,000 total BM cells were mixed with 5,00,000 support CD45.1^+^ BM cells. For primary transplantations of HSCs, 200 LSKCD48^-^CD150^+^ HSCs (per recipient) sorted from the BM of donor mice were mixed with 200,000 support CD45.1^+^ BM cells and transferred into lethally irradiated CD45.1^+^/CD45.2^+^ recipients. All recipient mice were killed and analyzed 16–20 weeks post-transplantation.

For animals treated with DOX, mice were provided ad libitum access to drinking water containing 2 mg ml^−1^ DOX with 30% sucrose.

### In vivo treatment with PHD inhibitors

For AML experiments, 100,000 of THP-1, OCI-AML3 or MV411 cells were injected via tail vein into non-irradiated 10–12-week-old mixed-sex NBSGW mice and began drug treatment 14 days after transplantation. Mice were injected intraperitoneally (i.p.) twice daily for 21 days with 30 mg kg^−1^ Dap (an optimal in vivo concentration^14,42^), 30 mg kg^−1^ IOX5 or vehicle control. Mice in combination treatment experiments with venetoclax (ABT-199) (MCE) were dosed once daily with 100 mg kg^−1^ via oral gavage (o.g.).

For steady-state analyses, 8–10-week-old mixed-sex C57BL6 mice were injected i.p. twice daily for 14 days with 30 mg kg^−1^ IOX5 or vehicle control. Mice were bled before and after treatment and killed 12 h after the final dosing.

### Cell proliferation and cell death analyses

Cells were cultured with 50 μM Dap (an optimal concentration in vitro^71,72^), 50 μM IOX5, 50 μM molidustat, 50 μM roxadustat (an optimal concentration in vitro^73^), 100 μM DM-NOFD, 0.01 μM venetoclax (MV411 and MOLM13), 0.1 μM venetoclax (THP-1 and OCI-AML3) or vehicle control. Viable cells were counted by Trypan blue exclusion at the indicated time points. To analyze cells undergoing apoptosis, cells were suspended in binding buffer containing PE-conjugated annexin V or FITC-conjugated annexin V and either PI or DAPI.

### Primary human AML patient-derived samples

All patients involved in this study gave informed consent for storage and use of their tissue for research purposes. The study was approved by the Institutional Review Board of Barts Cancer Institute and all work was performed in accordance with the Declaration of Helsinki and the Local Research Ethics Committee requirements. Frozen AML samples were obtained from the Barts Cancer Institute Biobank and quickly thawed at 37 °C. Upon thawing, T cells were depleted from all samples using EasySep Human TCR Alpha/Beta Depletion kit (STEMCELL Technologies, 17847). Enriched samples were plated with concentrations 0.4–1.0 × 10^6^ ml^−1^ in Myelocult H5100 medium (STEMCELL Technologies, 05150) supplemented with 20 ng ml^−1^ IL-3, granulocyte–macrophage colony-stimulating factor and TPO (BioLegend) in co-culture with irradiated MS-5 cells and treated with 50 μM Dap, 50 μM IOX5, 1 μM venetoclax or vehicle control for 7 days. After 3 days of treatment, cells were supplemented with fresh medium containing the corresponding agent in volume equivalent to 50% of initial volume. After 7 days, viable cell numbers were counted and viability was assessed using annexin V FITC/PI stain.

### Synthesis of PHD inhibitors

Roxadustat (FG-4592) was from Cayman Chemical. Molidustat (BAY 85-3934) was from Selleckchem. Dap was synthesized following a reported procedure as described previously^15^.

#### 1,3-Dicyclohexylpyrimidine-2,4,6(1*H*,3*H*,5*H*)trione (1a)

A suspension of *N*,*N*′-dicyclohexylcarbodiimide (3.5 g, 17.00 mmol) in THF (13 ml) was slowly added to a solution of malonic acid (884.5 mg, 8.50 mmol) in THF (13 ml) at 0 °C. After allowing the reaction mixture to warm up room temperature, it was stirred for 2 h. The resulting suspension was filtered and the solvent was removed in vacuo. Re-crystallization from ethanol gave a white fibrous solid (1.625 g, 5.56 mmol, 65%).

The melting point (m.p.) is 201.3–202.4 °C. ^1^H nuclear magnetic resonance (NMR) (600 MHz, DMSO-d_6_): δ (ppm) = 4.45 (tt, *J* = 12.2 Hz, 3.7 Hz, 2H,), 3.68 (s, 2H), 2.13 (qd, *J* = 12.6 Hz, 3.6 Hz, 4H), 1.76 (m, 4H), 1.58 (td, *J* = 16.2 Hz, 7.8 Hz, 6H), 1.25 (qt, *J* = 13.3 Hz, 3.6 Hz Hz, 4H), 1.08 (m, 2H).^13^C NMR (151 MHz, DMSO-d_6_): δ (ppm) = 166.0, 151.5, 53.7, 41.2, 28.7, 26.0 and 25.1. IR (ATR): $\widetilde{{\rm{v}}}$ (cm^−1^) = 2,972, 2,930, 2,890, 1,695, 1,676, 1,412, 1,387, 1,330 and 1,204. ESI-HRMS (*m*/*z*): [M − H]^−^ calculated for [C_16_H_23_N_2_O_3_]−: 291.1714, found: 291.1710. The analytical data are consistent with those previously reported^15^.

#### Ethyl (1,3-dicyclohexyl-6-hydroxy-2,4-dioxo-1,2,3,4-tetrahydropyrimidine-5-carbonyl)glycinate (1b)

**1a** (1.625 g, 5.56 mmol) and *N*,*N*-diisopropylethylamine (DIPEA; 1.94 ml, 11.12 mmol) were dissolved in CH_2_Cl_2_ (30 ml). Ethyl isocyanatoacetate (0.62 ml, 5.56 mmol) was added dropwise; the solution was then stirred at room temperature for 22 h. The reaction mixture was diluted with CH_2_Cl_2_ (15 ml), extracted with 6 M HCl (5 ml), then dried over sodium sulfate. After removing the solvent in vacuo, the solid was re-crystallized from cyclohexane, giving a white powder (2.303 g, 5.46 mmol, 98.3%).

The m.p. is 159.0–159.7 °C. ^1^H NMR, (600 MHz, CDCl_3_): *δ* (ppm) = 10.35 (s, 1H), 4.71 (m, 2H), 4.26 (q, *J* = 7.1 Hz, 2H), 4.15 (d, *J* = 5.7 Hz, 2H), 2.34 (tq, *J* = 15.5 Hz, 6.2 Hz, 4H), 1.83 (t, *J* = 11.4 Hz, 4H), 1.64 (m, 6H), 1.30 (m, 9H).^13^C NMR (151 MHz, CDCl_3_): *δ* (ppm) = 171.9, 169.3, 168.5, 163.5, 149.8, 81.0, 62.0, 41.6, 29.4, 29.1, 26.6, 26.6, 25.5, 25.4 and 14.3. IR (ATR): $\widetilde{v}$ (cm^−1^) = 2,981, 2,887, 1,751, 1,384, 1,252, 1,148 and 1,074. ESI-HRMS (*m*/*z*): [M + H]^+^ calculated for [C_21_H_31_N_3_O_6_]^+^: 422.2286, found: 422.2283. The analytical data are consistent with those previously reported in Yeh et al.^15^.

#### (1,3-Dicyclohexyl-6-hydroxy-2,4-dioxo-1,2,3,4-tetrahydropyrimidine-5-carbonyl)glycine (Dap)

**1b** (2.3 g, 5.46 mmol) was suspended in ethanol (40 ml), then 4 M sodium hydroxide solution (5 ml) was added slowly. After 2 h stirring at room temperature, 2 M HCl (12 ml) was added. The resulting precipitate was filtered and was stirred in water (50 ml) at 35 °C for 1 h and again filtered to give Dap (1.504 g, 3.82 mmol, 70.0%) as a white solid.

The m.p. is 221.6–223.0 °C. ^1^H NMR (600 MHz, DMSO-*d*_6_): *δ* (ppm) = 10.15 (t, *J* = 5.7 Hz, 1H), 4.63 (tt, *J* = 12.3 Hz, 3.7 Hz, 2H), 4.03 (d, *J* = 5.4 Hz, 2H), 2.28 (qd, *J* = 12.5 Hz, 3.6 Hz, 4H), 1.77 (dt, *J* = 13.2, 3.4 Hz, 5H), 1.64–1.50 (m, 6H), 1.26 (qt, *J* = 13.2 Hz, 3.6 Hz, 4H), 1.11 (qt, *J* = 13.0 Hz, 3.3 Hz, 2H). ^13^C NMR (151 MHz, DMSO-*d*_6_): *δ* (ppm) = 170.2, 170.1, 149.4 53.0, 41.5, 28.7, 26.1, 25.1. IR (ATR): $\widetilde{v}$ (cm^−1^) = 2,981, 2,933, 2,855, 2,665, 1,719, 1,663, 1,589, 1,489, 1,456 and 1,244. ESI-HRMS (*m*/*z*): [M + H]^+^ calculated for [C_19_H_28_N_3_O_6_]^+^: 394.1973, found: 394.1971. The analytical data are consistent with those previously reported in Yeh et al.^15^.

^1^H and ^13^C NMR of Dap are given in Supplementary Figs. 3 and 4, respectively.

### Synthesis of IOX5

#### 6-Chloro-4-methoxy-*N*-{*trans*-[4 -(trifluoromethyl)cyclohexyl]methyl}nicotinamide (2a)

6-Chloro-4-methoxy-nicotinic acid (250 mg, 1.33 mmol), C-(4-trifluoromethyl)-cyclohexylmethylamine (Fluorochem) (255 mg, 1.59 mmol), propylphosphonic anhydride (T3P, 827 mg, 2.6 mmol) were dissolved in dimethylformamide (DMF; 10 ml); DIPEA (803 µl, 4.67 mmol) was then added. The resultant mixture was stirred overnight at room temperature. EtOAc (20 ml) and H_2_O (100 ml) were added to the reaction mixture. The organic and aqueous fractions were then separated, and the aqueous layer was extracted with EtOAc (30 ml) twice. The combined organic fractions were washed with brine, then dried with anhydrous Na_2_SO_4_. The crude mixture purified using flash-column chromatography using cyclohexane (100 to 50%) and EtOAc (0 to 50%) over ten column volumes to give **2a** (402 mg, 1.14 mmol, 86%) as a white solid.

The m.p. is 67–70 °C. ^1^H NMR (600 MHz, CDCl_3_): δ 8.99 (s, 1H, H_2_), 7.41 (bs, 1H, H_10_), 6.92 (s, 1H, H_5_), 4.04 (s, 3H, H_20_), 3.33 (t, *J* = 6.1 Hz, 2H, H_12_), 2.01–1.88 (m, 5H, H_14_, H_15_, H_16_, H_17,_ H_18_), 1.62–1.55 (m, 1H, H_13_), 1.31 (qd, *J* = 13.0, 3.2 Hz, 2H, H_15′_, H_17′_), 1.07–0.99 (m, 2H, H_14′_, H_18′_).^13^C NMR (151 MHz, CDCl_3_): δ 164.38 (C9), 163.06 (C6), 155.51 (C4), 153.85 (C2), 130.53–125.00 (q, *J* = 278.4 Hz, C19), 116.99 (C1), 106.99 (C5), 56.91 (C20), 45.60 (C12), 41.85 (q, *J* = 26.5 Hz, C16), 37.45 (C13), 29.33 (C14, C18), 24.8 (C15, C17). FT-IR *V*_max_ (film): 3,411 and 1,652 cm^−1^. HRMS (ESI-TOF) calculated for C_15_H_19_O_2_N_2_^35^ClF_3_ [M + H] ^+^: 351.1081, found: 351.1078.

#### 4-Methoxy-6-(1*H*-pyrazol-1-yl)-*N*-{*trans*-[4-(trifluoromethyl)cyclohexyl]methyl} nicotinamide (2b)

**2a** (100 mg, 0.277 mmol), pyrazole (23 mg, 0.33 mmol), cesium carbonate (Cs_2_CO_3_,180 mg, 0.554 mmol), Pd^t^BuXPhos G3 ((2-di-*tert*-butylphosphino-2′,4′,6′-triisopropyl-1,1′-biphenyl)-2-(2′-amino-1-1′-biphenyl)) palladium (II) methanesulfonate) (19 mg, 0.027 mmol) were placed under N_2_, then anhydrous tert-butanol (^t^BuOH, 2 ml) was added. The resultant mixture was heated for 16 h at 60 °C. The reaction mixture was allowed to cool to room temperature, then filtered through a Celite pad. EtOAc (20 ml) and H_2_O (100 ml) were added to the mixture and the organic and aqueous fractions were separated. The aqueous layer was extracted twice with EtOAc (30 ml). The organic fractions were combined, washed with brine and dried with anhydrous Na_2_SO_4_. The crude mixture was purified using flash-column chromatography using (0% to 100% EtOAc, in cyclohexane) over 20 column volumes to give **2b** (49 mg, 0.127 mmol, 46 %).

^1^H NMR (400 MHz, CDCl_3_): δ 9.07 (s, 1H, H_2_), 8.62 (d, *J* = 2.6 Hz, 1H, H_20_), 7.75 (d, *J* = 1.6 Hz, 1H, H_22_), 7.60 (s, 1H, H_5_), 7.54 (bs, 1H, H_10_), 6.49 (dd, *J* = 2.7, 1.7 Hz, 1H, H_21_), 4.13 (s, 3H, H_23_), 3.35 (t, *J* = 6.4 Hz, 2H, H_12_), 2.06–1.90 (m, 5H, H_14_, H_15,_ H_16_, H_17_), 1.69–1.57 (m, 1H, H_13_), 1.43–1.20 (m, 2H, H_15′_, H_17′_), 1.05 (qd, *J* = 12.8, 2.6 Hz, 2H, H_14′_, H_18′_). ^13^C NMR (101 MHz, CDCl_3_): δ 164.93 (C9), 163.40 (C6), 154.60 (C4), 153.02 (C2), 142.57 (C22), 130.71–125.16 (q, *J* = 278.2 Hz, C24), 127.76 (C20), 115.25 (C1), 108.25 (C21), 94.38 (C5), 56.47 (C23), 45.22 (C12), 41.68 (q, *J* = 26.4 Hz, C16), 37.22 (C13), 29.08 (C14, C18), 24.34 (C15, C17). FT-IR *V*_max_ (film): 3,132, 1640, 1,550 cm^−1^. HRMS (ESI-TOF) calculated for C_18_H_22_O_2_N_4_F_3_ [M + H]^+^: 383.1689, found: 383.1689.

#### 4-Oxo-6-(1*H*-pyrazol-1-yl)-*N*-{*trans-*[4-(trifluoromethyl)cyclohexyl]methyl}-1,4-dihydropyridine-3-carboxamide (IOX5)

**2b** (30 mg, 0.078 mmol) and lithium chloride (33 mg, 0.78 mmol) were dissolved in *N*,*N*-dimethylacetamide (DMAc, 2 ml). The resultant mixture was heated with microwave irradiation at 120 °C for 8 h. The resultant mixture was diluted with H_2_O (100 ml) and extracted with EtOAc (3 × 20 ml). The combined organic phases were washed with water, brine and dried with anhydrous Na_2_SO_4_. The volatiles were then evaporated in vacuo and the crude mixture was purified by flash-column chromatography using (0% to 10% methanol in CH_2_Cl_2_) over 15 column volumes to give IOX5 (8.5 mg, 0.023 mmol, 30 %) as a white solid.

The m.p. is 176–181 °C. ^1^H NMR (600 MHz, DMSO): δ 9.11 (app. bs, 1H, H_10_), 8.71 (s, 1H, H_2_), 8.61 (d, *J* = 2.5 Hz, 1H, H_21_), 8.13 (s, 1H, H_3_), 7.84 (d, *J* = 1.5 Hz, 1H, H_23_), 7.28 (s, 1H, H_5_), 6.58 (dd, *J* = 2.6, 1.6 Hz, 1H, H_22_), 3.19 (t, *J* = 6.4 Hz, 2H, H_12_), 2.22–2.13 (m, 1H, H_16_), 1.90–1.79 (m, 4H, H_14_, H_15_, H_17_, H_18_), 1.61–1.49 (m, 2H, H_13_), 1.21 (qd, *J* = 12.9, 3.4 Hz, 2H, H_15′_, H_17′_), 1.03 (qd, *J* = 13.0, 3.4 Hz, 2H, H_14′_, H_18′_).^13^C NMR (151 MHz, DMSO): δ 165.98 (C9), 163.17 (C6), 152.12 (C4), 148.55 (C2), 142.86 (C23), 130.96–125.42 (q, *J* = 278.1 Hz, C19), 127.97 (C21), 113.64 (C1), 108.83 (C22), 99.76 (C5), 44.61 (C12), 40.85–40.35 (q, *J* = 25.4 Hz, C16), 36.80 (C13), 28.55 (C14, C18), 24.20 (C15, C17).^19^F NMR (565 MHz, DMSO): δ −70.96, −72.84 (d, *J* = 9.0 Hz). Low levels of unassigned peaks in the NMR spectra likely reflect conformational isomers or pyrimidine/pyridine/amide conformational isomers as precedented with related compounds (as evidenced by variations in the intensities of some of them in variable temperature NMR studies^74^. FT-IR *V*_max_ (film): 3,649, 3,442, 1,641, 1,574 cm^−1^. HRMS (ESI-TOF) calculated for C_17_H_20_O_2_N_4_F_3_ [M + H]^+^: 369.1532, found: 369.1533.

The synthetic route for IOX5 is shown Fig. 4f. NMR spectra are described in Supplementary Figs. 5–9, specifically ^1^H spectra for **2a** (Supplementary Fig. 5), ^1^H for **2b** (Supplementary Fig. 6), ^1^H for IOX5 (Supplementary Fig. 7), ^13^C for IOX5 (Supplementary Fig. 8) and ^19^F for IOX5 (Supplementary Fig. 9).

### Visualization of Dap and IOX5-binding mode models

The structures of Dap or IOX5 were drawn in Chemdraw v.19.1 and transferred to Chemdraw 3D. Minimized energy (MM2) calculations were applied to investigate the stable conformations of Dap or IOX5. The file was saved as a mol file and opened in the respective pdb model in PyMOL. Using the pair-fitting feature, the minimized structures of Dap or IOX5 were overlaid with the identified structures to give predicted binding modes of Dap or IOX5.

### Protein production

Recombinant truncated forms of PHD2 (ref. ^37^), FIH^75^, JMJD6 (ref. ^76^), KDM4A^77^, KDM5B^15,78^ and KDM6B^79^ were produced and purified from *Escherichia* *coli* as described. Full-length FIH (M1–N349), full-length OGFOD1 (M1–G542), full-length JMJD6 (M1–R423), PHD2 (181–426), KDM4A (M1-L359) and KDM6B (D1141–E1590) were produced and purified from *E.* *coli* as described. Summaries of the general procedures used are given here.

DNA encoding for human FIH (M1–N349) and KDM4A (M1–L359), with an N-terminal His6-tag were cloned into the pNIC28-Bsa4 vector. DNA encoding for human JMJD6 (M1–R423), OGFOD1 (M1–G542) and PHD2 (181–426) containing an N-terminal His6-tag were cloned into the pET-28b vector and that for KDM6B (D1141–E1590) into pNH-TrxT vector. The constructs were transformed into *E.* *coli* strain BL21(DE3).

A 10-ml overnight culture of each target was used to inoculate each of 12 l Terrific Broth medium containing 100 μg ml^−1^ kanamycin. Cultures were grown at 37 °C until the OD_600_ reached 1.0, cooled to 18 °C and induced for 18 h with 0.5 mM IPTG. Cells were collected, resuspended in lysis buffer, lysed with three passages through a high-pressure cell breaker and clarified by centrifugation at 21,000 rpm for 30 min. The lysis buffer contained 50 mM HEPES, pH 7.4, 500 mM NaCl, 20 mM imidazole, 0.5 mM Tris (2-carboxyethyl)-phosphine (TCEP) and 5% glycerol. JMJD6 (M1–R423) and OGFOD1 (M1–G542) contained a Tris-based lysis buffer (50 mM Tris-HCl (pH 8), 200 mM NaCl, 20 mM imidazole, 0.5 mM TCEP and 5% glycerol). Protease inhibitor mix 1:2,000 was added for lysis (Complete, EDTA-free Protease Inhibitor Cocktail, Roche Diagnostics).

The clarified supernatant was passed through an Ni NTA gravity column. After ten column volume washes with lysis buffer, the His_6_-tagged proteins were eluted with 300 mM imidazole. For JMJD6 (M1–R423), OGFOD1 (M1–G542) and PHD2 (181–426) the eluted fraction was incubated with 100 mM EDTA for 1 h at 4 °C. The eluted fractions containing appropriately purified proteins were further purified with a S200 Gel Filtration column equilibrated with 50 mM HEPES (pH 7.4), 150 mM NaCl, 0.5 mM TCEP and 5% glycerol. For JMJD6 (M1–R423) and OGFOD1 (M1–G542), a Tris-based buffer was used (Tris-HCl, pH 8, 200 mM NaCl, 0.5 mM TCEP and 5% glycerol). The monodispersed peak containing the respective protein was collected. As reported, the purities and molecular masses of the desired products were validated by SDS–PAGE and intact electrospray ionization mass spectrometry (PHD2 (ref. ^37^), FIH^75^, JMJD6 (ref. ^76^), KDM4A^77^, KDM5B^15,78^ and KDM6B^79^).

### KDM5B (M1–R822)

cDNA encoding for KDM5B (residues M1–R822) was cloned into the pFB-LIC vector, encoding for a protein with a TEV-protease cleavable N-terminal 6x-histidine tag via ligation independent cloning. Human KDM5B was then expressed in Sf9 (*Spodoptera* *frugiperda*) insect cells.

Exponentially growing Sf9 cells (2 × 10^6^ cells per ml) were infected with high titer baculovirus stock and incubated in shaker flasks. The cells were shaken at 90 rpm at 27 °C and collected 72 h after infection by centrifugation (15 min, 800*g*, 4 °C). The cell pellets were washed and resuspended in PBS. The cells were centrifuged again and the cell pellets were stored at −80 °C.

For protein purification, the cell pellet was thawed, resuspended in lysis buffer (50 mM HEPES, pH 7.4, 200 mM NaCl, 20 mM imidazole, 5% glycerol and 0.5 mM TCEP) in the presence of a protease inhibitor mix 1:2,000 (Complete, EDTA-free Protease Inhibitor Cocktail, Roche Diagnostics) and sonicated (2 min, amplitude 35%, on ice). The lysate was cleared by centrifugation (60 min, 36,000*g*, 4 °C). The protein was purified in the same methodology used for KDM4A (M1–L359) and KDM6B (D1141–E1590) production.

### Inhibition assays

#### PHD2 solid phase extraction mass spectrometry assay

Inhibition of PHD2 (181–426) was measured using a solid phase extraction (SPE) mass spectrometry (MS) assay as reported previously^35^. In brief, compounds were dry dispensed into 384-well polypropylene plates using an ECHO 550 acoustic dispenser (Labcyte) approximating to a threefold dilution series across an 11-point concentration range (100 μM to 0.0017 μM). PHD2 (0.3 μM in 50 mM Tris-Cl, pH 7.5, 50 mM NaCl) was then dispensed across the plate using a Thermo Multidrop dispenser equipped with a small-volume dispensing cassette (25 μl per well). Compound dilutions were pre-incubated with PHD2 for 15 min; the reaction was initiated by dispensing 25 μl of a substrate solution (200 μM L-AA, 20 μM FAS, 20 μM 2OG and 10 μM CODD peptide) in 50 mM Tris-Cl, pH 7.5, 50 mM NaCl. The reaction was progressed for 15 min, then quenched by dispensing 5 μl 10% (*v*/*v*) aqueous formic acid. The assay plates were transferred to an Agilent RapidFire RF365 and processed as described in the section below (RapidFire SPE-MS Procedure).

### FIH SPE-MS assays

Inhibition of FIH was measured using a SPE-MS assay and synthetic peptide substrate (HIF-1α788-822) and monitoring hydroxylation of the peptide product. In brief, compounds were dry dispensed into 384-well polypropylene plates using an ECHO 550 acoustic dispenser (Labcyte) approximating to a threefold dilution series across an 11-point concentration range (100 μM to 0.0017 μM). FIH (0.3 μM in 50 mM Tris-Cl, pH 7.5, 50 mM NaCl) was then dispensed (25 μl per well) across the assay plate using a Thermo Multidrop dispenser equipped with a small-volume dispensing cassette. Compound dilutions were pre-incubated with FIH for 15 min and then the reaction initiated by dispensing 25 μl of a substrate solution (200 μM L-AA, 20 μM FAS, 20 μM 2OG and 10 μM HIF-1α788-822) in 50 mM Tris-Cl, pH 7.5 and 50 mM NaCl. The enzyme reaction was allowed to progress for 15 min, then halted by dispensing 5 μl 10% (*v*/*v*) aqueous formic acid. Assay plates were transferred to an Agilent RapidFire RF365 and processed as described in the section below (RapidFire SPE-MS Procedure).

### JMJD6 SPE-MS assay

The inhibitory effect of NOG, 2,4-pyridine dicarboxylic acid (2,4-PDCA), NOFD, Dap, roxadustat and molidustat on activity of full-length JMJD6 was measured using a 40-mer peptide substrate of bromodomain-containing protein 4 (BRD4_511–550_)^80^. Titrations of compounds were prepared using an ECHO 550 acoustic dispenser (Labcyte). An 11-point and threefold dilution for each compound (100 μM to 0.0017 μM) was prepared and dry dispensed into 384-well polypropylene plates. Full-length JMJD6 (1.0 μM in 50 mM Tris-Cl, pH 7.5) was then dispensed (25 μl per well) across the plate using a multidrop dispenser equipped with a low-volume dispensing cassette (Thermo). Compound dilutions were pre-incubated with JMJD6 for 15 min and the enzyme reaction was initiated by 25 μl dispense of the substrate mixture in 50 mM Tris-HCl, pH 7.5 (200 μM l-ascorbate, 20 μM ferrous ammonium sulfate, 20 μM 2-oxoglutarate and 10 μM JMJD6 substrate BRD4_511–550_). Reactions were progressed for 15 min at room temperature, then halted by dispensing 10% (*v*/*v*) aqueous formic acid (5 μl). The final concentration of DMSO was 0.5% (*v*/*v*). Assay plates were transferred to an Agilent RapidFire RF365 and processed as described in the section below (RapidFire SPE-MS procedure).

### JMJD6 IOX5 IC_50_ determination

Inhibition of the catalytic activity of recombinant human JMJD6 was measured using an N-terminal peptide (RSKKRKKSKSRS) of RNA Binding Motif Protein 39 (RBM39 residues 31–42) and monitoring the appearance of the hydroxylated peptide product in 50 mM Tris-Cl, pH 7.5. Titrations of IOX5 for IC_50_ determinations (threefold and 11-point IC_50_ curves) were performed using an ECHO 550 acoustic dispenser (Labcyte) and dry dispensed into 384-well polypropylene assay plates. The final assay concentration of DMSO was kept constant at 0.5% (*v*/*v*). Full-length JMJD6 was prepared at a concentration of 1.0 μM in 50 mM Tris-Cl, pH 7.5 and 25 μl dispensed across the 384-well plates, JMJD6 was pre-incubated with compound dilutions for 15 min. The reaction was initiated by dispensing 25 μl of a substrate solution (20 μM FAS, 200 μM L-AA, 10 μM RBM39_31–42_ and 20 μM 2OG) across each 384-well assay plate. The reaction was allowed to progress for 30 min, then halted by dispensing 10% (*v*/*v*) aqueous formic acid (5 μl per well). Assay plates were analyzed by LC–MS as described in the section ‘Peptide detection by LC–MS’.

### OGFOD1 SPE-MS assays

The inhibition of OGFOD1 activity was measured using a 30-mer peptide substrate of the ribosomal protein RPS23 (RPS23_47–76_). IC_50_ determinations were performed in 384-well-plate format using polypropylene plates (Greiner Bio One, cat. no. 781096). Compounds were prepared as 20 mM DMSO stock solutions and all compound dispenses were performed using an ECHO 550 acoustic dispenser (Labcyte). A positive control compound (2,4-PDCA, 100 mM) was dispensed into column 1 (250 nl) and DMSO was dispensed into column 13 (250 nl). All test compounds were serially diluted (an approximately threefold dilution series across an 11-point IC_50,_ 100 μM to 0.0017 μM) and 250 nl of each dilution dispensed in duplicate into the polypropylene plate. OGFOD1 was diluted to 0.3 μM in assay buffer (50 mM Tris-Cl, pH 7.5) and was dispensed (25 μl) into the 384-well compound plates using a multidrop combi reagent dispenser (Thermo Scientific, 5840300) with a small-tube plastic-tip dispensing cassette (Thermo Scientific, 24073290). The compounds were pre-incubated with OGFOD1 for 15 min and the reaction was initiated by dispensing 25 μl of a substrate solution (200 μM L-AA, 20 μM FAS, 20 μM 2OG and 10 μM RPS23 (47–76) peptide) in assay buffer. The final concentration of DMSO in the assay was 0.5%. The reactions were allowed to progress for 20 min, then halted by addition of 10% (*v*/*v*) formic acid (5 μl). Assay plates were transferred to an Agilent RapidFire RF365 and processed as described in the section below (RapidFire SPE-MS procedure).

### Lysine demethylase LC–MS assay

The inhibitory activity of IOX5 was measured by monitoring demethylation of peptide substrates for KDM4A, KDM5B and KDM6B. The peptide substrate for KDM4A was a 15-mer histone-H3 derivative (ARTAQTARK(me3)STGGIA) as reported previously^81^ and synthesized by GL Biochem. The peptide substrate for KDM5B was a 21-mer histone-H3 peptide (ARTK(me3)QTARKSTGGKAPRKQLA), synthesized by Peptide Protein Research. The KDM6B peptide substrate was a 17-mer histone-H3 peptide (LATKAARK(me3)SAPATGGVK), synthesized by GL Biochem.

### KDM4A LC–MS assays

KDM4A reactions were performed under optimized buffer conditions (50 mM MES, pH 7.0). KDM4A (0.15 μM) was pre-incubated for 15 min in the presence of IOX5 (100 μM) and the enzyme reaction initiated by addition of a substrate solution (100 μM L-AA, 10 μM FAS, 10 μM 2OG and 10 μM peptide substrate). The reaction was progressed for 50 min and then stopped by the addition of formic acid to a final concentration of 1% (*v*/*v*). Control reactions in the presence of 0.5% (*v*/*v*) DMSO and a control in the presence of a known inhibitor of KDM4A (50 μM 2, 4-pyridine dicarboxylic acid^82^).

### KDM5B LC–MS assays

KDM5B enzyme reactions were performed under optimized buffer conditions (50 mM MES, pH 7.0, 50 mM NaCl and 1 mM TCEP). KDM5B (0.15 μM) was pre-incubated for 15 min in the presence of IOX5 (100 μM) and the enzyme reaction was initiated by addition of substrate (100 μM L-AA, 10 μM FAS, 10 μM 2OG and 5 μM peptide). The enzyme reaction was progressed for 30 min, then halted by addition of formic acid to a final concentration of 1% (*v*/*v*). Control reactions included a 0.5% DMSO control and a reaction with a known inhibitor of KDM5B (10 μM KDOAM25 (ref. ^83^)).

### KDM6B LC–MS assays

KDM6B reactions were performed under optimized buffer conditions (50 mM MES, pH 7.0). KDM6B (0.15 μM) was pre-incubated for 15 min in the presence of IOX5 (100 mM) and the enzyme reaction initiated by addition of substrate (100 μM l-ascorbate, 10 μM ferrous ammonium sulfate, 10 μM 2OG and 5 μM peptide). The enzyme reaction was progressed for 30 min, then halted by addition of formic acid to a final concentration of 1% (*v*/*v*). Control reactions included a 0.5% DMSO control and a reaction with a known inhibitor of KDM6B (10 μM GSKJ1 (ref. ^84^)).

### Peptide detection by LC–MS

KDM4A, KDM5B and KDM6B reactions were transferred to a 96-well polypropylene plate and peptide analysis was performed by LC–MS using an Agilent 1290 infinity II LC system equipped with an Agilent 1290 multisampler and an Agilent 1290 high-speed pump and connected to an Agilent 6550 Accurate Mass iFunnel quadrupole-time of flight (QTOF) mass spectrometer. Then, 4 μl of the enzyme reaction was injected and loaded onto a ZORBAX RRHD Eclipse Plus C18 column (Agilent Technologies). Solvent A consisted of LC–MS grade water containing 0.1% (*v*/*v*) formic acid and solvent B consisted of acetonitrile containing 0.1% (*v*/*v*) formic acid. Peptides were separated using a step-wise gradient (0 min in 95% solvent A; 1.0 min in 80% solvent A; 3.0 min in 45% solvent A; 4.0 min in 45% solvent A; 5.0 min in 0% solvent A; 6.0 min in 0% solvent A; and 7.0 min in 95% solvent A). This was followed by a 3-min post run with 95% solvent A to re-equilibrate the column; all flow rates were 0.2 ml min^−1^. The mass spectrometer was operated in the positive ion mode with a drying gas temperature of 280 °C, drying gas flow rate of 13 l min^−1^, nebulizer pressure of 40 psig, sheath gas temperature of 350 °C, sheath gas flow rate of 12 l min^−1^, capillary voltage of 4,000 V and nozzle voltage of 1,000 V. All acquired data were analyzed using Agilent MassHunter Qualitative Analysis (v.B.07.00) software.

### RapidFire SPE-MS procedure

Assay plates were then transferred to an Agilent RapidFire RF365 machine connected to an Agilent 6550 Accurate Mass iFunnel QTOF mass spectrometer. Samples were aspirated under vacuum, loaded onto a C4 SPE cartridge and then washed with 0.1% (*v*/*v*) aqueous formic acid to remove buffer salts. Peptides were eluted from the C4 SPE cartridge using 0.1% (*v*/*v*) formic acid in 85% (*v*/*v*) acetonitrile and 15% (*v*/*v*) LC–MS-grade water onto the QTOF mass spectrometer. The mass spectrometer was operated in the positive ion mode with a drying gas temperature of 280 °C, drying gas flow rate of 13 l min^−1^, nebulizer pressure of 40 psig, sheath gas temperature of 350 °C, sheath gas flow rate of 12 l min^−1^, capillary voltage of 4,000 V and nozzle voltage of 1,000 V. Ion chromatogram data for the most predominant charge state was extracted and peak area data for the substrate peptide and hydroxylated peptide integrated using RapidFire Integrator (Agilent, v.4.3.0.17235). The percentage conversion was calculated using Excel and IC_50_ curves generated using GraphPad Prism (v.5.04).

### Biophysical methods

#### NMR spectroscopic studies

NMR spectra were measured using a Bruker AVIII 700 MHz NMR spectrometer equipped with a TCI inverse cryoprobe using 3-mm diameter high-throughput NMR tubes (Norell). Samples were recorded at 298 K. Data were processed with TopSpin v.3.6.2 software.

#### ^1^H CPMG NMR experiments

For ^1^H Carr–Purcell–Meiboom–Gill (CPMG) NMR spectroscopy, the assay mixtures contained 50 µM *apo*-PHD2_181-426_, 200 µM Zn (II), 50 µM 2OG, increasing concentrations of ligand (up to 400 µM) in 50 mM Tris-D_11_, 150 mM NaCl (pH 7.5), in 90 % H_2_O and 10 % D_2_O (*v*/*v*). Typical experimental parameters for CPMG NMR spectroscopy were total echo time, 100 ms (τ = 1 ms, *n* = 50); number of points, 32,768; sweep width, 16 ppm; relaxation delay, 2 s; and number of transients, 64. The PROJECT-CPMG sequence (90°_*x*_-(τ -180°_*y*_-τ -90°_*y*_-τ -180°_*y*_-τ)_*n*_-acq) was employed. Pre-saturation was used to achieve water suppression^15^.

### Western blot assays

For immunoblotting in HEK293T cells, cells were treated with increasing concentrations of IOX5, 20 μM of roxadustat or vehicle control (as indicated in Fig. 4d) using cell lysis buffer (1× RIPA buffer, Sigma, R0278) supplemented with protease inhibitors (Complete, Mini, EDTA-free Protease Inhibitor Cocktail, Roche). Protein extracts were subjected to SDS–PAGE separation (NuPAGE 4–12% Bis-Tris Plus Gel, Thermo Fisher Scientific, NP0323BOX), then transferred onto a polyacrylamide membrane using wet transfer. The gels were run in 1× Tris/glycine/SDS running buffer (20× NuPAGE MES SDS Running Buffer, Life Technologies) at 180 V for 45 min (Mini Gel Tank, Life Technologies). Membranes were blocked with 5% milk powder in 1× PBS-T for 30 min then incubated overnight at 4 °C with anti-HIF-1α (BD Biosciences, cat. no. 610959, 1:1,000, ON at 4 °C) and anti-GAPDH (Invitrogen, cat no. MA5-15738, 1:1,000, ON at 4 °C). After incubation with appropriate horseradish peroxidase-coupled secondary antibody (Cell Signaling Technology, rabbit anti-mouse IgG, (D3V2A), 1:5,000, 2 h at room temperature), proteins were detected with by GE Healthcare Amersham ECL Prime Western Blotting Detection Reagent (RPN2236) and acquired on the Bio-Rad Universal Hood III.

For immunoblotting in AML cells, MOLM13, OCI-AML3, MV411 and THP-1 cells treated with 50 μM Dap, 50 μM IOX5 or vehicle control using cell lysis buffer (Cell Signaling Technology, 9803) supplemented with protease and phosphatase inhibitors (Merck, 20-201, 524624). Total protein extracts (30 or 60 μg) were subjected to SDS–PAGE separation (NuPAGE 4–12% Bis-Tris Plus Gel, Thermo Fisher Scientific, NP0323BOX) and then transferred onto polyvinylidene difluoride membranes using wet transfer. Membranes were blocked in 5% milk-TBST (TBS with 0.1% Tween20) and probed with anti-HIF-1α (BD Biosciences, 610959, 1:1,000, ON at 4 °C), anti-HIF-2α (Cell Signaling Technology, 59973, 1:1,000, ON at 4 °C), anti-BNIP3 (Abcam, EPR4034, 1:2,000, ON at 4 °C), anti-β-actin (Cell Signaling Technology, 3700, 1:10,000, 30 min at room temperature) and anti-histone-H3 (Cell Signaling Technology, 4499, 1:2,000, 30 min at room temperature). After incubation with the appropriate horseradish peroxidase-coupled secondary antibody (Cell Signaling Technology, Anti-mouse IgG, 7076, 1:2,000, 2 h at room temperature or anti-rabbit IgG, 7074, 1:2,000, 2 h at room temperature), proteins were detected with Clarity Western ECL Substrate (Bio-Rad, 1705061) and acquired using an Amersham Imager 600 (GE Healthcare Life Sciences). Unprocessed western blots are available in the Source Data.

### Real-time quantitative PCR assays

RNA was isolated using Direct-zol RNA Miniprep kit (Zymo Research, R2051) and reverse transcribed using High-Capacity cDNA Reverse Transcription kit (Applied Biosystems). For real-time quantitative PCR (qPCR), 5 ng of cDNA, 5 µl PowerUp SYBR Green MasterMix (Applied Biosystems) and 2 pmol of primers were used per well of 384-well plate. Reactions were performed in triplicate using C1000 Thermocycler 384-well (Bio-Rad). Gene expression was quantified using the comparative ΔΔ-Ct method and *ACTB* was used as the housekeeping gene. Data are expressed as log_2_ fold change in comparison to control sample and represents results of three independent experiments measured in duplicate.

### shRNA-mediated *BNIP3* knockdown

MOLM13 cells were transduced with lentiviruses expressing shRNAs, shRNA *BNIP3*, 5′-GCCTCGGTTTCTATTTATAAT-3′ (TRCN0000007831, Sigma-Aldrich); and shRNA CTL, 5′-TTCTCCGAACGTGTCACGTT-3′ (GE Healthcare). Selection of efficiently transduced cells was achieved by treatment with puromycin (2 μg ml^−1^ final concentration).

### RNA extraction, sequencing and bioinformatic analyses

Total RNA was extracted from 500,000 leukemic cells using RNeasy Plus Universal Mini kit (QIAGEN, cat. no. 73404) following the manufacturer’s protocol. The RNA integrity number (RIN) was determined by High Sensitivity RNA ScreenTape analysis (Agilent, cat. no. 5067) and all RIN was >8. High sensitivity libraries for RNA-seq were prepared from 500 ng of total RNA using the NEBNext Ultra II Directional RNA Library Prep kit for Illumina (NEB, cat. no. E7760) with NEBNext Poly(A) mRNA Magnetic Isolation Module (NEB, cat. no. E7490) following the manufacture’s protocol. Nine cycles of PCR were performed to amplify libraries and the pooled library was sequenced on Illumina NextSeq 500 with 85 bp single-end mode at EMBL GeneCore facility.

For analysis of differentially expressed genes, adaptors were trimmed with Cutadapt (v.1.18) using options: -a AGATCGGAAGAGC -m 50, and processed reads were mapped to GRCm38 genome_tran (release 84) with HISAT2 (v.2.2.1)^85^ using the option –qc-filter. Mapped reads per gene were counted with htseq-count (HTSeq v.2.0.1)^86^ providing GTF file and differentially expressed genes were analyzed using DESeq2 (v.1.30.1).

### GSEA and ingenuity pathway analysis

Gene set enrichment analysis (GSEA) was performed using GSEA software v.3.0 with 1,000 permutations and default parameters. Gene differential expression, computed by the edgeR package in R, was ranked by moderated *t* statistics, which takes into account variability between genes in the ranking. Ranked genes were compared to gene lists in the Hallmark subset of the MSigDB database, v.7.0. Ingenuity pathway analysis was performed using the Core Analysis Function offered by QIAGEN’s Ingenuity Pathway Analysis software. The interrogated RNA-seq and MS datasets were filtered for adjusted *P* values of differential expression (false discovery rate < 0.05) and the threshold for significant activation or inhibition was defined by an absolute *z*-score >2.

### Statistics and reproducibility

Statistical analyses were performed using GraphPad Prism v.9 software (GraphPad Software).

The study design was based on previous experience and experiments using similar approaches and thus no statistical methods were used to predetermine sample sizes for this study^6,47,58,67,68^. In vivo transplantation experiments utilizing genetically altered murine cells were randomized and blinded to the primary researcher during the experiment and analyses. In vivo experiments utilizing PHD inhibitors were not blinded due to the primary researcher dosing animals, but unbiased monitoring and scoring was performed by independent staff members. For in vitro experiments, samples were equally allocated to ensure that covariates were identical between groups. The investigators performing in vitro experiments were not blinded during allocation or outcome assessment. Experiments were replicated by independent researchers who were initially unaware of the expected outcome. Data distribution was assumed to be normal, but this was not formally tested. No animals or data points were excluded for any reason. Numbers (*n*) are provided for each experiment and specified if different between groups.

### Reporting summary

Further information on research design is available in the Nature Portfolio Reporting Summary linked to this article.

### Supplementary information

Supplementary information Supplementary Figs. 1–17 and legends.

Reporting Summary

Supplementary Tables Supplementary Tables 1 and 2.

### Source data

Source Data Fig. 1 Statistical source data for Fig. 1.

Source Data Fig. 2 Statistical source data for Fig. 2.

Source Data Fig. 3 Statistical source data for Fig. 3.

Source Data Fig. 5 Statistical source data for Fig. 5.

Source Data Fig. 6 Statistical source data for Fig. 6.

Source Data Fig. 7 Statistical source data for Fig. 7.

Source Data Fig. 8 Statistical source data for Fig. 8.

Source Data Extended Data Fig. 1 Statistical source data for Extended Data Fig. 1.

Source Data Extended Data Fig. 2 Statistical source data for Extended Data Fig. 2.

Source Data Extended Data Fig. 4 Statistical source data for Extended Data Fig. 4.

Source Data Extended Data Fig. 5 Statistical source data for Extended Data Fig. 5.

Source Data Extended Data Fig. 6 Statistical source data for Extended Data Fig. 6.

Source Data Extended Data Fig. 7 Statistical source data for Extended Data Fig. 7.

Source Data Extended Data Fig. 8 Statistical source data for Extended Data Fig. 8.

All figures Unprocessed western blot gels.

## Data Availability

RNA-seq data that support the findings of this study have been deposited in the Gene Expression Omnibus under accession code GSE232644. Human patient data analyzed in Fig. 1a,b were obtained from Nehme et al. under accession code GSE147515. Human patient data analyzed in Extended Data Fig. 1a were obtained from http://www.vizome.org/ under accession ID phs001657.v1.p1. Source data for Figs. 1–8 and Extended Data Figs. 1–8 have been provided as Source Data files. All other data supporting the findings of this study are available from the corresponding authors upon request. Source data are provided with this paper.

## Code availability

All analysis was performed using freely available R packages. Plots were generated with software and pipelines that have been in use in previous papers. No special-purpose bioinformatic algorithms were used.
