# Supplementary material for: The selective prolyl hydroxylase inhibitor IOX5 stabilizes HIF-1α and compromises development and progression of acute myeloid leukemia
Source: Nat Cancer. 2024 Apr 18;5(6):916–37. doi: 10.1038/s43018-024-00761-w (PMC11208159; doi:10.1038/s43018-024-00761-w)
Supplement: Supplementary file 1 — Supplementary Figs. 1–17 and legends. [file 43018_2024_761_MOESM1_ESM.pdf]

nature cancer

Article https://doi.org/10.1038/s43018-024-00761-w

The selective prolyl hydroxylase inhibitor IOX5 stabilizes HIF-1α and compromises development and progression of acute myeloid leukemia

In the format provided by the authors and unedited

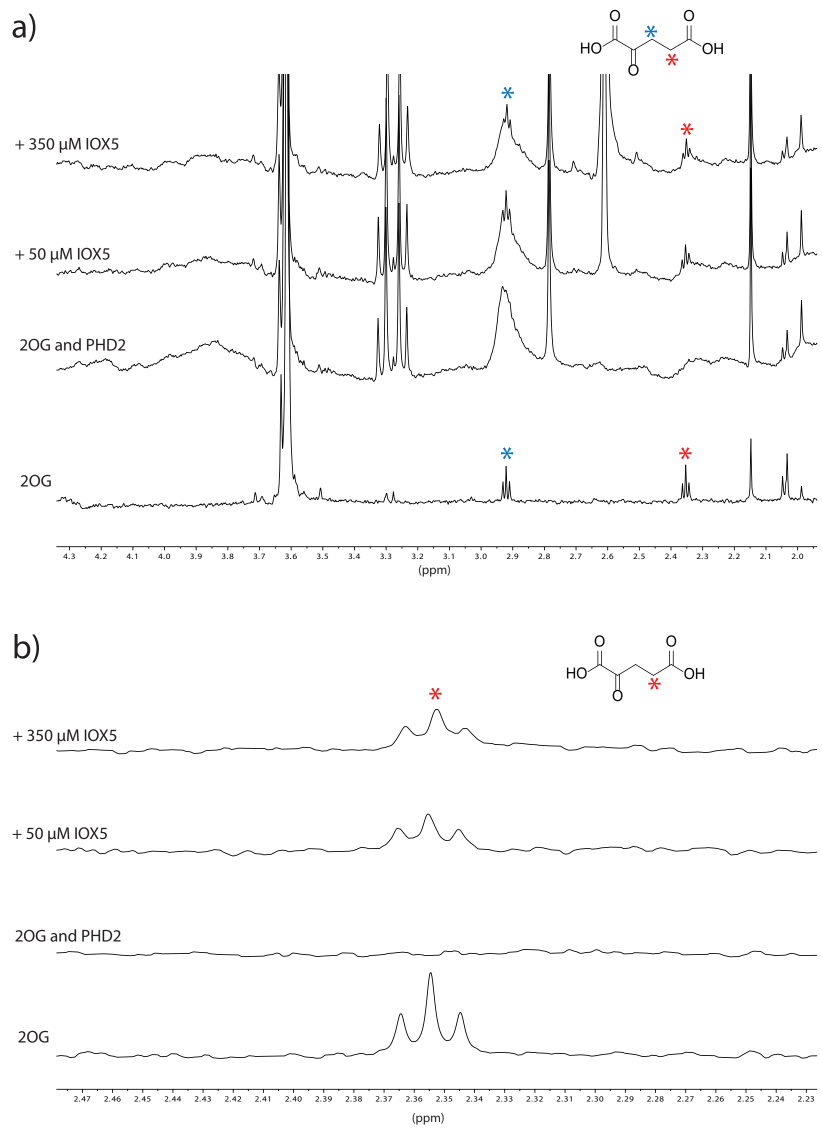

Supplementary Figure 1: NMR binding analyses showing IOX5 causes displacement of 2OG from the PHD2.2OG complex. 2OG followed by IOX5 were added to a PHD2 solution. 2OG displacement was qualitatively analysed by: **a)** ^1H NMR (700 MHz) excitation sculpting suppression, and **b)** CPMG-edited ^1H (700 MHz) analyses. The assay mixture contained PHD2 (50 µM) supplemented with 200 µM Zn (II), 50 µM 2OG, then IOX5 (50 µM or 350 µM), in 50 mM Tris-D_{11}, 150 mM NaCl (pH 7.5), in 90 % H_2O and 10 % D_2O *(v/v)*. The 2OG CH_2 peak at δ_H 2.35 ppm was monitored to measure 2OG signal recovery. Addition of IOX5 (50 µM or 350 µM) results in the recovery of the 2OG signal indicating 2OG displacement.

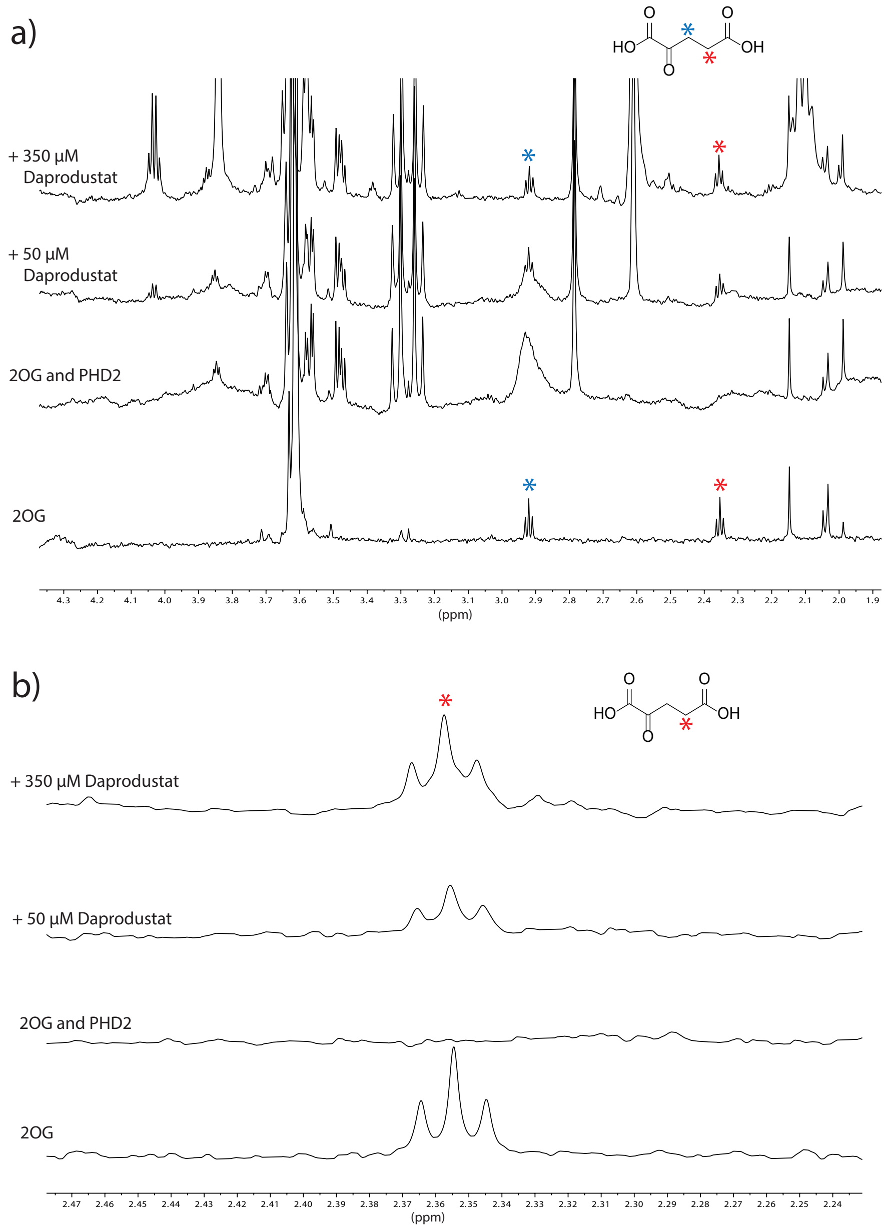

Supplementary Figure 2: NMR binding analyses showing Daprodustat causes displacement of 2OG from the PHD2.2OG complex. 2OG followed by Daprodustat were added to a PHD2 solution. 2OG displacement was qualitatively analysed by: **a)** ^{1}H NMR (700 MHz) excitation sculpting suppression, and **b)** CPMG-edited ^{1}H (700 MHz) analyses. The assay mixture contained PHD2 (50 µM) supplemented with 200 µM Zn (II), 50 µM 2OG**,** then Daprodustat (50 µM or 350 µM), in 50 mM Tris-D_{11}, 150 mM NaCl (pH 7.5), in 90 % H_2O and 10 % D_2O *(v/v)*. The 2OG CH_2 peak at δ_H 2.35 ppm was monitored **to** measure 2OG signal recovery. Addition of Daprodustat (50 µM or 350 µM) results in the recovery of the 2OG signal indicating 2OG displacement.

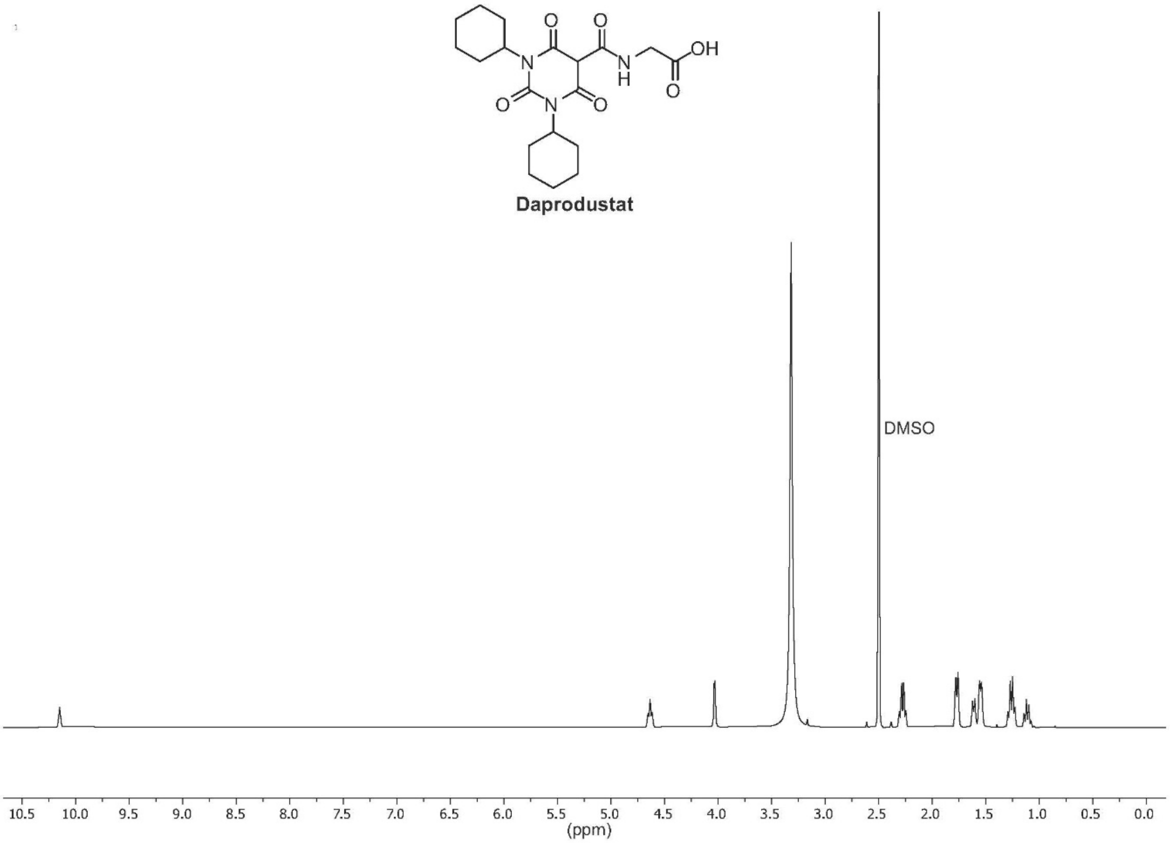

Supplementary Figure 3: ^{1}H (600 MHz) NMR spectrum for Daprodustat

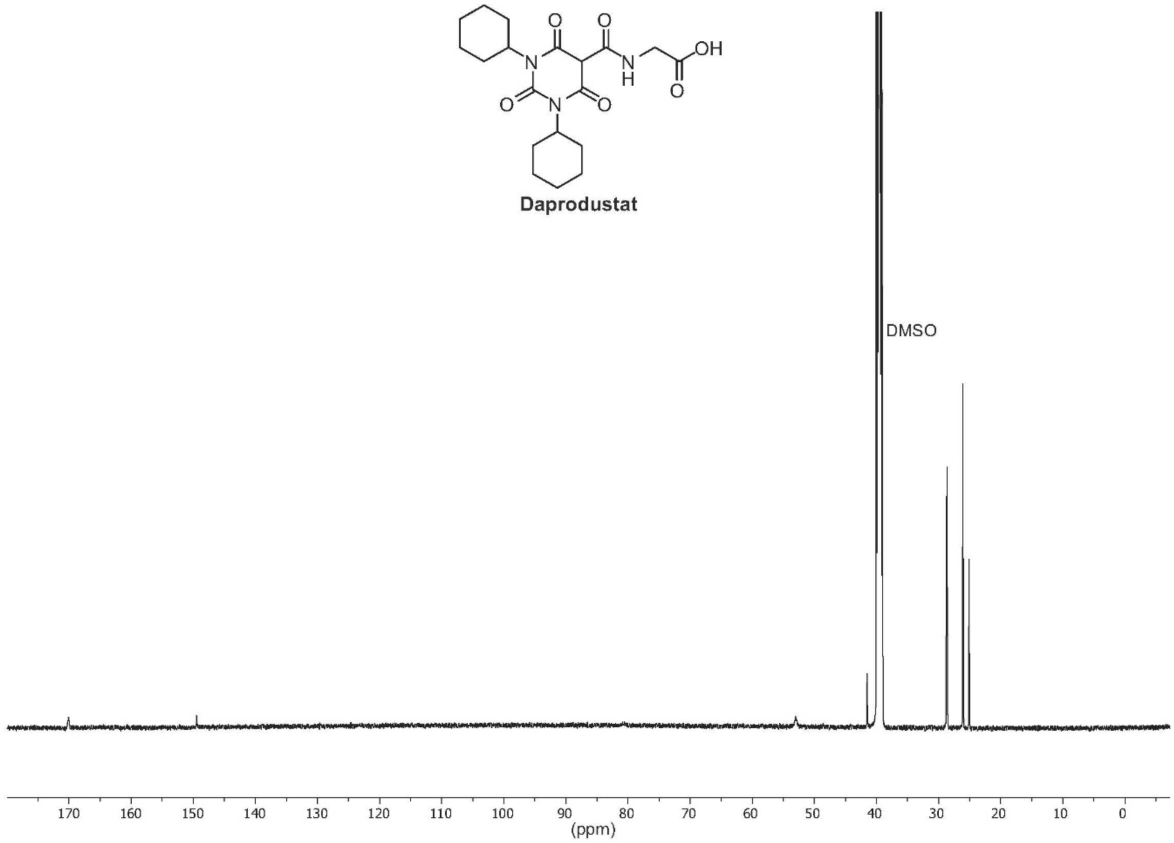

Supplementary Figure 4: ^{13}C (151 MHz) NMR spectrum for Daprodustat

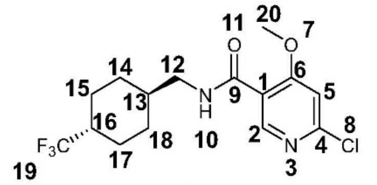

2a

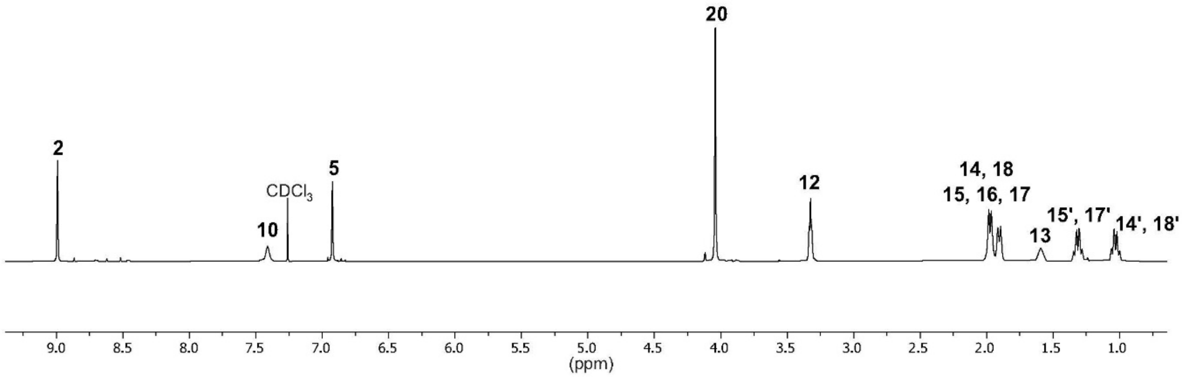

Supplementary Figure 5: ^{1}H (600 MHz) NMR spectrum for 2a

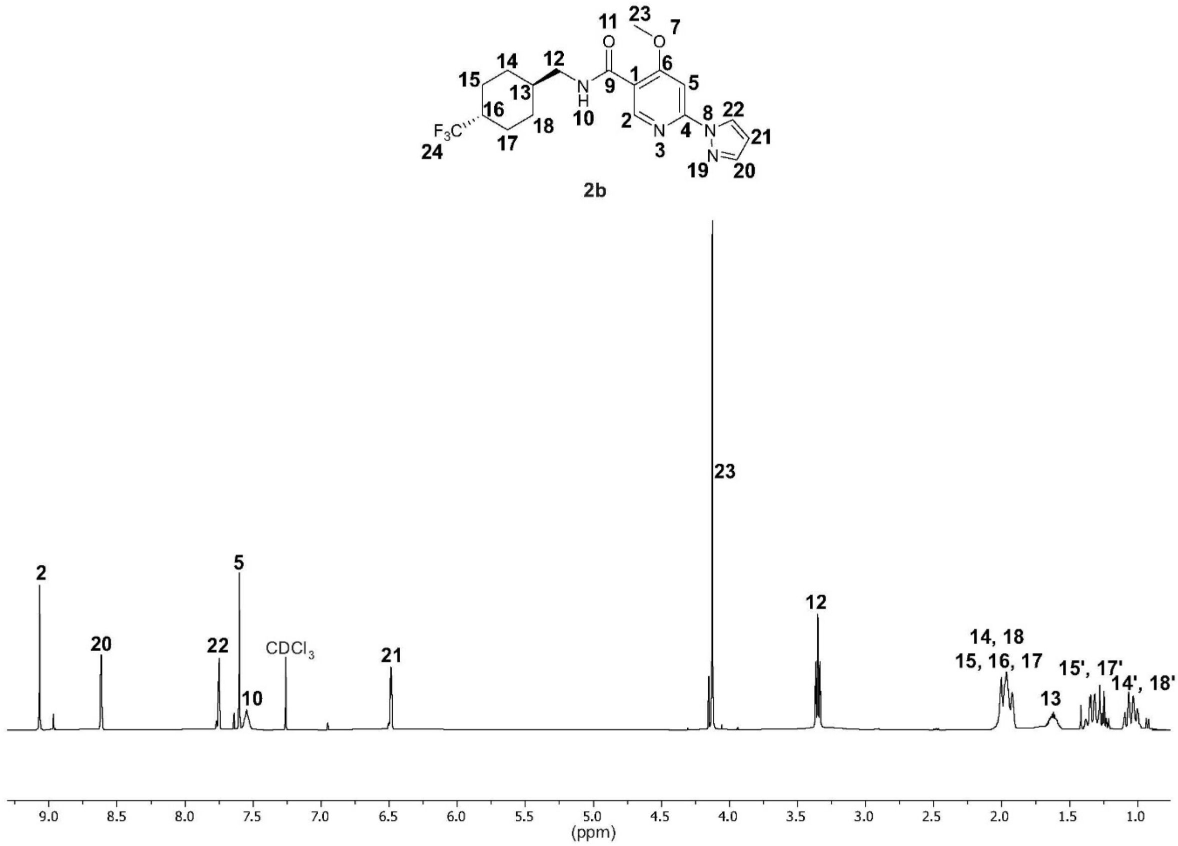

Supplementary Figure 6: ^{1}H (400 MHz) NMR spectrum for 2b

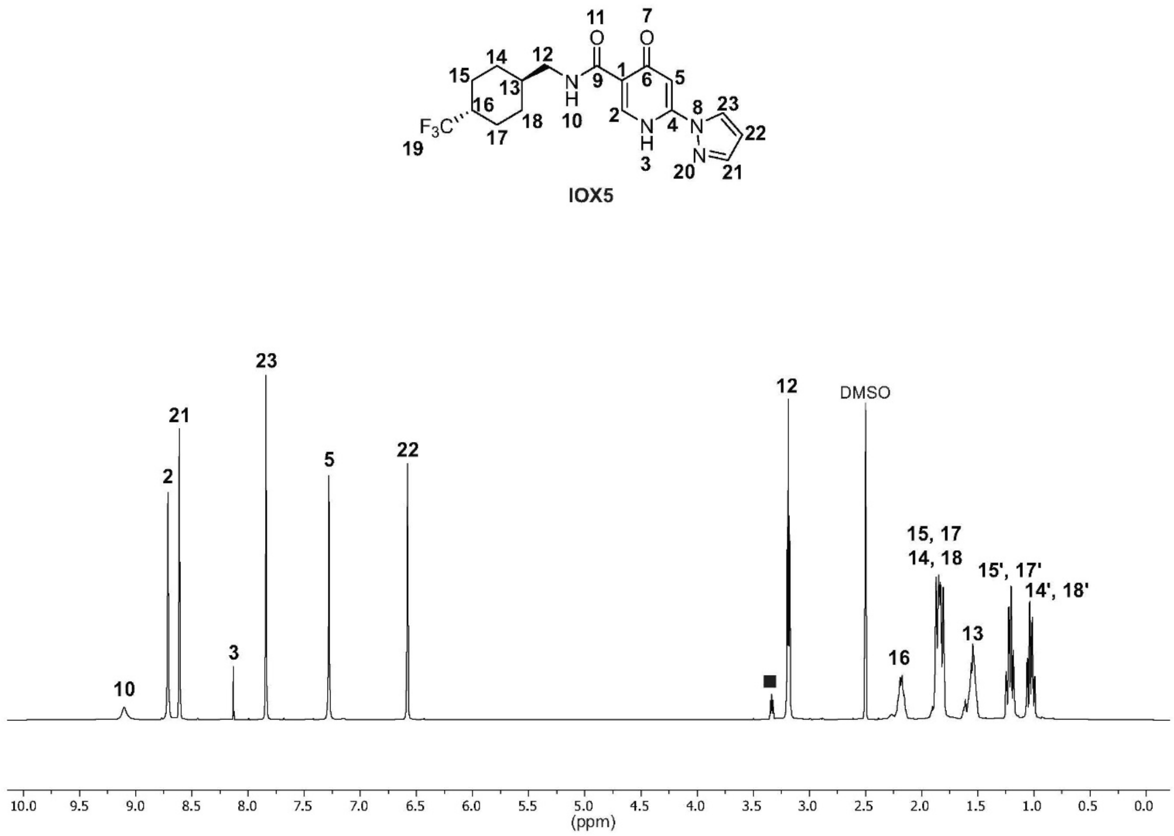

Supplementary Figure 7: ^{1}H (600 MHz) NMR spectrum for IOX5

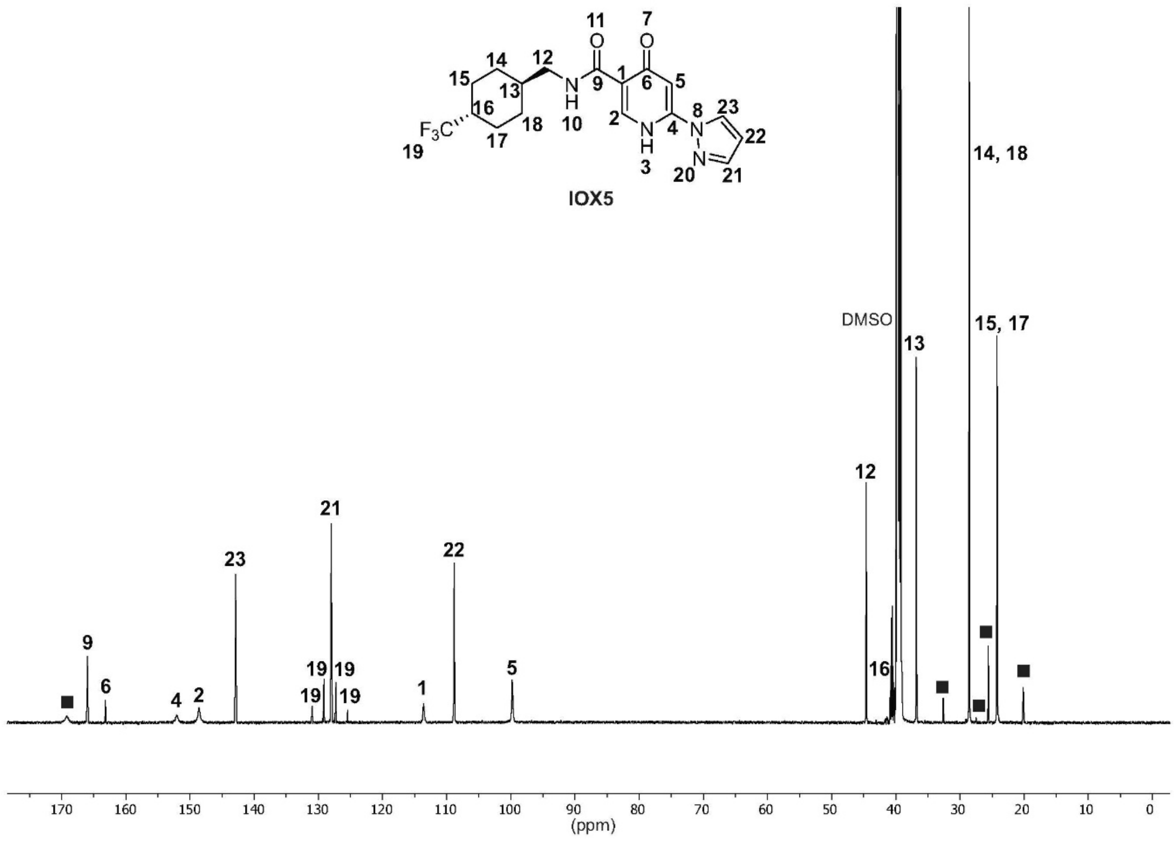

Supplementary Figure 8: ^{13}C NMR (151 MHz) spectrum for IOX5

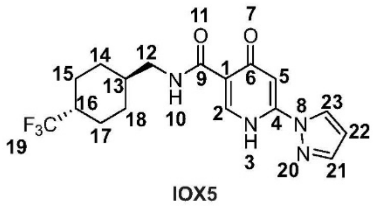

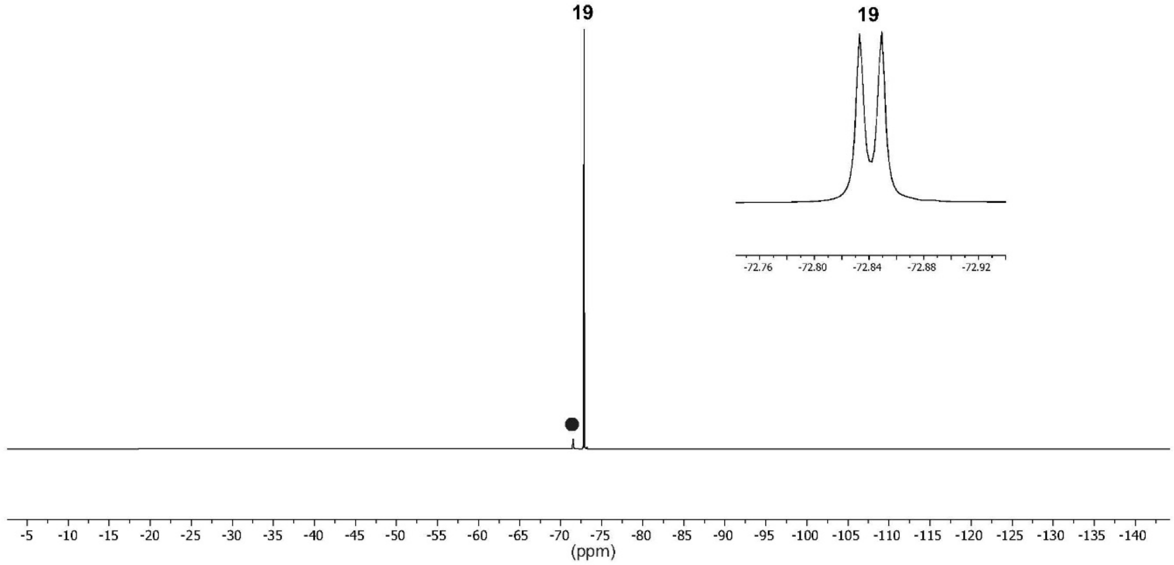

Supplementary Figure 9: ^{19}F NMR (565 MHz) spectrum for IOX5

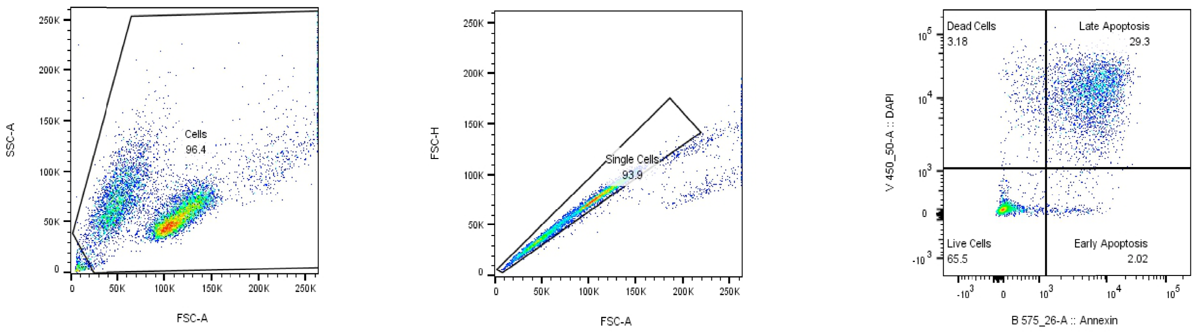

Supplementary Figure 10: Flow cytometry gating of Annexin/DAPI analyses.

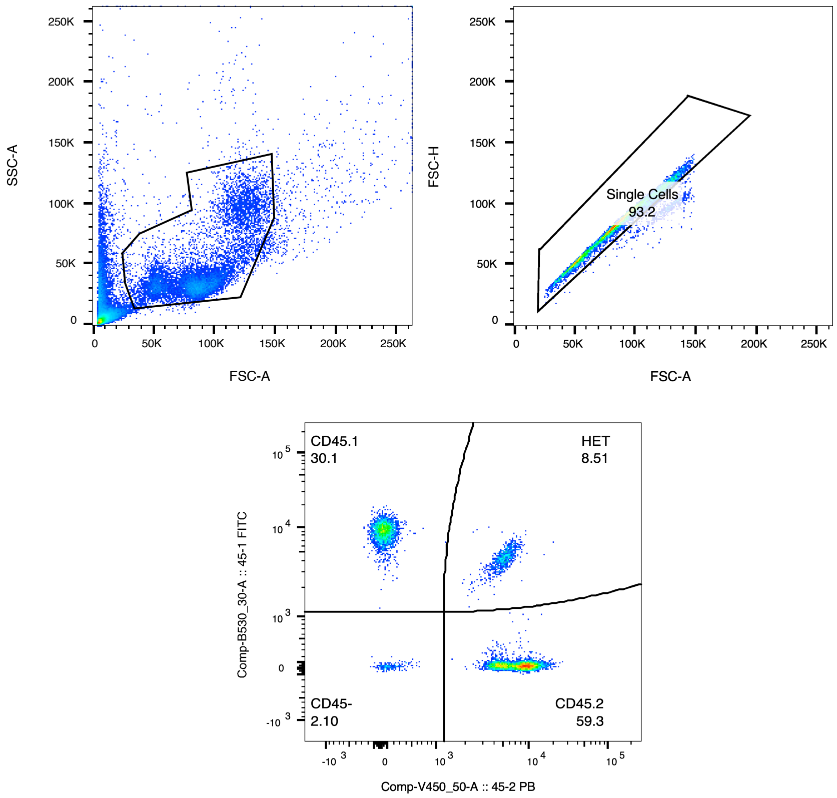

Supplementary Figure 11: Flow cytometry gating of CD45.2^{+} engraftment in the peripheral blood.

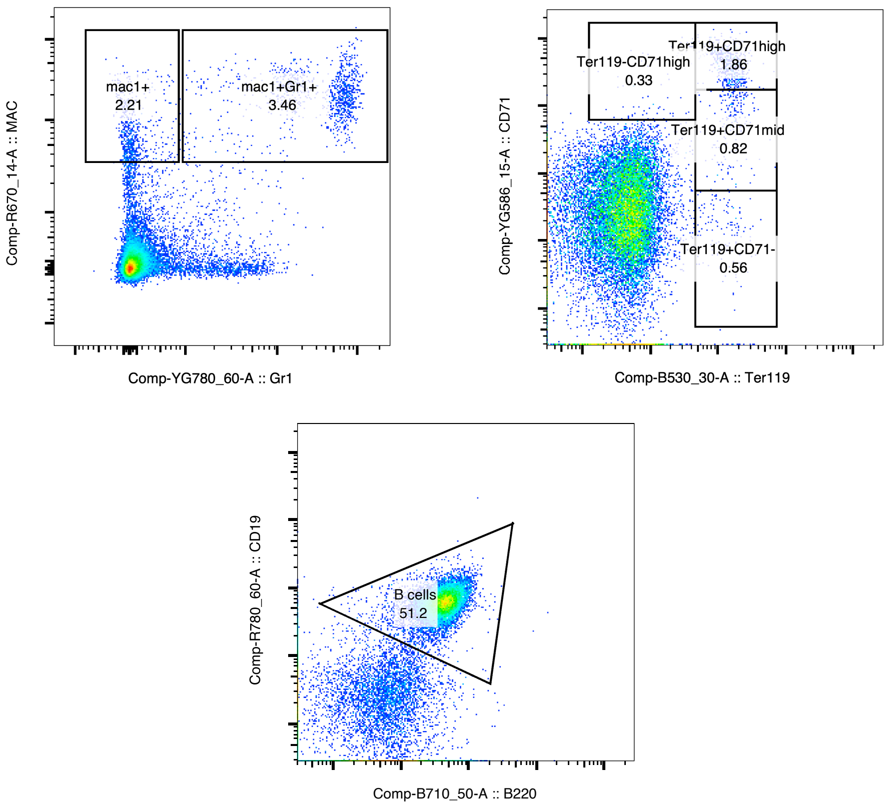

Supplementary Figure 12: Flow cytometry gating of differentiated haematopoietic compartments. Representative staining for Monocytes (Mac1^{+}), Granulocytes (Mac1^{+}Gr1^{+}), Erythroid staining (CD71 and Ter119) and B cells (CD19^{+}B220^{+}) is shown.

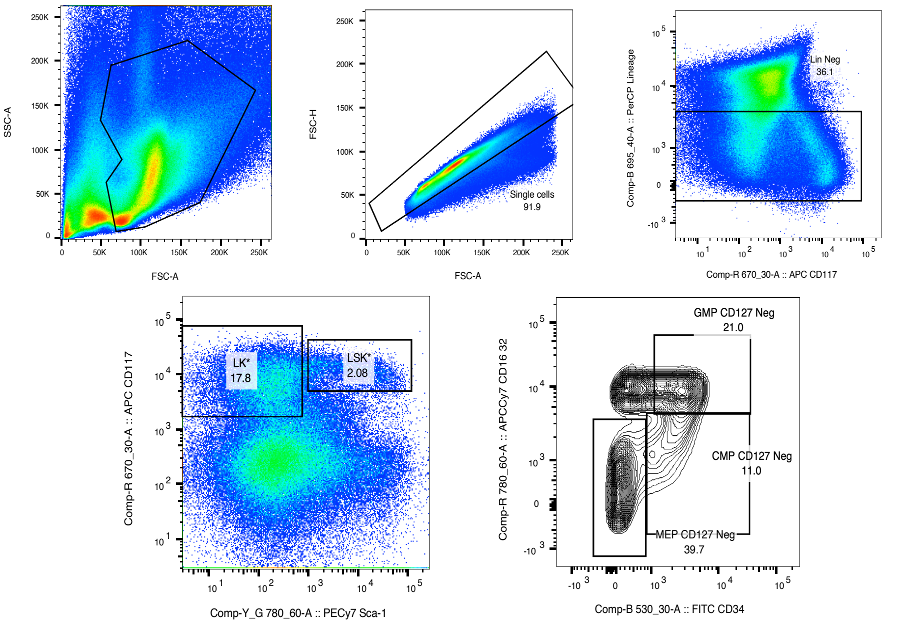

Supplementary Figure 13: Flow cytometry gating of haematopoietic progenitor compartments. Representative staining of LK (Lin$^-$c-Kit$^+$), LSK (Lin$^-$Sca-1$^+$c-Kit$^+$), CMP (LKCD34$^+$FcγRII/III^{low}), GMP (LKCD34$^+$FcγRII/III^{high}), and MEP (LKCD34$^-$FcγRII/III^{low}) is shown. FcγRII/III is also known as CD16/32.

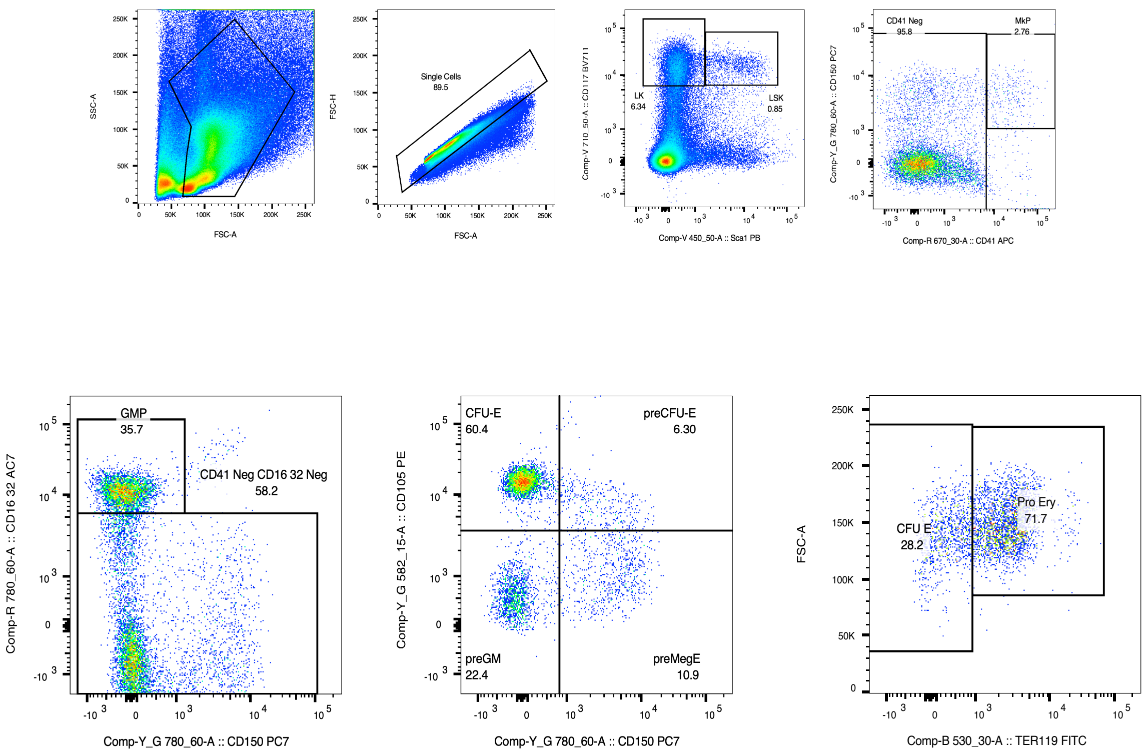

Supplementary Figure 14: Flow cytometry gating of haematopoietic progenitor compartments. Representative gating of LK (Lin$^-$c-Kit$^+$), LSK (Lin$^-$Sca-1$^+$c-Kit$^+$), MkP (LKCD41$^+$CD150$^+$), GMP (LKCD41$^-$FcγRII/III$^+$CD150$^-$), preCFU-E(LKCD41$^-$FcγRII/III$^-$CD105$^+$CD150$^+$), preMegE(LKCD41$^-$FcγRII/III$^-$CD105$^-$CD150$^+$), preGM(LKCD41$^-$FcγRII/III$^-$CD105$^-$CD150$^-$), CFU-E(LKCD41$^-$FcγRII/III$^-$CD105$^+$CD150$^-$Ter119$^-$), ProEry(LKCD41$^-$FcγRII/III$^-$CD105$^+$CD150$^-$Ter119$^+$) is shown.

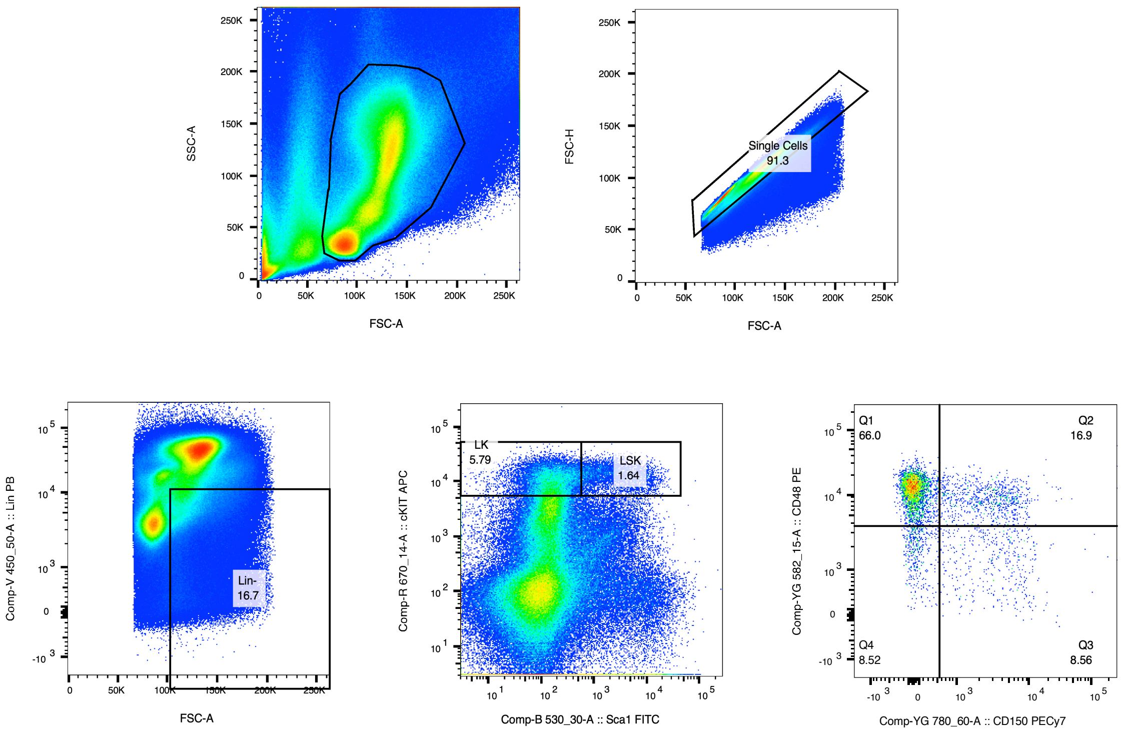

Supplementary Figure 15: Flow cytometry gating of haematopoietic stem and progenitor compartments. Representative gating of LK (Lin$^-$c-Kit$^+$), LSK (Lin$^-$Sca-1$^+$c-Kit$^+$), LSKCD48$^-$CD150$^+$ HSCs (Q3), LSKCD48$^-$CD150$^-$ MPPs (Q4), primitive HPCs (LSKCD48$^+$CD150$^-$ HPC-1 (Q1) and LSKCD48$^+$CD150$^+$ HPC-2 (Q2) populations) is shown.

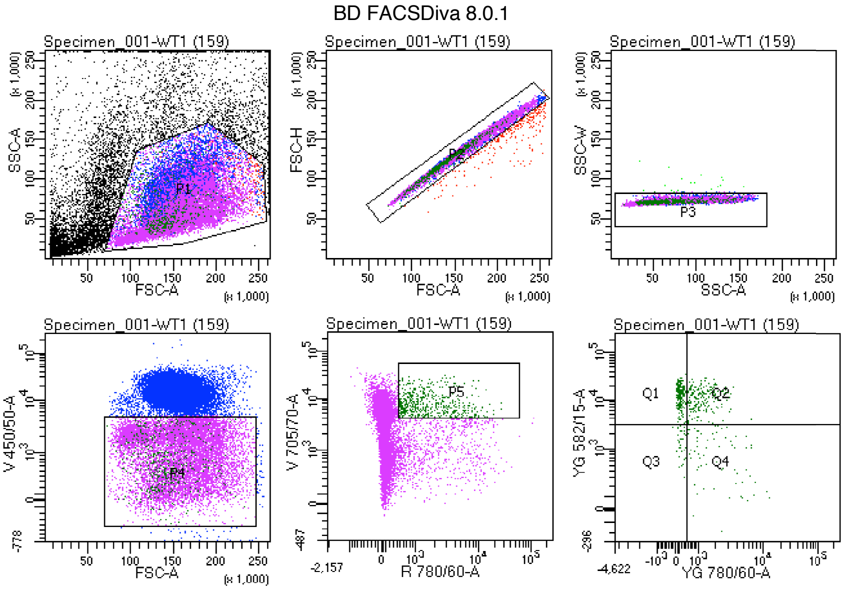

Supplementary Figure 16: Flow cytometry sorting strategy of haematopoietic stem cells for transplantation. Representative gating of LK (Lin$^-$c-Kit$^+$), LSK (Lin$^-$Sca-1$^+$c-Kit$^+$), LSKCD48$^-$CD150$^+$ HSCs (Q3), LSKCD48$^-$CD150$^-$ MPPs (Q4), primitive HPCs (LSKCD48$^+$CD150$^-$ HPC-1 (Q1) and LSKCD48$^+$CD150$^+$ HPC-2 (Q2) populations). V450/50 = Lin, V705/70 = c-Kit BV711, R780/60 = Sca-1 APC, YG582/15 = CD48 PE, YG780/60 = CD150 PECy7 is shown.

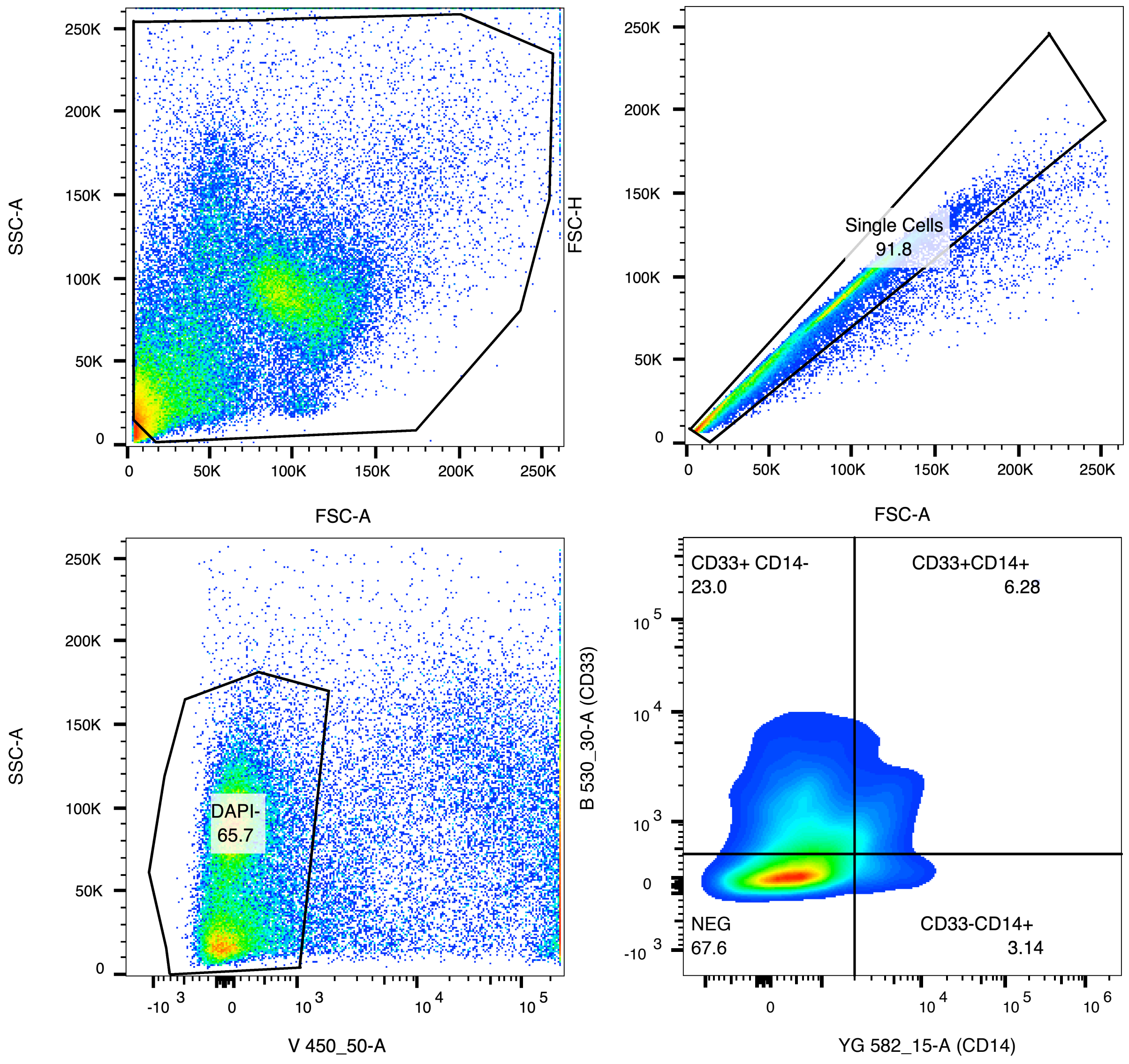

Supplementary Figure 17: Flow cytometry sorting strategy of human AML cell engraftment. CD33 and CD14 used as human AML cell markers.
