# Supplementary material for: The selective prolyl hydroxylase inhibitor IOX5 stabilizes HIF-1α and compromises development and progression of acute myeloid leukemia
Source: Nat Cancer. 2024 Apr 18;5(6):916–37. doi: 10.1038/s43018-024-00761-w (PMC11208159; doi:10.1038/s43018-024-00761-w)
Supplement: Supplementary file 2 — Reporting Summary [file 43018_2024_761_MOESM2_ESM.pdf]

nature portfolio

Corresponding author(s): Prof. Kamil R. Kranc & Prof. Christopher J. Schofield

Last updated by author(s): 18/2/24

Reporting Summary

Nature Portfolio wishes to improve the reproducibility of the work that we publish. This form provides structure for consistency and transparency in reporting. For further information on Nature Portfolio policies, see our Editorial Policies and the Editorial Policy Checklist.

Statistics

For all statistical analyses, confirm that the following items are present in the figure legend, table legend, main text, or Methods section.

n/a	Confirmed	
☐	☒	The exact sample size (*n*) for each experimental group/condition, given as a discrete number and unit of measurement
☐	☒	A statement on whether measurements were taken from distinct samples or whether the same sample was measured repeatedly
☐	☒	The statistical test(s) used AND whether they are one- or two-sided<br>*Only common tests should be described solely by name; describe more complex techniques in the Methods section.*
☐	☒	A description of all covariates tested
☐	☒	A description of any assumptions or corrections, such as tests of normality and adjustment for multiple comparisons
☐	☒	A full description of the statistical parameters including central tendency (e.g. means) or other basic estimates (e.g. regression coefficient) AND variation (e.g. standard deviation) or associated estimates of uncertainty (e.g. confidence intervals)
☐	☒	For null hypothesis testing, the test statistic (e.g. *F*, *t*, *r*) with confidence intervals, effect sizes, degrees of freedom and *P* value noted<br>*Give P values as exact values whenever suitable.*
☒	☐	For Bayesian analysis, information on the choice of priors and Markov chain Monte Carlo settings
☒	☐	For hierarchical and complex designs, identification of the appropriate level for tests and full reporting of outcomes
☐	☒	Estimates of effect sizes (e.g. Cohen's *d*, Pearson's *r*), indicating how they were calculated

Our web collection on statistics for biologists contains articles on many of the points above.

Software and code

Policy information about availability of computer code

Data collection	BDS FACS Diva v9.0<br>Amersham 600 RGB imager (chemidoc)<br>BioRad CFX Manager
Data analysis	FlowJo v10 6.1<br>GraphPad Prism v9<br>Microsoft Excel 2019<br>RStudio with ggplot2<br>QIAGEN (Ingenuity Pathway Analysis software)<br>Chemdraw 3D<br>Pymol<br>Agilent MassHunter Qualitative Analysis (Version B.07.00)<br>Agilent RapidFire Integrator<br>GRCm38 genome_tran (release 84) with HISAT2 (version 2.2.1)<br>htseq-count (HTSeq version 2.0.1)<br>DESeq2 (version 1.30.1)<br>MSigDB database, version 7.0

For manuscripts utilizing custom algorithms or software that are central to the research but not yet described in published literature, software must be made available to editors and reviewers. We strongly encourage code deposition in a community repository (e.g. GitHub). See the Nature Portfolio guidelines for submitting code & software for further information.

Data

Policy information about availability of data

All manuscripts must include a data availability statement. This statement should provide the following information, where applicable:

- Accession codes, unique identifiers, or web links for publicly available datasets
- A description of any restrictions on data availability
- For clinical datasets or third party data, please ensure that the statement adheres to our policy

An updated data availability statement is in the manuscript as follows: "RNA-seq data that support the findings of this study have been deposited in the Gene Expression Omnibus (GEO) under accession codes GSE232644. Human patient data analysed in Fig. 1a-b were obtained from Nehme et al. under the accession code GSE147515. Human patient data analysed in Extended Data Fig. 1a were obtained from http://www.vizome.org/, accession ID phs001657.v1.p1. Source data for Fig. 1-8 and Extended Data Fig. 1-8 have been provided as Source Data files. All other data supporting the findings of this study are available from the corresponding authors upon request."

Research involving human participants, their data, or biological material

Policy information about studies with human participants or human data. See also policy information about sex, gender (identity/presentation), and sexual orientation and race, ethnicity and racism.

Reporting on sex and gender	This study analysed the response of AML patient samples to compounds, independent of their sex, gender, race, age. The relevant biological parameters, i.e. mutational status as are included in Extended Data Figure 1. Additional information can be requested from Barts Cancer Institute Haemato-Oncology Research Tissue Bank.
Reporting on race, ethnicity, or other socially relevant groupings	As above - race, ethnicity or social background of AML sample donors was not taken into account in this study.
Population characteristics	A total of 13 patient samples were used in this study.
Recruitment	As above
Ethics oversight	All use of human tissue was in compliance with the ethical and legal framework of the United Kingdom's Human Tissue Act, 2004. Primary human AML samples were from Barts Cancer Institute Biobank (with approval of the Research Ethics Committee). Their use was authorised following ethical review by the Tissue Biobank's scientific sub-committee, and with the informed written consent of the donor. For all human AML samples used in this study, Barts Cancer Institute Biobank obtained informed written consent from all participants.

Note that full information on the approval of the study protocol must also be provided in the manuscript.

Field-specific reporting

Please select the one below that is the best fit for your research. If you are not sure, read the appropriate sections before making your selection.

☒ Life sciences ☐ Behavioural & social sciences ☐ Ecological, evolutionary & environmental sciences

For a reference copy of the document with all sections, see nature.com/documents/nr-reporting-summary-flat.pdf

Life sciences study design

All studies must disclose on these points even when the disclosure is negative.

Sample size	No power calculation was performed for in vitro experiments. Number of independent repeats was determined based on previous experience. For in vivo experiments, number of mice per group were based on power calculations based on extensive previous experience with these in vivo techniques. Previous publications using these power calculations include; https://doi.org/10.1084/jem.20150452, https://doi.org/10.1084/jem.20161087, https://doi.org/10.1182/bloodadvances.2020002702 and https://doi.org/10.1016/j.stem.2019.03.021.
Data exclusions	No data was excluded
Replication	The number of replicates (technical or biological) are stated in the figure legends. All data presented was independently replicated at least twice, often using an independent researcher.
Randomization	For in vivo work involving steady-state or transplantation analyses, all animals were randomised, i.e. all cages included both WT or experimental animals. For in vivo work involving pharmacological treatment, animals were randomised and grouped based on their initial weights, initial engraftment or gender. For in vitro experiments, samples were equally allocated to ensure that covariates were identical between groups.
Blinding	Where possible during the in vivo studies, the primary researcher was blinded. This was done by randomising the animals where possible and ensuring the majority of animal checks were carried out by independent researchers. Blinding was often not possible in in vitro studies, given

the nature of the experiments being carried out. However, the majority of in vitro experiments were repeated by independent researchers who were initially unaware of the expected outcome.

Reporting for specific materials, systems and methods

We require information from authors about some types of materials, experimental systems and methods used in many studies. Here, indicate whether each material, system or method listed is relevant to your study. If you are not sure if a list item applies to your research, read the appropriate section before selecting a response.

Materials & experimental systems

n/a	Involved in the study
☐	☒ Antibodies
☐	☒ Eukaryotic cell lines
☒	☐ Palaeontology and archaeology
☐	☒ Animals and other organisms
☒	☐ Clinical data
☒	☐ Dual use research of concern
☒	☐ Plants

Methods

n/a	Involved in the study
☒	☐ ChIP-seq
☐	☒ Flow cytometry
☒	☐ MRI-based neuroimaging

Antibodies

Antibodies used

For Lineage Cocktail; CD4 0.3125 µg/mL biotin 553649 H129.19 BD Biosciences (1:1600), CD5 0.625 µg/mL biotin 553019 53-7.3 BD Biosciences (1:800), CD8a 0.625 µg/mL biotin 553029 53 -6.7 BD Biosciences (1:800), CD11b 2.5 µg/mL biotin 553309 M1/70 BD Biosciences (1:200), CD45R/B220 2.5 µg/mL biotin 553086 RA3-6B2 BD Biosciences (1:200), Ter119 10 µg/mL biotin 553672 TER-119 BD Biosciences (1:50), Gr-1/Ly-6G/C 5 µg/mL biotin 553125 RB6-8C5 BD Biosciences (1:100).

For HSPCs and Committed Progenitors; CD117/cKit 2 µg/mL APC 105812 2B8 Biolegend (1:100), Sca-1/Ly-6A 2 µg/mL PE-Cy7 122513 E13-161.7 Biolegend (1:100), Sca-1/Ly-6A 5 µg/mL FITC 122506 E13-161.7 Biolegend (1:100), CD48 0.8 µg/mL PE 103406 103406HM48-1 Biolegend (1:250), CD150 2µg/mL PE-Cy7 115914 12F12.2 Biolegend (1:100), Erythroid Ter119 0.2 µg/mL APC-Cy7 116223 Biolegend (1:1000), CD71 0.2 µg/mL PE 113807 RI7217 Biolegend (1:1000).

For B Cells; CD19 0.2 µg/mL APC-Cy7 115530 6D5 Biolegend (1:1000),CD45R/B220 2 µg/mL PerCP 103236 RA3-6B2 Biolegend (1:100).

For Myeloid Cells; CD11b 0.5 µg/mL PB 101224 M170 Biolegend (1:1000), Gr-1/Ly-6G/C 0.2 µg/mL PE-Cy7 108416 RB6-8C5 Biolegend (1:1000).

For T Cells; CD4 0.2 µg/mL PE 130310 H129.19 Biolegend (1:1000), CD8a 0.08 µg/mL PE 100708 53-6.7 Biolegend (1:2500), CD8a 0.2 µg/mL APC 100712 53-6.7 Biolegend (1:1000).

For Transplantation Assays; CD45.1 0.5 µg/mL FITC 110706 A20 Biolegend (1:1000), CD45.1 0.2 µg/mL BV711 110739 A20 Biolegend (1:1000), CD45.2 0.5 µg/mL Pacific Blue 109820 104 Biolegend (1:1000).

For Streptavidin staining; Streptavidin 2.5 µg/mL BV421 405225 – Biolegend (1:200), Streptavidin 2.5 µg/mL PerCP 405213 – Biolegend (1:200)

Fc Block CD16/32 10 µg/mL none 553142 2.4G2 BD Pharmingen (1:50).

For Western Blot Assays; anti-HIF-1α (BD Biosciences, cat no 610959, 1:1000), anti-GAPDH (Invitrogen, cat no: MA5-15738, 1:1000), Rabbit Anti-mouse IgG (Cell Signalling Technology, #58802, 1:5000), anti-HIF-2α (Cell Signalling Technology, #59973, Clone : D6T8V, 1:1000), anti-BNIP3 (Abcam, EPR4034, 1:2000), anti-β-actin (Cell Signalling Technology, #3700, 1:10,000), anti-Histone-H3 (Cell Signalling Technology, #4499, 1:2000), anti-mouse IgG (Cell Signalling Technology #7076, 1:2000) and anti-rabbit IgG (Cell Signalling Technology, #7074, 1:2000).

Validation

All antibodies chosen were used and validated according to manufacturer's guidelines, as well as being used in numerous publications. All antibodies used in the study have been previously published by the research group (https://doi.org/10.1084/jem.20150452, https://doi.org/10.1084/jem.20161087, https://doi.org/10.1182/bloodadvances.2020002702 and https://doi.org/10.1016/j.stem.2019.03.021.)

Lineage Cocktail:
CD4 https://www.bdbiosciences.com/en-gb/products/reagents/flow-cytometry-reagents/research-reagents/single-color-antibodies-ruo/biotin-rat-anti-mouse-cd4.553649
CD5 https://www.bdbiosciences.com/en-gb/products/reagents/flow-cytometry-reagents/research-reagents/single-color-antibodies-ruo/biotin-rat-anti-mouse-cd5.553019
CD8a https://www.bdbiosciences.com/en-gb/products/reagents/cell-preparation-separation-reagents/biotin-rat-anti-mouse-cd8a.553029
CD11b https://www.bdbiosciences.com/en-gb/products/reagents/flow-cytometry-reagents/research-reagents/single-color-

antibodies-ruo/biotin-rat-anti-cd11b.553309
CD45R/B220 https://www.bdbiosciences.com/en-gb/products/reagents/cell-preparation-separation-reagents/biotin-rat-anti-mouse-cd45r-b220.553086
Ter119 https://www.bdbiosciences.com/en-gb/products/reagents/flow-cytometry-reagents/research-reagents/single-color-antibodies-ruo/biotin-rat-anti-mouse-ter-119-erythroid-cells.553672
Gr-1/Ly-6G/C https://www.bdbiosciences.com/en-gb/products/reagents/cell-preparation-separation-reagents/biotin-rat-anti-mouse-ly-6g-and-ly-6c.553125

HSPCs and Committed Progenitors:
CD117/cKit https://www.biolegend.com/en-us/products/apc-anti-mouse-cd117-c-kit-antibody-72
Sca-1/Ly-6A PE-Cy7 https://www.biolegend.com/en-us/products/pe-cyanine7-anti-mouse-ly-6a-e-sca-1-antibody-3898
Sca-1/Ly-6A FITC https://www.biolegend.com/en-us/products/fitc-anti-mouse-ly-6a-e-sca-1-antibody-3894
CD48 https://www.biolegend.com/en-us/products/pe-anti-mouse-cd48-antibody-293
CD150 https://www.biolegend.com/en-us/products/pe-cyanine7-anti-mouse-cd150-slam-antibody-3056
Ter119 https://www.biolegend.com/en-us/products/apc-cyanine7-anti-mouse-ter-119-erythroid-cells-antibody-3905
CD71 https://www.biolegend.com/en-us/products/pe-anti-mouse-cd71-antibody-1631

B Cells:
CD19 https://www.biolegend.com/en-us/products/apc-cyanine7-anti-mouse-cd19-antibody-3903
CD45R/B220 https://www.biolegend.com/en-us/products/percp-cyanine5-5-anti-mouse-human-cd45r-b220-antibody-4267

Myeloid Cells:
CD11b https://www.biolegend.com/en-us/products/pacific-blue-anti-mouse-human-cd11b-antibody-3863
Gr-1/Ly-6G/C https://www.biolegend.com/en-us/products/pe-cyanine7-anti-mouse-ly-6g-ly-6c-gr-1-antibody-1931

T Cells:
CD4 https://www.biolegend.com/en-us/products/pe-anti-mouse-cd4-antibody-5488
CD8a PE https://www.biolegend.com/en-us/products/pe-anti-mouse-cd8a-antibody-155
CD8a APC https://www.biolegend.com/en-us/products/apc-anti-mouse-cd8a-antibody-150

Transplantation Assays:
CD45.1 FITC https://www.biolegend.com/en-us/products/fitc-anti-mouse-cd45-1-antibody-198
CD45.1 BV711 https://www.biolegend.com/en-us/products/brilliant-violet-711-anti-mouse-cd45-1-antibody-8925
CD45.2 PB https://www.biolegend.com/en-us/products/pacific-blue-anti-mouse-cd45-2-antibody-3108

Streptavidin Staining:
BV421 Streptavidin https://www.biolegend.com/en-us/products/brilliant-violet-421-streptavidin-7297
PerCP Streptavidin https://www.biolegend.com/en-us/products/percp-streptavidin-4211

Fc Block: CD16/32 https://www.bdbiosciences.com/en-gb/products/reagents/flow-cytometry-reagents/research-reagents/single-color-antibodies-ruo/purified-rat-anti-mouse-cd16-cd32-mouse-bd-fc-block.553142

Western Blots:
anti-HIF-1α https://www.bdbiosciences.com/en-gb/products/reagents/microscopy-imaging-reagents/immunofluorescence-reagents/purified-mouse-anti-human-hif-1.610959
anti-GAPDH https://www.thermofisher.com/antibody/product/GAPDH-Loading-Control-Antibody-clone-GA1R-Monoclonal/MA5-15738
Rabbit anti-mouse IgG https://www.cellsignal.com/products/secondary-antibodies/rabbit-anti-mouse-igg-light-chain-specific-d3v2a-mab-hrp-conjugate/58802
anti-HIF-2α https://www.cellsignal.com/products/primary-antibodies/hif-2a-d6t8v-rabbit-mab/59973
anti-BNIP3 https://www.abcam.com/products/primary-antibodies/bnip3-antibody-epr4034-ab109362.html
anti-β-actin https://www.cellsignal.com/products/primary-antibodies/b-actin-8h10d10-mouse-mab/3700
anti-Histone-H3 https://www.cellsignal.com/products/primary-antibodies/histone-h3-d1h2-xp-rabbit-mab/4499
anti-mouse IgG https://www.cellsignal.com/products/secondary-antibodies/anti-mouse-igg-hrp-linked-antibody/7076
anti-rabbit IgG https://www.cellsignal.com/products/secondary-antibodies/anti-rabbit-igg-hrp-linked-antibody/7074

Eukaryotic cell lines

Policy information about cell lines and Sex and Gender in Research

Cell line source(s)	THP-1, Kasumi1, MOLM13, OCI-AML3, MV411 and K562 cell lines are established human AML cell lines purchased during this study (Purchased from ATCC or DKFZ). Plat-E cells used to generate retrovirus were purchased during this study (Purchased from Cell BioLabs). HEK293T cells were used for chemistry based studies for PHD inhibitors .(Purchased from ATCC). WT and KO iMLL-AF9 and WT and KO Meis1Hoxa9 cells were generated during this study from primary mouse tissue.
Authentication	Human AML cell lines were STR typed at regular intervals during the project. Plat-Es were regularly re-selected via antibiotic resistance. Murine cell lines generated during this project were regularly tested for expression of the oncogene as well as validation of the appropriate knockout.
Mycoplasma contamination	Cell lines tested negative for mycoplasma contamination at 3 month intervals.

Commonly misidentified lines (See ICLAC register)	None

Animals and other research organisms

Policy information about studies involving animals; ARRIVE guidelines recommended for reporting animal research, and Sex and Gender in Research

Laboratory animals	All mice were on the C57BL/6 genetic background. Phd2fl/fl, shPhd2 and iMLL-AF9 mice were previously published and validated. Vav-iCre, and NBSGW mice were purchased from the Jackson Laboratory. All transgenic and knockout mice were CD45.2+. Congenic recipient mice were CD45.1+/CD45.2+. Mice used for support BM cells during transplantation experiments were CD45.1+. Mice used were between 8-12 weeks old, unless otherwise stated in the figure legends. All animals were subject to an optimum dark/light cycle, with ambient temperature and humidity.
Wild animals	No wild animals were used in this study.
Reporting on sex	All experiments were performed on mixed gender animals.
Field-collected samples	No field-collected samples were used in this study.
Ethics oversight	All experiments on animals were performed under UK Home Office authorisation under the project license PP4153210 at Barts Cancer Institute following approval by Queen Mary University of London AWERB.

Note that full information on the approval of the study protocol must also be provided in the manuscript.

Flow Cytometry

Plots

Confirm that:

☒ The axis labels state the marker and fluorochrome used (e.g. CD4-FITC).

☒ The axis scales are clearly visible. Include numbers along axes only for bottom left plot of group (a 'group' is an analysis of identical markers).

☒ All plots are contour plots with outliers or pseudocolor plots.

☒ A numerical value for number of cells or percentage (with statistics) is provided.

Methodology

Sample preparation	Cells were harvested and washed in PBS 1x prior to staining and were stained as described in the methods section
Instrument	Analysis was performed on the LSRFortessa (BD). Cell sorting was performed on a FACSAria Fusion (BD).
Software	FlowJo v10 6.1
Cell population abundance	For sorted cell populations, LSK cells have an approximate abundance of 1.5% and HSCs have an approximate abundance of 0.1% of total BM cells.
Gating strategy	All gating strategies are provided in Supplementary Information. All experiments are first gated on a "lymphocyte gate" using FSC-A vs SSC-A, followed by a "single cell gate" using FSC-A vs FSC-H. Sorted samples used a further "single cell gate" from SSC-A vs SSC-W. All experiments were analysed using single stain controls.

☒ Tick this box to confirm that a figure exemplifying the gating strategy is provided in the Supplementary Information.
