# Supplementary material for: The selective prolyl hydroxylase inhibitor IOX5 stabilizes HIF-1α and compromises development and progression of acute myeloid leukemia
Source: Nat Cancer. 2024 Apr 18;5(6):916–37. doi: 10.1038/s43018-024-00761-w (PMC11208159; doi:10.1038/s43018-024-00761-w)
Supplement: Supplementary file 18 — Unprocessed western blot gels. [file 43018_2024_761_MOESM18_ESM.pdf]

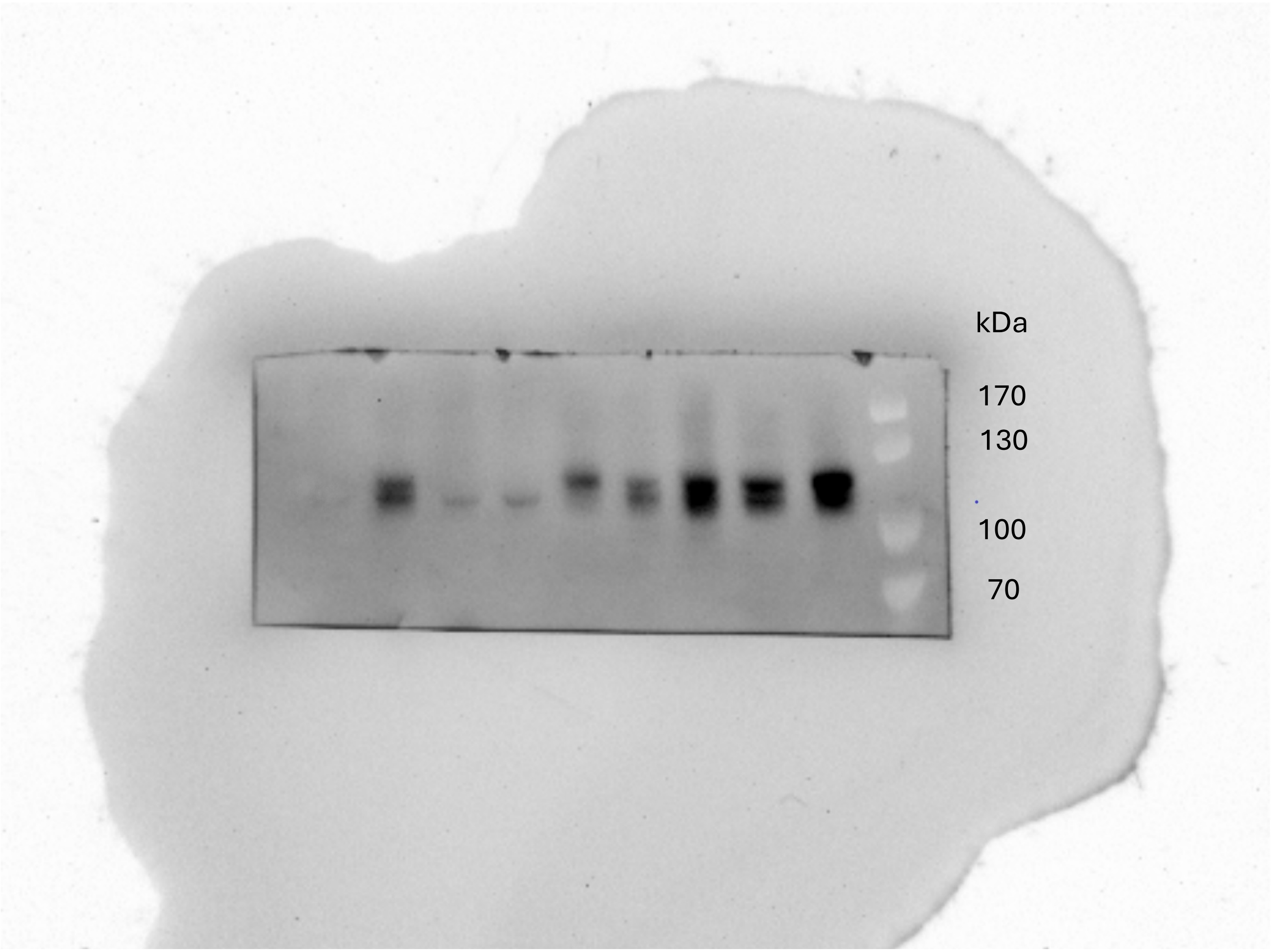

Fig. 4d – HIF-1α

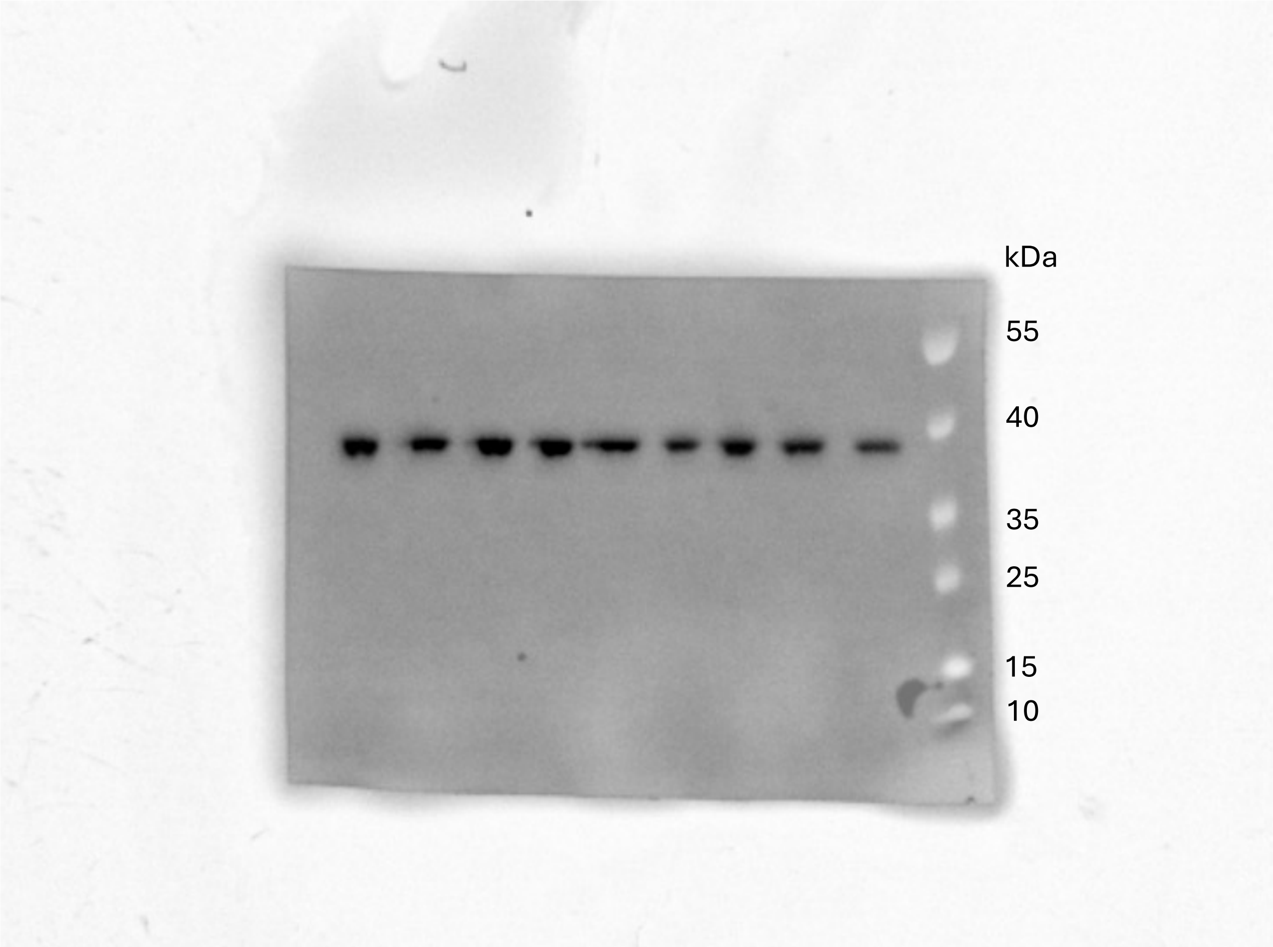

Fig. 4d – GAPDH

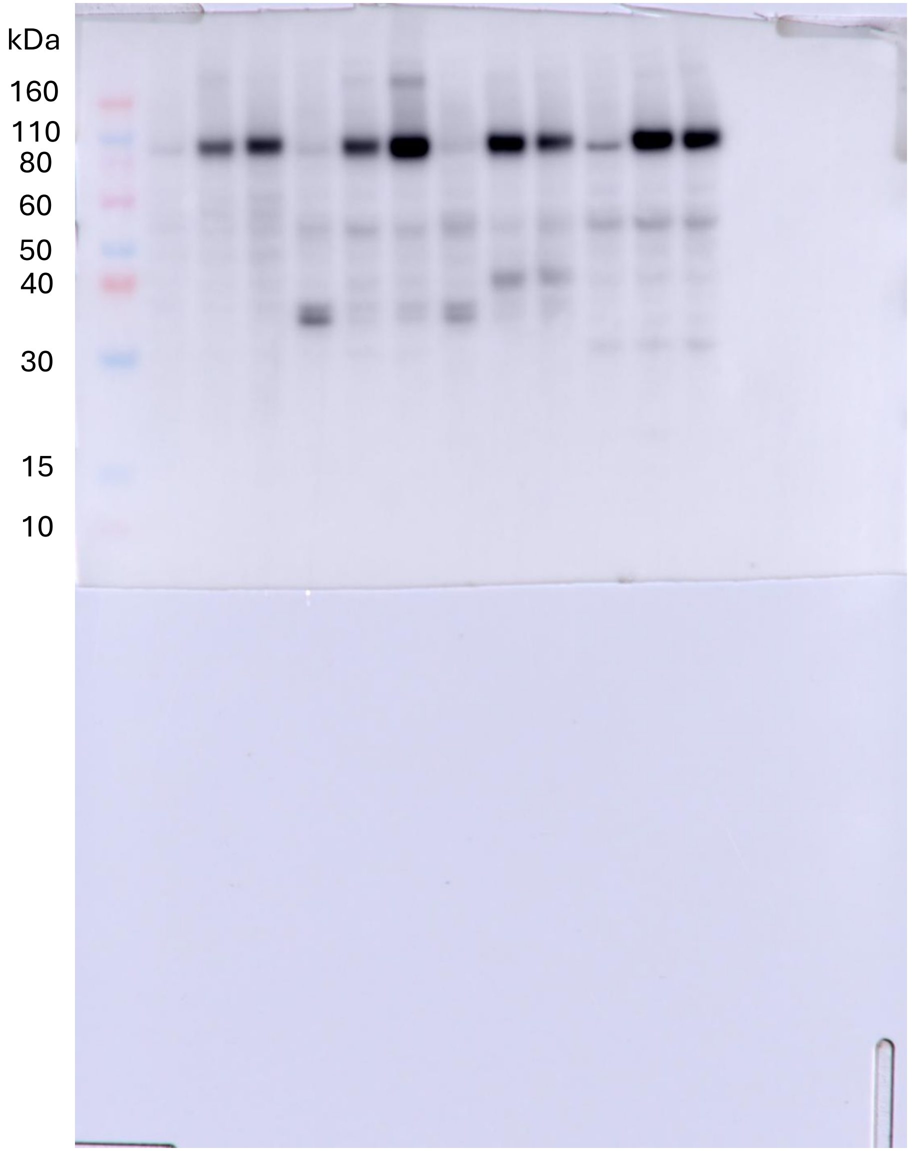

Fig. 5a – HIF-1α

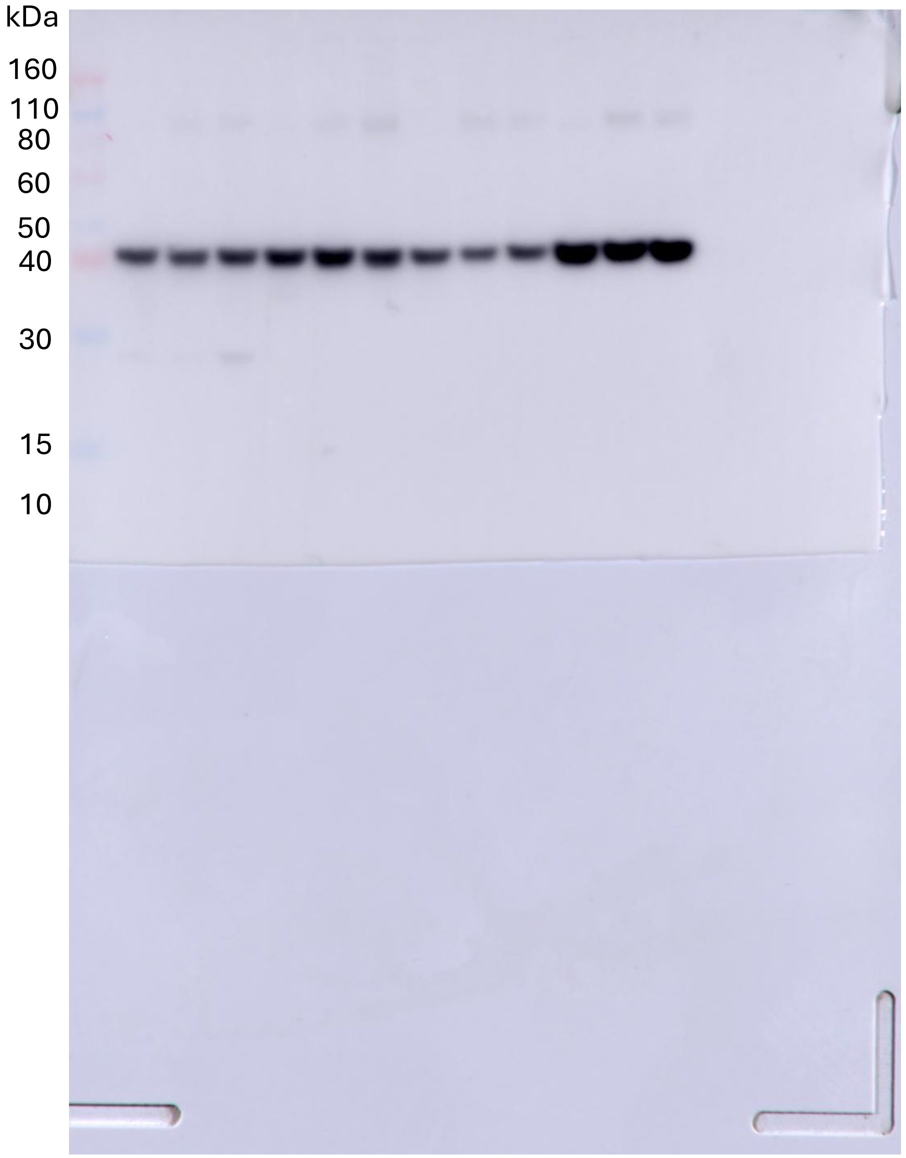

Fig. 5a – β-actin

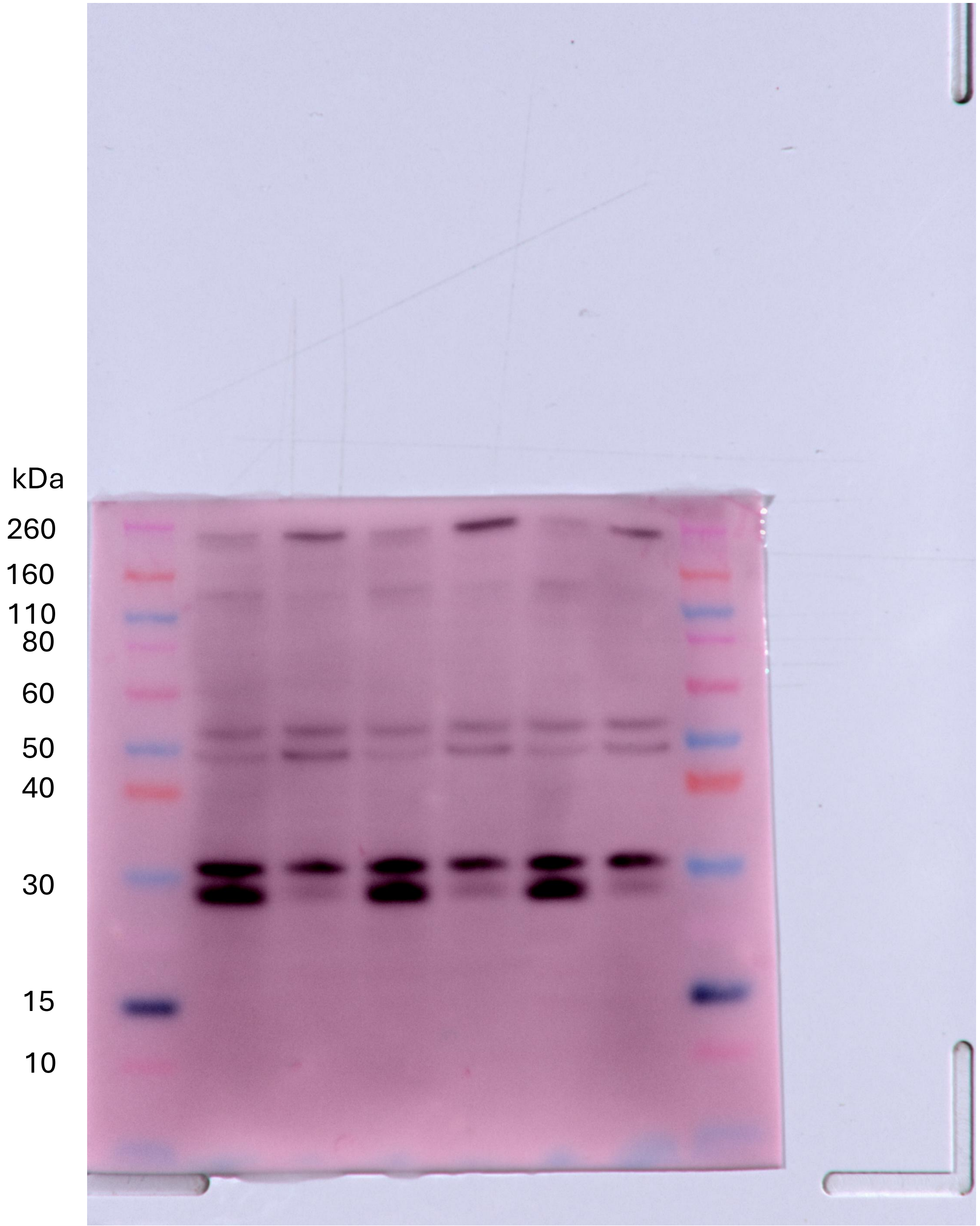

Fig. 7f – BNIP3

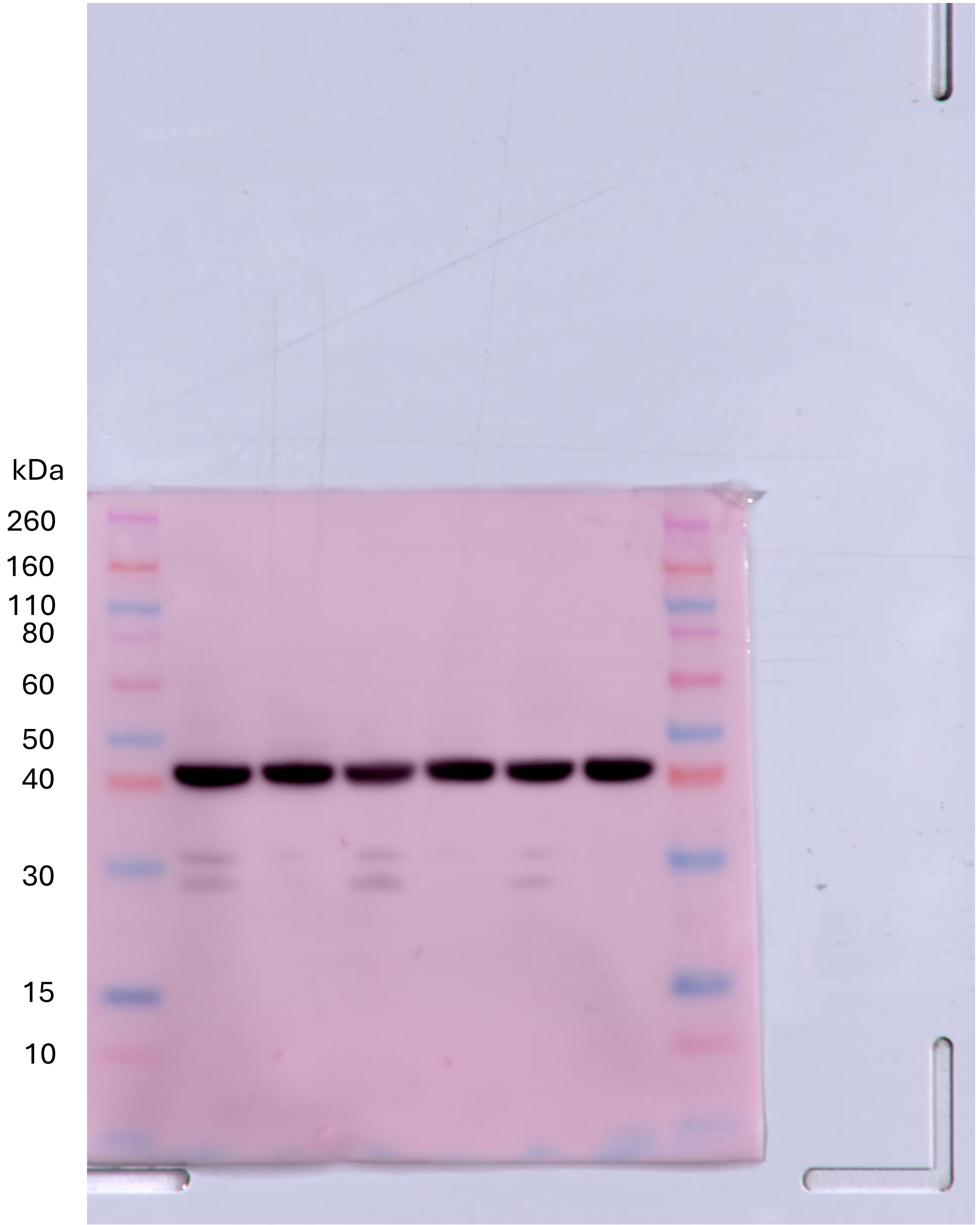

Fig. 7f – β-actin

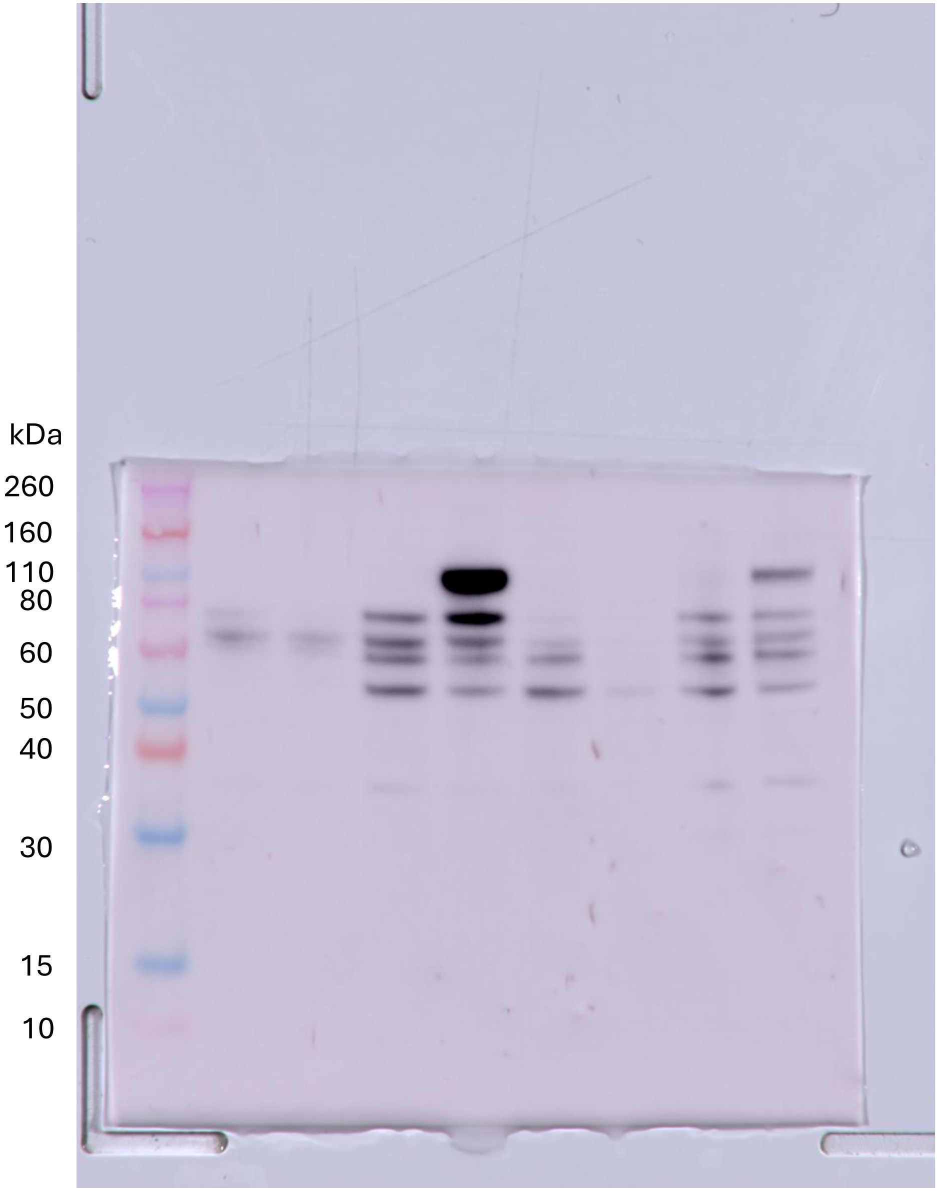

Extended Data Fig. 5a – HIF-2α

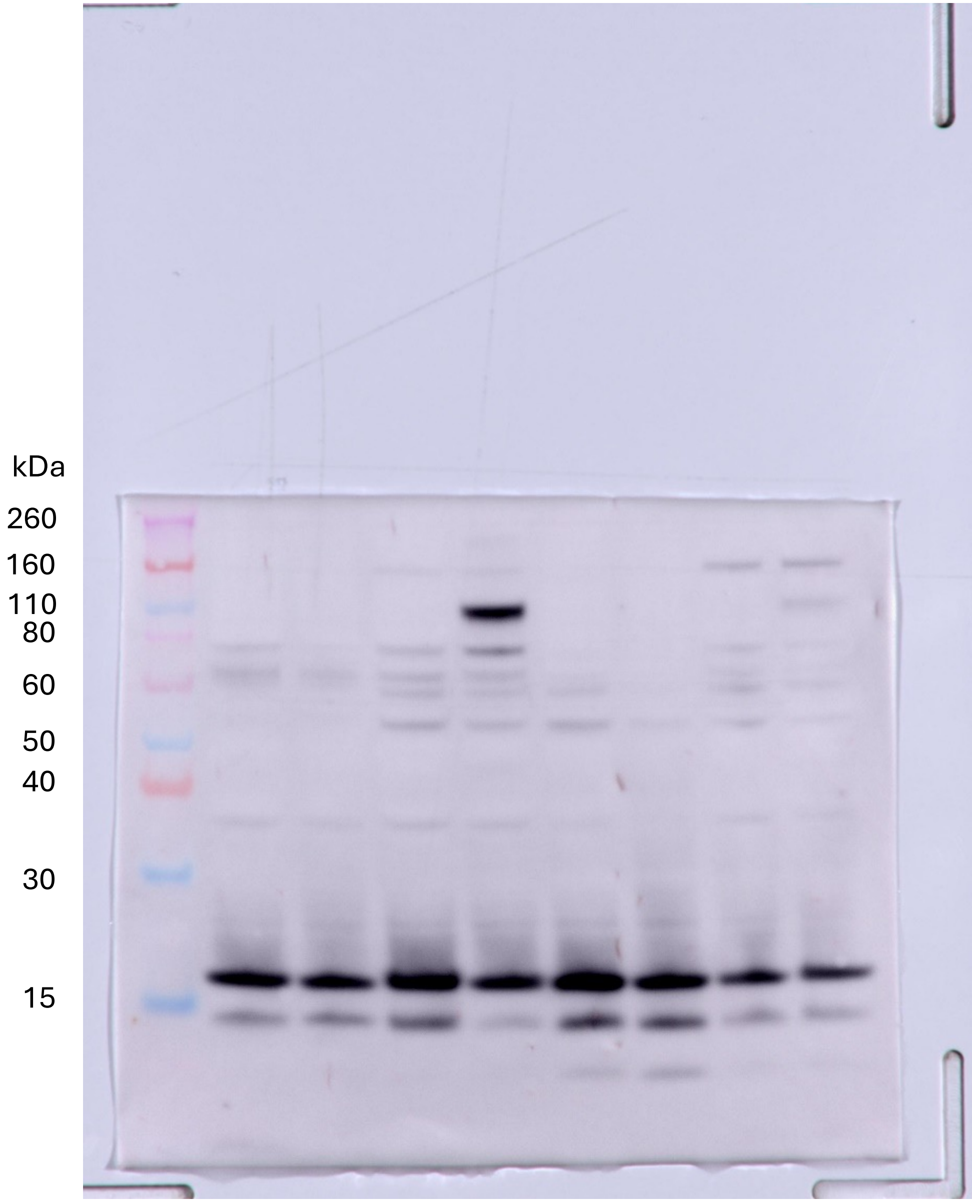

Extended Data Fig. 5a – Histone H3

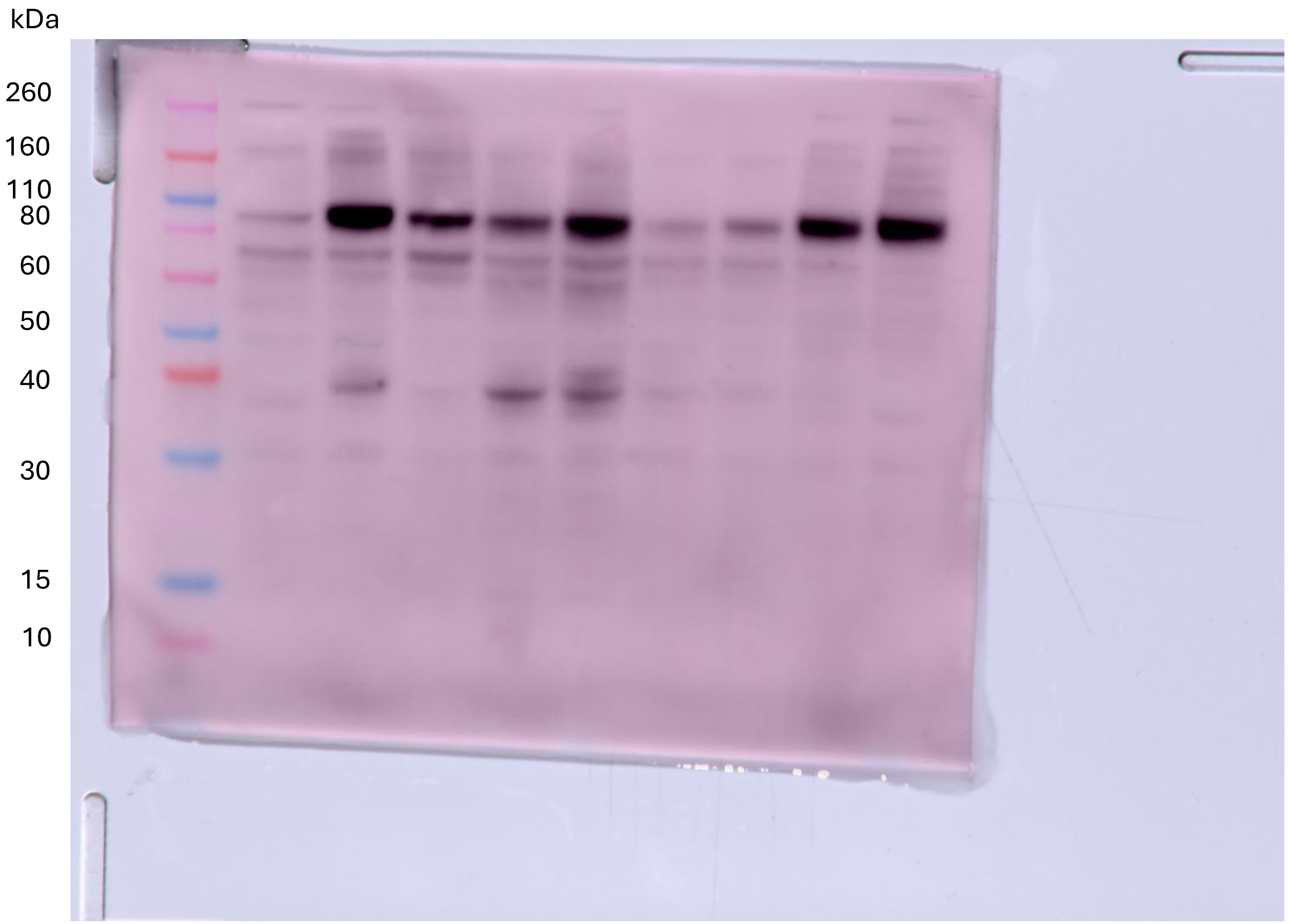

Extended Data Fig. 5f – HIF-1α

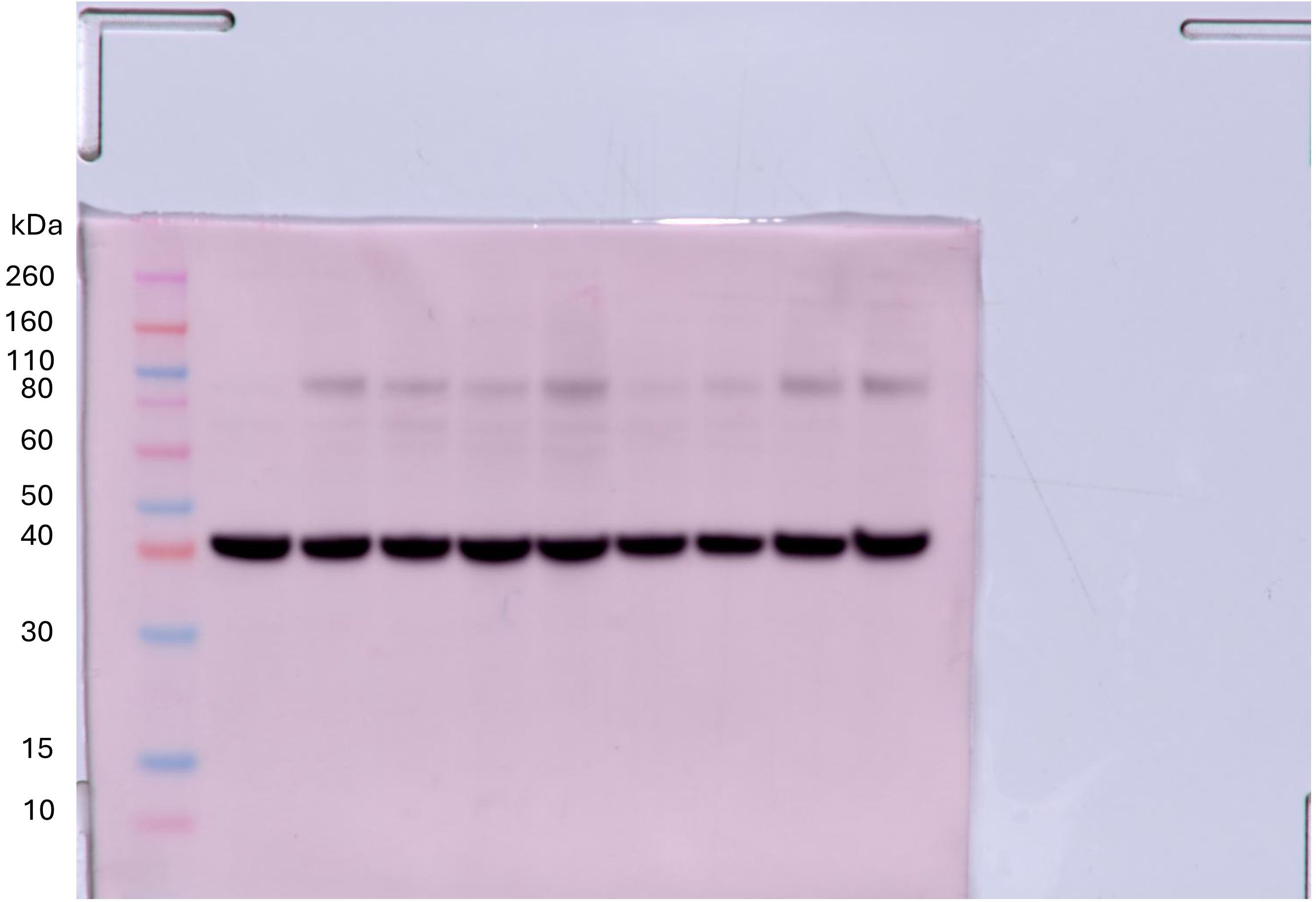

Extended Data Fig. 5f – β-actin
